# Effect of Chemical Mutagens and Carcinogens on Gene Expression Profiles in Human TK6 Cells

**DOI:** 10.1371/journal.pone.0039205

**Published:** 2012-06-18

**Authors:** Lode Godderis, Reuben Thomas, Alan E. Hubbard, Ali M. Tabish, Peter Hoet, Luoping Zhang, Martyn T. Smith, Hendrik Veulemans, Cliona M. McHale

**Affiliations:** 1 Occupational, Environmental and Insurance Medicine, Katholieke Universiteit Leuven, Leuven, Belgium; 2 External Service for Prevention and Protection at Work, Idewe, Heverlee, Belgium; 3 Division of Environmental Health Sciences, School of Public Health, University of California, Berkeley, California, United States of America; 4 Division of Biostatistics, School of Public Health, University of California, Berkeley, California, United States of America; University of Chicago, United States of America

## Abstract

Characterization of toxicogenomic signatures of carcinogen exposure holds significant promise for mechanistic and predictive toxicology. *In vitro* transcriptomic studies allow the comparison of the response to chemicals with diverse mode of actions under controlled experimental conditions. We conducted an *in vitro* study in TK6 cells to characterize gene expression signatures of exposure to 15 genotoxic carcinogens frequently used in European industries. We also examined the dose-responsive changes in gene expression, and perturbation of biochemical pathways in response to these carcinogens. TK6 cells were exposed at 3 dose levels for 24 h with and without S9 human metabolic mix. Since S9 had an impact on gene expression (885 genes), we analyzed the gene expression data from cells cultures incubated with S9 and without S9 independently. The ribosome pathway was affected by all chemical-dose combinations. However in general, no similar gene expression was observed among carcinogens. Further, pathways, i.e. cell cycle, DNA repair mechanisms, RNA degradation, that were common within sets of chemical-dose combination were suggested by clustergram. Linear trends in dose–response of gene expression were observed for Trichloroethylene, Benz[a]anthracene, Epichlorohydrin, Benzene, and Hydroquinone. The significantly altered genes were involved in the regulation of (anti-) apoptosis, maintenance of cell survival, tumor necrosis factor-related pathways and immune response, in agreement with several other studies. Similarly in S9+ cultures, Benz[a]pyrene, Styrene and Trichloroethylene each modified over 1000 genes at high concentrations. Our findings expand our understanding of the transcriptomic response to genotoxic carcinogens, revealing the alteration of diverse sets of genes and pathways involved in cellular homeostasis and cell cycle control.

## Introduction

Cancer is a multifactorial disease in which both environmental and genetic factors play a role. Thus far, the cancer risk associated with exposure to industrial pollutants has been mainly assessed through epidemiological follow-up, animal studies, mutagenicity and genotoxicity assays. Exposure-related cancers are difficult to detect in epidemiological studies given that the induced cancer risk is small compared to the natural occurrence of cancer [1], [2], [3], [4], [5] and latencies can be protracted. Despite the possibility of measuring genotoxic effects in humans, e.g. DNA breaks and micronuclei, which can lead to the development of a cancer, the interpretation of positive findings in relation to exposure and cancer risk assessment remains a major challenge [6]. With the development of new tests, it is hoped that more effective bioassay surrogates will produce clinical tools for monitoring and risk assessment [7], [8], [9], [10], [11].

Collaborative efforts have been taken to deal with the health effects of large number of chemicals released in the environment and to develop cost-effective high-throughput approaches to assess environmental chemical toxicity [12]. In recent years, ‘omic’ technologies have been employed to get a deeper understanding of toxicological mechanisms and to predict toxicological outcome, such as carcinogenicity, by profiling genomic perturbations (e.g., mRNA expression profile) [13], [14], [15]. Several promising studies have been reported in mechanistic [16], [17] and predictive toxicology [18], [19], [20] using toxicogenomics. Toxicogenomic tools have also been utilized to discriminate between classes of carcinogens based on global expression profiling [19], [21]. This suggests that for compounds with insufficient toxicological information, associated gene signatures could be used to characterize their toxicological properties based on comparison with signatures associated with previously characterized compounds. Studies have reported positive findings regarding the toxicogenomic strategy of compound categorization based on the mode of action (e.g., gene expression alterations) [22] but data is still too preliminary to support the conclusion that similar gene expression signatures are observed among families of structurally similar compound(s) and/or compounds with similar mode of action [23], [24].

Human toxicogenomic studies typically are small, examine high dose exposures, have confounding issues (such as age, genotype, and diet) and use various methodologies, all of which make it difficult to compare the effects of exposure to different chemicals or classes of chemicals among studies. In contrast, *in vitro* toxicogenomic studies can be designed to examine the effect of multiple exposures in parallel with greatly reduced confounding. *In vitro* gene expression profiling has the potential to discriminate carcinogens with distinct mechanisms of action [25]. Several *in vitro* studies have shown that genotoxic agents alter genes involved in immune, inflammatory and stress responses and apoptosis, rather than genes with a role in DNA repair or metabolism, as might have been expected [26], [27], [28], [29], [30], [31]. These studies examined genotoxins that operate through various mechanisms, in human and mouse cell lines using different microarray platforms.

In the current study, we characterized the gene expression alterations in TK6 cells induced by by genotoxic carcinogens with diverse modes of action e.g., adduct forming and cross-linking. We also examined the dose-responsive changes in gene expression and altered biochemical pathways. We hypothesized that the gene expression signatures are perturbed in response to exposure in a carcinogen, and a dose specific manner. The carcinogens comprised 13 chemicals currently and frequently used in European industries, including occupational and environmental agents, as well as 2 control chemicals (Table 1). These genotoxic agents react with/alter DNA and/or proteins directly or indirectly (through their bioactivation) [32].

**Table 1:** Overview of agents used for the treatment of TK6 cell cultures.

Agents	IARC	Category	Concentration (µM)		
			High	Medium	Low
Formaldehyde*,1,2	1	Aldehyde	100	10	1
Styrene^**,1^	2B	Aromatic hydrocarbon	5000	500	50
Styrene 7,8-oxide*,1	2A	Aromatic hydrocarbon	500	50	5
Benzene^**,1^	1	Aromatic hydrocarbon	100	10	1
Hydroquinone*,1	3	Aromatic hydrocarbon	0.5	0.05	0.005
Mitomycin C*,2	2B	Cytostaticum	0.5	0.05	0.005
Ethylenedibromide^**,1,2^	2A	Organobromide	1000	100	10
Epichlorohydrin*,1	2A	Organochloride	500	50	5
Acrylamide^**,1^	2A	Amide	500	50	5
Trichloroethylene^**,1^	2A	Chlorinated hydrocarbon	5000	500	50
Carbontetrachloride^**,1^	2B	Chlorinated hydrocarbon	1000	100	10
Cyclophosphamide^**,1^	1	Cytostaticum	50	5	0.5
Benzo[a]fluoranthene^**,1^	2B	Poly aromatic hydrocarbon	500	50	5
Benzo[a]pyrene^**,1^	1	Poly aromatic hydrocarbon	500	50	5
Benz[a]anthracene^**,1^	2B	Poly aromatic hydrocarbon	500	50	5

* Direct acting agent; **Indirect acting agent,

1: DNA adduct forming agent; 2: DNA Cross linking agent.

Human thymidine kinase ± human lymphoblastoid cells (TK6 cells) were exposed for 24 hours and global gene expression profiling was performed. In order to assess the applicability of gene expression profiles to biomonitoring of human populations exposed to carcinogens, we also examined dose-dependent changes in gene expression. We applied a long exposure period (24 h) rather than a pulse treatment (4–6 h), since humans (e.g. workers) are typically chronically exposed and in *in vitro* studies, 18 to 24 h exposure period is commonly used for investigation of genotoxicity [33], [34]. Moreover, it has been shown that more changes in gene expression with alteration of more genes, occurs after approximately 24 h than after 4, 48, or 72 h [27], [28], [29], [30], [35].

## Results

After 24 h of exposure, cells were harvested and analyzed for viability. All samples included in this study showed viability above 90% and were further processed for RNA extraction. The RNA from each exposure condition was hybridized to the Sentrix HumanRef-8 v33 Expression BeadChips (Illumina, Inc., San Diego, CA).

### Assessment of experimental variation

**Figure 1:**
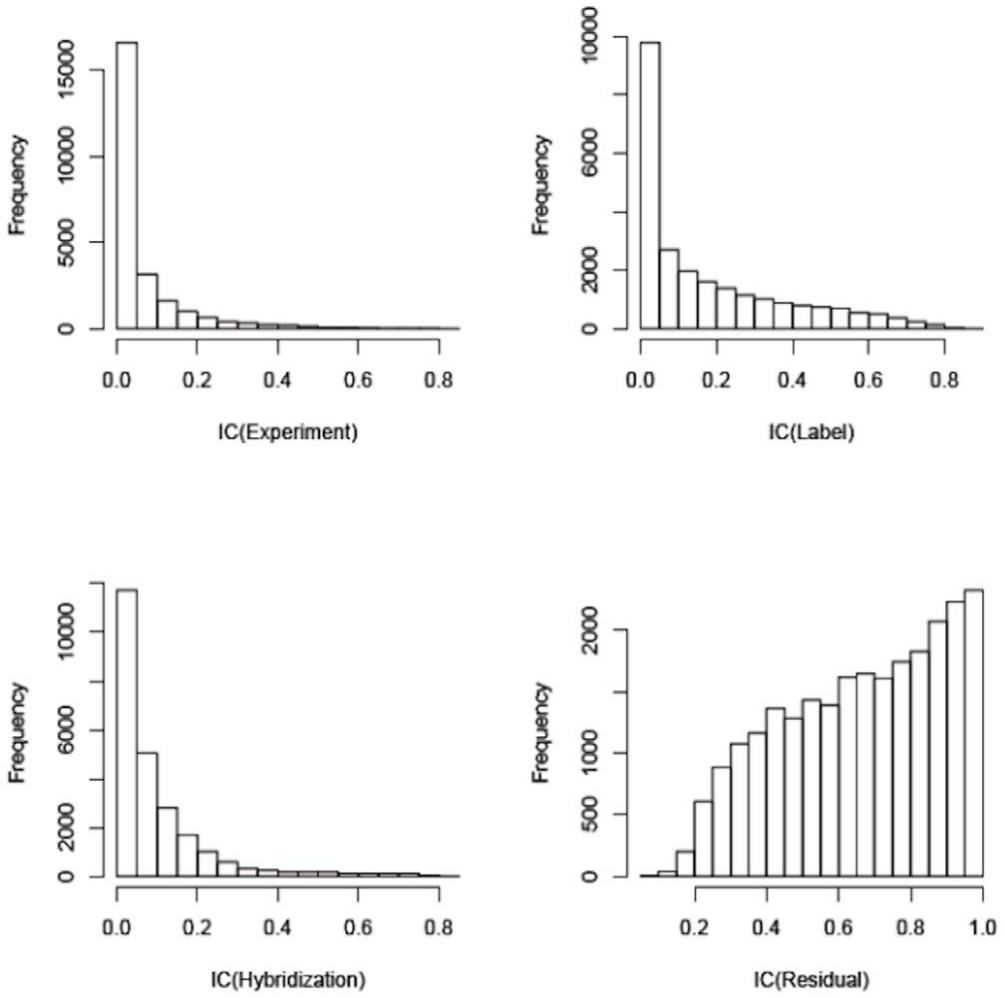
Sources of methodological and biological variation within the microarray expression data. Figure represents the distribution of the intraclass correlation coefficients (the proportion of variability estimated to come from each source on a probe-by-probe basis) calculated by variance components analysis based on a mixed-effects model allowing assessment of independent contributions of variability from experiment, label, hybridization, and residual variability. IC: intra-class correlation coefficients; x-axis represent the scale of IC (0–1) and y-axis represent the frequency of IC.

We applied a linear mixed model to assess the proportion of total variation that is due to exposure (chemical, S9, dose) and experimental variation (treatment experiment, hybridization, label and chip). The distribution by gene of the IntraClass Correlation Coefficients for 3 of the potential experimental confounding factors (experiment, label, and hybridization) and residual showed that residual variation was greatest (Figure 1). This means that exposure, i.e. chemical agent, S9 and dose had the strongest effect on variation in gene expression. We adjusted the inference for correlation due to labeling and hybridization, which had lesser effects on variation than exposure.

### Effect of S9 treatment on gene expression

**Figure 2:**
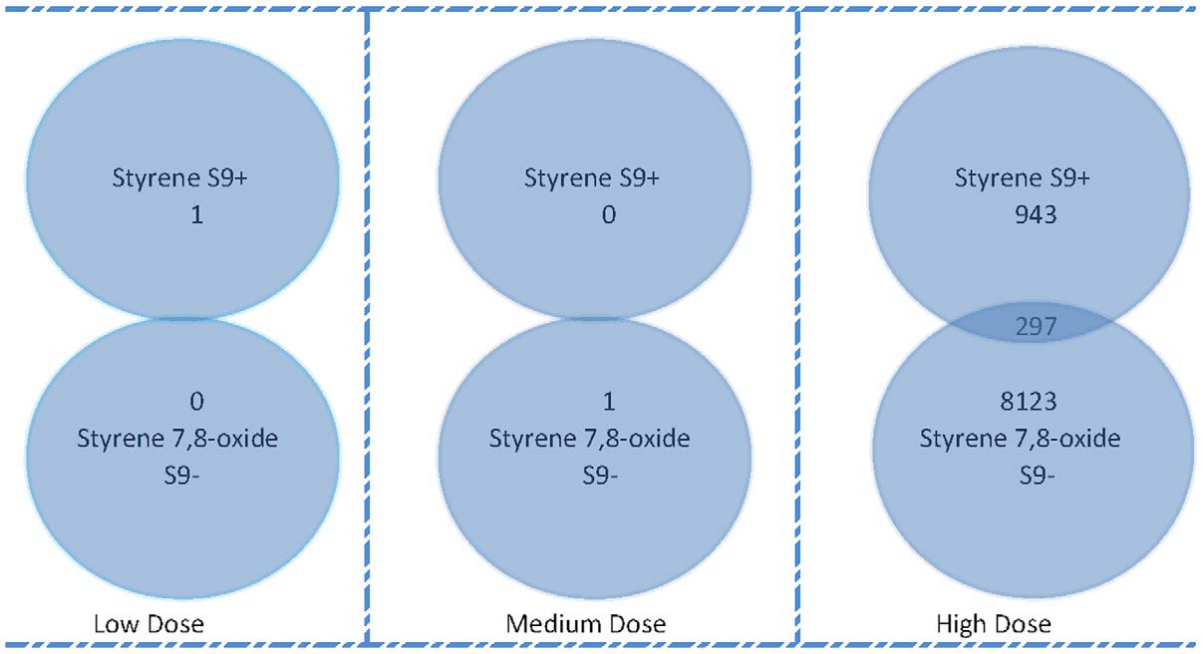
Genes in common between Styrene (S9+) and Styrene 7,8-oxide (S9-). Venn diagram indicating the number of genes common between styrene (S9+) and styrene 7,8-oxide (S9-) at low, medium and high dose (*q*-value<0.15) exposed TK6 cells. The number of overlapping genes at each of the dose levels were not significant (*q*-value<0.15) by Fisher's exact test.

In order to determine the effectiveness of S9 activation in our treatment experiments, we compared the differential gene expression profiles of cells treated with Styrene, which requires metabolic activation, in the presence of S9, with the profiles associated with its major metabolite, Styrene 7,8-oxide, in the absence of S9. Styrene and Styrene 7,8-oxide had 297 genes (*q*-value<0.15) in common at high dose treatment, and no genes in common at low and medium doses (Figure 2). The high-dose result, suggested that S9 affected the metabolism of Styrene to Styrene 7,8-oxide. However, the degree of overlap was small suggesting that metabolism by S9 was incomplete, or that Styrene alters gene expression independently of its metabolism or via other metabolites, or that S9 treatment was causing “non-target” effects on genes expression. Comparison of the gene expression patterns of S9-treated (S9+) with S9-untreated (S9-) control samples showed that 885 genes (supplementary Table S1) were significantly altered (*q*-value<0.01), of which 375 genes were down regulated. This confirmed that “non-target” effects of S9 treatment occurred. Given these non-target S9 effects on gene expression, we analyzed the gene expression data from cells cultures incubated without S9 metabolic mix independently from those incubated with S9 metabolic mix.

### GO categories and genes significantly impacted by individual carcinogens vs controls

We examined the genes modulated by each agent at each concentration. After multiple test correction (False Discovery Rate (FDR) *q*-value <0.15), many exposure-induced gene expression modifications remained significant. At low-dose exposure, Formaldehyde significantly altered the expression of 3 genes (FLJ44653 and SFRS11 were upregulated, CTBP1 was downregulated); Mitomycin C exposure significantly altered the expression of 21 genes of which 3 were downregulated; and, Acrylamide significantly altered the expression of 61 genes. Low-dose exposure to Benz[a]anthracene, Benzo[a]pyrene, Carbontetrachloride, Cyclophosphamide, Hydroquinone, Trichloroethylene and Styrene also affected gene expression. At the medium dose level, various numbers of genes were significantly altered (*q*-value <0.15): Cyclophosphamide (134 genes), Carbontetrachloride (353 genes), Benz[a]anthracene (397 genes), Trichloroethylene (385 genes), Epichlorohydrin (826 genes) and Benzene (>1000 genes). Similarly, at the high-dose level, various numbers of genes were significantly altered (*q*-value <0.15): Styrene (7 genes), Acrylamide (81 genes), Benzene (254 genes), Hydroquinone (332 genes), Ethylenedibromide (748 genes) and Styrene 7,8-oxide (>1000 genes), Trichloroethylene (>1000 genes), Benz[a]anthracene (>1000 genes) and Epichlorohydrin (>1000 genes) (*q*-value <0.15).

The biological processes impacted by each chemical (S9-) at each dose level were determined by GO analysis and are summarized in supplementary Table S2. GO categories which were affected by at least 4 chemicals per dose level are shown in Table 2 (low dose), Table 3 (medium dose) and Table 4 (high dose). A number of GO processes including antigen processing and presentation of endogenous peptide antigen via MHC class I, peptide antigen stabilization, DNA damage response, and signal transduction by p53 class mediator resulting in cell cycle arrest, were affected at the low dose by most chemicals. Regulation of translational fidelity, positive regulation of epithelial cell differentiation, antigen processing, and presentation of endogenous peptide antigen via MHC class I were significantly affected at the medium and high doses. At the high dose multiple chemicals were associated with perturbation of regulation of cell redox homeostasis, cell cycle arrest, inhibition of CREB transcription factor, and mRNA transcription from RNA polymerase I. Additional GO processes of interest, impacted at medium and high levels of exposure, were positive regulation of tumor necrosis factor receptor, T cell proliferation, selection and costimulation and negative regulation of type IV hypersensitivity, positive regulation of DNA repair, nucleotide-excision repair, single strand break repair, double-strand break repair via homologous recombination, DNA double-strand break processing apoptosis, cell cycle arrest, programmed cell death and apoptotic nuclear change (supplementary Table S2). In the supplementary Table S3, we also provide the GO categories impacted by each chemical (S9+) at each dose level.

**Table 2:** Functional classification of affected genes at low dose.

GO ID	GO Processes	Carcinogens*									
		AA	BA	BP	CCL	CP	FA	HQ	MMC	ST	TCE
GO:0009440	Cyanate catabolic process	ü	ü	O	O	O	O	O	ü	ü	O
GO:0019885	Antigen processing and presentation of endogenous peptide antigen via MHC class I	ü	O	O	ü	O	ü	ü	O	ü	ü
GO:0000085	G2 phase of mitotic cell cycle	O	ü	O	ü	ü	ü	ü	ü	O	ü
GO:0006977	DNA damage response, signal transduction by p53 class mediator resulting in cell cycle arrest	O	ü	O	ü	ü	O	O	O	O	ü
GO:0050823	Peptide antigen stabilization	O	O	ü	ü	O	ü	ü	O	ü	ü
GO:0001833	Inner cell mass cell proliferation	O	O	O	O	ü	ü	ü	O	ü	O

Functional classification of significantly affected genes by exposure to carcinogens at low dose into Gene Ontology (GO) categories was performed. GO categories that were affected by 4 or more carcinogens per chemical dose are listed. A list of all GO categories affected per chemical per dose is given in the supplementary Table S2.

* Carcinogens [AA:Acrylamide; BA:Benz[a]anthracene; BP:Benzo[a]pyrene; CCL:Carbontetrachloride; CP:Cyclophosphamide; FA:Formaldehyde; HQ:Hydroquinone; MMC:Mitomycin C; ST; Styrene; TCE:Trichloroethylene].

**Table 3:** Functional classification of affected genes at medium dose.

GO ID	Go Processes	Carcinogens*					
		BA	BZ	CCL	CP	EPI	TCE
GO:0019885	Antigen processing and presentation of endogenous peptide antigen via MHC class I	P	P	O	P	P	P
GO:0001711	Endodermal cell fate commitment	P	O	P	O	P	P
GO:0006450	Regulation of translational fidelity	P	P	P	O	P	P
GO:0030858	Positive regulation of epithelial cell differentiation	P	O	P	O	P	P

Functional classification of significantly affected genes by exposure to carcinogens at medium dose into Gene Ontology (GO) categories was performed. GO categories that were affected by 4 or more carcinogens per chemical dose are listed. A list of all GO categories affected per chemical per dose is given in the supplementary Table S2.

* Carcinogens [BA:Benz[a]anthracene; BZ:Benzene; CCL:Carbontetrachloride; CP:Cyclophosphamide; EPI:Epichlorohydrin; TCE:Trichloroethylene].

**Table 4:** Functional classification of affected genes at high dose.

GO ID	GO Processes	Carcinogens*								
		AA	BA	BZ	EDB	EPI	HQ	SO	ST	TCE
GO:0030503	Regulation of cell redox homeostasis	P	P	O	P	O	O	O	P	O
GO:0042789	mRNA transcription from RNA polymerase I	P	O	P	P	P	P	O	O	O
GO:0007050	Cell cycle arrest	P	O	P	O	P	P	O	P	O
GO:0032792	Inhibition of CREB transcription factor	P	O	O	P	O	P	O	P	O
GO:0043065	Positive regulation of apoptosis	P	O	P	O	P	O	O	P	O
GO:0001975	Response to amphetamine	P	O	P	O	O	P	O	P	O
GO:0001711	Endodermal cell fate commitment	O	P	O	O	P	P	O	O	P
GO:0006450	Regulation of translational fidelity	O	P	O	O	P	P	O	O	P
GO:0015855	Pyrimidine transport	O	P	O	O	P	O	P	O	P
GO:0030858	Positive regulation of epithelial cell differentiation	O	P	O	O	P	P	O	O	P

Functional classification of significantly affected genes by exposure to carcinogens at high dose into Gene Ontology (GO) categories was performed. GO categories that were affected by 4 or more carcinogens per chemical dose are listed. A list of all GO categories affected per chemical per dose is given in the supplementary Table S2.

* Carcinogens [AA:Acrylamide; BA:Benz[a]anthracene; BZ:Benzene; EDB:Ethylenedibromide; EPI:Epichlorohydrin; HQ:Hydroquinone; SO:Styrene 7,8-oxide; ST:Styrene; TCE:Trichloroethylene].

**Figure 3:**
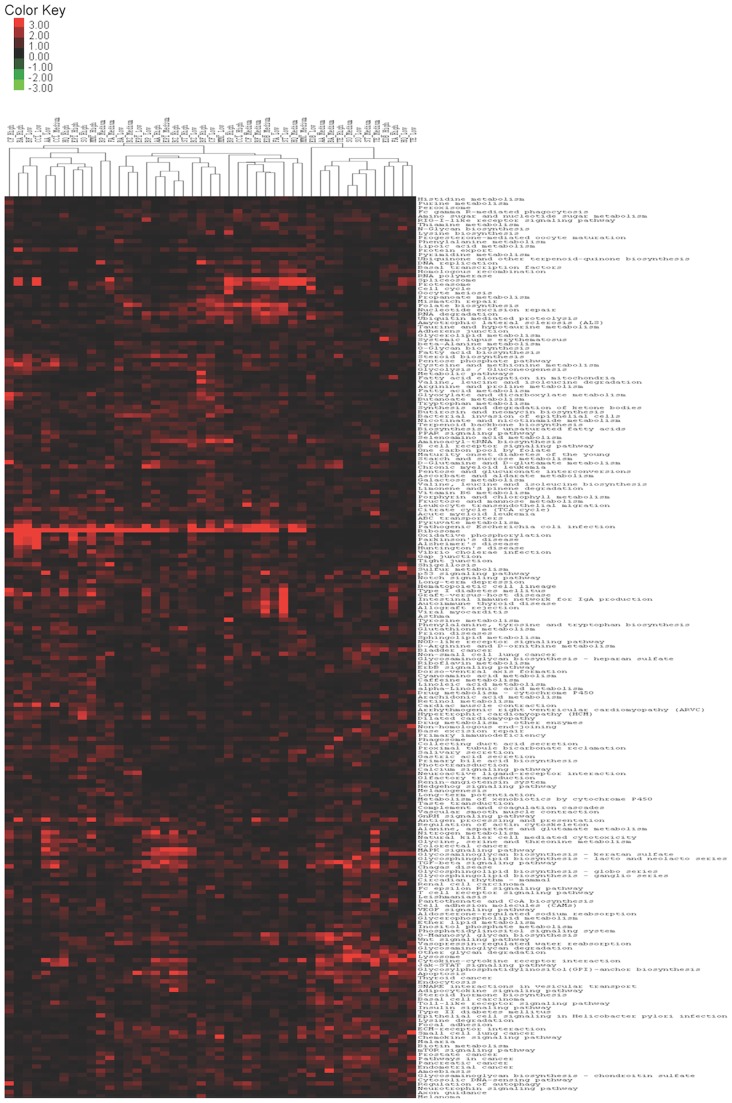
Heatmap generated by hierarchical clustering of chemical-dose arrays. Heatmap representation of the hierarchical trees of pathways and differentially expressed genes in each chemicals-dose combination generated using hierarchical clustering with the Euclidean distance metric and average linkage method. The columns in the Heatmap correspond to each chemical-dose combination and rows correspond to different KEGG human pathways. Significantly impacted genes from the respective biological replicates per chemical were pooled in the hierarchical clustering.

Hierarchical clustering, with heatmap presentation to visualize the pathways that behave similarly and/or differently within each chemical-dose combination (S9-), is shown in Figure 3. Significantly impacted genes from the respective biological replicates per chemical were pooled in the hierarchical clustering. Specific pathways associated with cell cycle alterations, DNA repair i.e., mismatch repair, nucleotide excision repair, spliceosome, proteasome, ribosome, RNA degradation, and others are enriched within sets of chemical-dose combinations as shown in Figure 3. Interestingly, the Ribosome pathway was affected by all chemical-dose combinations used in this study. In general, we did not observe pathways that have similar expression among all direct or indirect- acting carcinogens.

### Dose-dependent changes in gene expression induced by individual carcinogens

Trend analysis of genes impacted by chemicals with S9- treatment revealed dose-dependent changes in gene expression for Benzene (20 genes), Hydroquinone (34 genes), Benz[a]anthracene (8 genes), Trichloroethylene (1000 genes) and Epichlorohydrin (1000 genes) at *q*-value <0.15. We performed GO analysis for the significantly affected genes and the altered GO categories are given in supplementary Table S4. Some of the significantly affected genes (S9-) were involved in DNA repair (e.g. GO:0033683: nucleotide-excision repair, GO:0006283: transcription-coupled nucleotide-excision repair), regulation of cell cycle (e.g. GO:0000075: cell cycle checkpoint, GO:0007050: cell cycle arrest), regulation of apoptosis (e.g. GO:0008624: induction of apoptosis by extracellular signals, GO:0042981: regulation of apoptosis), regulation of immune response (e.g. GO:0001808 negative regulation of type IV hypersensitivity, GO:0042113: B cell activation), regulation of translation (e.g. GO:0006412: translation, GO:0006446: regulation of translational initiation) and GO:0042535: positive regulation of tumor necrosis factor biosynthetic process.

A closer look at the genes involved in regulation of tumor necrosis factors receptors revealed that TNFRSF9 (CD137) and TNFRSF10B (CD262), both members of the tumor necrosis factor receptor family, were upregulated with increasing Epichlorohydrin dose. Interestingly the TNFRSF9 gene was also upregulated at exposure to high doses of e.g. Styrene 7,8-oxide and Hydroquinone. In contrast, TNFRSF10A was downregulated with increasing Epichlorohydrin dose.

## Discussion

The current study was set up to detect gene signatures and biological pathways altered by exposure to occupational and environmental carcinogens with diverse modes of action (MOA), using global gene expression analysis. S9 was included for agents requiring metabolic activation. S9 was found to have an impact on some of the same pathways as the carcinogens, making it difficult to distinguish carcinogenic-specific effects. As a consequence, gene expression data of experiments with S9 were considered independently and are given in the supplementary files. Our findings suggest that experimental exposure conditions should be considered carefully when carrying out all *in vitro* gene expression experiments to classify indirect acting carcinogens using S9 metabolic mix.

### Comparison with previous *in vitro* studies of global gene expression induced by exposure to mutagenic compounds

Our data confirm the results of Islaih et al. (2005), who found few robust changes in global gene expression induced by exposure to mutagenic compounds [36]. In contrast to Hu et al. (2004), who measured the response to cytostatic agents and found 17 genes downregulated and 26 genes upregulated in a dose-responsive manner, no common, robust, dose-responsive gene expression changes were found for all agents in our study [27]. However, with increasing concentration, increased numbers of genes were affected, indicating dose-responsiveness. Further, clear dose-dependent gene expression changes could be detected for Benzene, Hydroquinone, Benz[a]anthracene, Trichloroethylene and Epichlorohydrin. The genes identified by Hu et al. (2004) encoded products involved in (anti-)apoptosis and in pathways involved in maintaining cell survival [27]. In our study, some of the genes that were significantly altered were also involved in the regulation of (anti-)apoptosis and maintenance of cell survival (DNA repair, regulation of cell cycle). In addition, genes from pathways involved in the regulation of immune response and translation regulation were also altered in our study. These results are in agreement with studies describing gene alterations in cell cycle arrest, tumor necrosis factor-related pathways, immune and stress response and DNA repair after exposure to cigarette smoke, reactive oxygen species and other genotoxic agents [30], [36], [37].

Previous studies examining gene expression profiles induced by genotoxic agents were carried out in several cell types (human peripheral blood mononuclear cells, HepG2, L5178Y mouse lymphoma cells, TK6 cells) and show a large variation in the differential expression level of individual genes [26], [27], [30], [31], [36], [37]. The two studies previously carried out in TK6 cells assessed effects of anti-cancer drugs and showed effects on the same pathways as in our study [31], [36]. Differences in the expression levels of individual genes between the studies may partially be explained by different treatment schedules and times at which gene expression measurements were made. The statistical approach used in the present study differs from the approaches of previous studies, in which genes were selected based on fold expression changes, and could also explain some of the different outcomes compared to other studies [27], [29], [31].

In this study we could not find similar pathways among all direct or indirect- acting carcingoens or pathways, which could discriminate between direct- and indirect- acting agents. We found that specific KEGG pathways exhibit similar expression for certain chemical-dose combinations. The identified KEGG pathways are mainly involved in cell cycle control, DNA repair mechanisms, apoptosis, immune response, p53 signaling pathway and intracellular signaling pathways. Activation of DNA repair related pathways suggests that carcinogenic exposure elicits a DNA damage response. Activation of ubiquitin mediated proteolysis pathway also suggests the induction of apoptosis [38]. In general, cellular processes identified by GO analysis and KEGG pathway analysis were similar. As shown in the heatmap, upregulation of ribosomal pathways by exposure to many chemical-dose combinations was significant. This may reflect the prevention of shutdown of the translational machinery after carcinogenic exposure and an inherent defense system to restore the cellular homeostasis through activation of cellular translation.

### Effects of individual agents and comparison with previous studies

We examined the gene expression alterations induced by individual agents. Altered expression of a set of genes involved in the regulation of tumor necrosis factor receptors at all levels of exposure to several agents was striking. The gene product of *TNFRSF9* (CD137), which was upregulated with increasing dose in our study, enhances immune activity to eliminate tumors. *TNFRSF10B* (CD262), which was upregulated, and *TNFRSF10A*, which was downregulated, in our study with increasing dose, both encode receptors that can be activated by tumor necrosis factor-related apoptosis inducing ligand to transduce an apoptosis signal and induce cell apoptosis. The effects on tumor necrosis factor-related target genes and impact on both induction of and protection from apoptosis was reported previously [36].

Mitomycin C exposure modified the expression of 21 genes of which 3 were downregulated. The number of genes affected is within the range of another study [36] examining TK6 cells exposed to Mitomycin C. In contrast to that study, however, with mitomycin C exposure we did not find an effect on genes involved in the upregulation of apoptosis (e.g. MYC). Some of the pathways e.g., cell cycle, DNA repair affected by the formaldehyde exposure in our study are in agreement with another *in vivo* study performed on rat nasal epithelium exposed to Formaldehyde [39]. GO categories altered by exposure to Benzene and its metabolites, mainly Hydroquinone, were also in agreement with previous studies [40], [41]. A study of Carbontertracholoride-induced toxicity to rat liver demonstrated the induction of cellular damage followed by the activation of DNA repair genes [14]. Many of the Carbontetrachloride-induced gene expression alterations profiled in the current study were also involved in regulating the immune response, cellular toxicity, DNA damage response and apoptosis. In general, more genes were affected at high concentrations of some/several carcinogens, but also the numbers of genes playing a role in apoptosis and control of cell cycle, specifically.

### Relevance to biomarker development

In this study we also looked into the genes and biochemical pathways that represent potential biomarkers of exposure/effect of carcinogens in human cells *in vitro*. The relevance of these genes and pathways to *in vivo* biomarkers is uncertain. Peripheral blood mononuclear cells (PBMC) are easily accessible in human biomonitoring studies. A similar transcriptomic response in the PBMC of exposed individuals as in the *in vitro* studies could suggest its applicability as a biomarker. Comparing the gene expression data of Benzene and Hydroquinone in TK6 cells from the present study, with changes in the PBMC transcriptome associated with occupational benzene exposure, reveals that similar pathways (e.g. apoptosis, immune response), but not genes, are affected [42], [43]. A number of differences potentially underlie the differential effects on TK6 and human PBMC, such as the fact that TK6 cells are lymphoblastoid while PBMC comprises a number of different cell types at various stages of development; the Benzene in our *in vitro* is unmetabolized; and, Hydroquinone is only one active metabolite of Benzene. As well as differences in cell type, *in vitro* and *in vivo* responses to chemical exposure are likely influenced by many different factors. Comparison of the genes and pathways identified in the present study with effects induced *in vitro* in PBMC from normal human donors should be performed.

### Conclusion

In conclusion, we identified perturbed gene expression and pathways induced in TK6 cells by a group of genotoxic carcinogens with diverse MOAs, as well as dose-dependent changes in gene expression and pathways. Many of the pathways play roles in the survival of the cell. A number of genes significantly impacted are involved in cell division, e.g., cell cycle arrest, tumor necrosis related pathways and anti(apoptosis), halt the cell division until the accumulated mutations, and the DNA damage is effectively repaired. Future research will involve confirmation of these effects in human PBMC exposed to these and additional carcinogens *in vitro*, to determine their applicability in human biomonitoring studies. Collaborative efforts similar to ToxCast^TM^, Tox21 projects will be required to investigate the *in vitro* and *in vivo* effects of chemicals on a large scale, to develop cost-effective assays for chemical categorization, for their toxicity assessment, and to explore chemical toxicity pathways [44].

## Materials and Methods

### Cell culture

In this *in vitro* study we assessed gene expression in TK6 cells exposed to 15 carcinogens. Human TK6 cells were chosen because they express wild-type p53, grow rapidly in suspension (population doubling time of 12–14 h), and are frequently employed in standard genetic toxicology studies [36], [45], [46]. While TK6 cells are not necessarily equivalent to the target cells for carcinogenicity, evidence suggests that they can act as surrogate target cells [36], [47]. TK6 cells, purchased from the European Collection of Cell Cultures (ECACC, Wiltshire, UK), were cultured in RPMI 1640 medium containing 10% heat-inactivated horse serum, 100 U/ml penicillin, 100 μg/ml streptomycin and 2 mM l-glutamine. The cultures were maintained between 10^5^–10^6^ cells/ml, at 5% CO_2_ and 37°C. The cells, at a density of 10^6^cells/ml, were divided into 15 treatment groups and 1 control group and were exposed for 24 h to the carcinogens.

Because most compounds first pass the lung and only after absorption enter the blood and go through the liver, human liver S9 mix (1% v/v) was added to parallel cultures within each treatment and control group [37], [48]. Thus for each agent an experiment (in duplicate) was done with and without S9. Liver S9 fractions were obtained from Celsis (Neuss, Germany) and contained drug-metabolizing enzymes including the cytochromes P450, flavinmonooxygenases, and UDP glucuronyltransferases. An exogenous NADPH-regenerating system (1.3 mM NADP+, 3.3 mM glucose-6-phosphate, 0.4 U/ml glucose-6-phosphate dehydrogenase, and 3.3 mM magnesium chloride; BD Biosciences, Erembodegem, Belgium) required by liver S9 for phase I oxidation was included in the experiments.

### Chemicals, viability assays and selection of concentration

The selected agents have well-described clastogenic, genotoxic and mutagenic characteristics [49], [50]. Agents included in the current study are capable of forming adducts with DNA either directly or indirectly through the metabolic formation of reactive metabolites. Table 1 gives an overview of the agents, classification, and administered dose. In this project, we focused on carcinogens used in occupational settings. First, we selected adduct forming and cross-linking chemicals from International Agency for Research on Cancers 2007 group 1 (agents carcinogenic to humans) and 2 (agents probably/possibly carcinogenic to humans). Secondly, we classified the agents per chemical class and chose chemicals currently and frequently used in European industries and hospitals [51]. All compounds were purchased from Sigma Aldrich and dissolved and diluted in dimethylsulfoxide (DMSO).

Preliminary viability assays were performed to select three concentrations per agent, i.e. high concentration (cellular viability of 90%), medium concentration (1/10 of high concentration) and low concentration (1/100 of high concentration). We used the 3-[4,5-dimethylthiazol–2-yl]-2,5-diphenyl tetrazolium bromide (MTT) viability assay [52] and also counted the proportions of living and dead cells using a Countess^TM^ Automated Cell Counter (Invitrogen, Carlsbad, CA).

### RNA extraction, labeling, hybridization and quality control

After 24 h of treatment, cells were immediately processed for RNA isolation. RNA was extracted using Trizol® Reagent with the PureLinkTM Micro-to-Midi System® according to the manufacturer's protocol (Invitrogen, Carlsbad, CA). An on-column DNase I treatment was carried out during RNA purification (DNase I, Amplification Grade; Invitrogen, Carlsbad, CA). The quantity of RNA was measured by NanoDrop Spectrophotometry and quality (integrity) testing of RNA was done with an Agilent 2100 bioanalyzer. The RNA 260/280 ratios of all samples were above 1.84 with an RNA integrity number of at least 8.5. After isolation, RNA was stored at −80°C until analysis.

The Illumina® TotalPrep™-96 RNA Amplification Kit was used for generating biotinylated, amplified cRNA for hybridization following the manufacturer's directions. The concentration of cRNA was determined by measuring its absorbance at 260 nm using a NanoDrop Spectrophotometer and by RiboGreen fluorescence-based assay (Invitrogen). The size distribution of cRNA was evaluated by conventional denaturing agarose gel analysis. Hybridization to the Sentrix HumanRef-8 v33 Expression BeadChips (Illumina, Inc., San Diego, CA), which contain >23,000 probes/array targeting genes and known alternative splice variants from the RefSeq database release, washing and scanning were performed according to the IlluminaBeadChip manual (11286340 Rev A). Each BeadChip contained 8 microarrays allowing for the parallel processing of 8 samples for greater throughput. In order to estimate the technical variation caused by microarray sample preparation and measurement, we randomized the experiments at each level i.e., labeling, hybridization reactions and chips. We used two biological replicates per exposure condition, six biological replicates for control S9- and four biological replicates for control S9+ experiments. Quality control metrics of the microarray raw data including noise, background, % probe sets present/absent, and 3′/5′ ratios for internal control genes were satisfactory and consistent. To allow comparisons, all chips were scaled to a target intensity of 500 based on all probe sets on each chip.

### Statistical analysis

Statistical analyses were performed as described in McHale et al. (2010) [43]. Briefly, we conducted variance components analysis using a linear mixed model [53] to assess the proportion of total variation due to variation between subjects, hybridizations, labels, and chips, both before and after the normalization [quantile normalization in the affy package [54] in R [55]. For each probe, we estimated the association between exposure level and expression level using a mixed-effects model with random intercepts that accounted for clustering by treatment, hybridization, and label. The individual dose-dependent treatment effects were modeled as fixed effects. We fitted the mixed-effects model in R with the lmer function in the lme4 package [56]. The distribution (across all the probes on the microarray) of the contribution of variance from each of the sources (treatment, labeling, hybridization and chips) is plotted. We performed standard differential expression analysis on a probe-by-probe basis using a modified T-statistic based on the empirical Bayes Limma (Linear models for microarray data) approach [57], [58]. Trend analysis was performed using Limma where the doses were numerically coded as 0 (for control), 1 (for the low dose), 2 (for the medium dose) and 3 (for the high dose). After deriving the raw *p*-values on a probe by probe basis, the differentially expressed genes (probes) were selected via ranking by *p*-value and using a false discovery rate cut-off (FDR *q*-value<0.15) [59].The raw data discussed here have been deposited in the European Bioinformatics Institute ArrayExpress [60]and is publically accessible through the accession number E-TABM-1223.

### Pathway enrichment

We used a method known as structurally enhanced pathway enrichment analysis (SEPEA_NT3) [61], which incorporates the associated network information of KEGG (Kyoto Encyclopedia of Genes and Genomes) biochemical pathways [62], [63], [64]. SEPEA differs from other pathway enrichment methods in that it takes into account the network structure of the various pathways in the analyses – pathways where perturbed genes (as a result of treatment) are closely related to each other in a graph/network sense are assigned more significance. The gene-wise statistic chosen to be used by SEPEA_NT3 was the t-statistic corresponding to the treatment effect as reported by the empirical bayes Limma approach [57]. The null hypothesis tested by SEPEA_NT3 states that the distribution of the observed t-statistics among the probes corresponding to genes in a given pathway is the same as the distribution among all the probes on the microarray platform. 10^4^ permutations in the SEPEA_NT3 method were chosen in order to evaluate the significance of association of the treatment with a particular pathway.

### Gene Ontology (GO) analysis

The GO project [65] provides an ontology of defined terms representing gene product properties in the domains, cellular components, molecular functions, and biological processes. GO has a hierarchical structure that forms a directed acyclic graph in which each term has defined relationships to one or more other terms in the same domain, which can be described as parent-child relationships. Every GO term is represented by a node in this graph, and the nodes are annotated with a set of genes. We used TopGO (topology-based GO scoring; [66]) to calculate the significance of biological terms from gene expression data taking the GO structure into account [67] using the elim algorithm. The elim score is the *p*-value returned by Fisher's exact test, and a node is marked as significant if the *p*-value is smaller than a previously defined threshold [67]. Typically this threshold is set to be 0.01 divided by the number of nodes in the GO graph with at least one annotated gene. This corresponds to a Bonferroni adjustment of the *p*-values. The most highly significant nodes thus derived are denoted as key nodes.

### Generation of heatmap

Average linkage represents the criteria of assigning a new member (chemical or pathway) to a cluster if the average distance between the new member and the existing members of the cluster is small. The heat map of negative log_10_-transformed p-values representing the enrichment of the 216 pathways across the chemical treatments, was generated using hierarchical clustering with the Euclidean distance metric and average linkage to generate the hierarchical trees of pathways and chemicals using the Cluster and Tree view programs [68].

## Supporting Information

Table S1 **Impact of S9 exposure on gene expression in TK6 cells.**
(DOC)

Table S2 **Complete list of affected GO categories per chemical dose.**Functional classification of significantly altered genes by exposure to carcinogens (S9-) at low, medium and high dose into gene ontology (GO) categories were statistically performed.
(DOC)

Table S3 **Functional classification of significantly impacted genes by exposure to carcinogens (S9+) at low, medium and high dose into gene ontology (GO) categories.**
(DOC)

Table S4 **Functional classification of significantly altered genes with dose-response trend by exposure to carcinogens (S9-) into gene ontology (GO) categories.**
(DOCX)
