# Correction: Effect of Chemical Mutagens and Carcinogens on Gene Expression Profiles in Human TK6 Cells

**DOI:** 10.1371/annotation/cee00b5b-4def-4684-b02c-fdcc5eaf51e6

**Published:** 2012-07-10

**Authors:** Lode Godderis, Reuben Thomas, Alan E. Hubbard, Ali M. Tabish, Peter Hoet, Luoping Zhang, Martyn T. Smith, Hendrik Veulemans, Cliona M. McHale

In Tables 2, 3, and 4, some of the information was formatted incorrectly. Revised versions of the tables can be seen here:

Table 2: 

**Figure:**
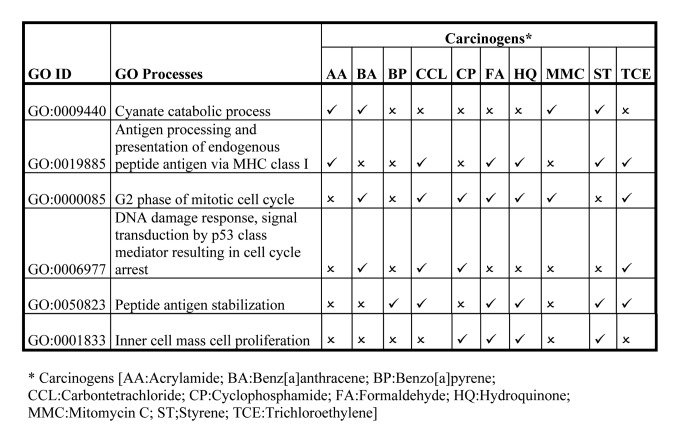


[^]

Table 3: 

**Figure:**
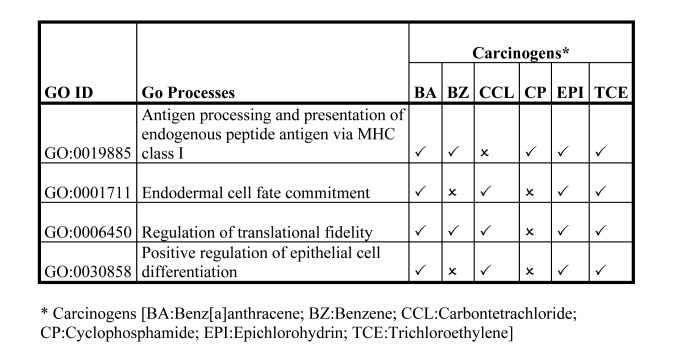


[^]

Table 4: 

**Figure:**
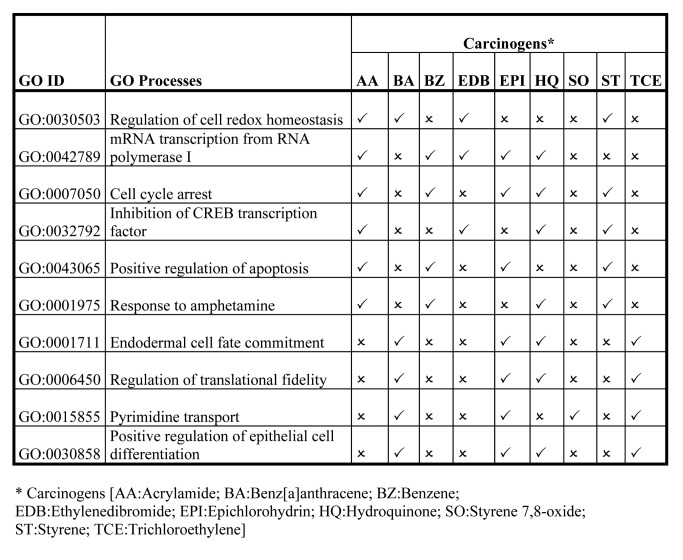


[^] 

