# Supplementary material for: Effect of Chemical Mutagens and Carcinogens on Gene Expression Profiles in Human TK6 Cells
Source: PLoS One. 2012 Jun 18;7(6):e39205. doi: 10.1371/journal.pone.0039205 (PMC3377624; doi:10.1371/journal.pone.0039205)
Supplement: Table S1 — Impact of S9 exposure on gene expression in TK6 cells. (DOC) [file pone.0039205.s001.doc]

Supplementary Table S1: Impact of S9 exposure on gene expression in TK6 cells

| **Symbol** | **Definition** |
| --- | --- |
| TRIM29 | Homo sapiens tripartite motif-containing 29 (TRIM29), transcript variant 2, mRNA. |
| MMP7 | Homo sapiens matrix metallopeptidase 7 (matrilysin, uterine) (MMP7), mRNA. |
| TNFRSF9 | Homo sapiens tumor necrosis factor receptor superfamily, member 9 (TNFRSF9), mRNA. |
| UPP1 | Homo sapiens uridine phosphorylase 1 (UPP1), transcript variant 2, mRNA. |
| TNKS1BP1 | Homo sapiens tankyrase 1 binding protein 1, 182kDa (TNKS1BP1), mRNA. |
| BIRC3 | Homo sapiens baculoviral IAP repeat-containing 3 (BIRC3), transcript variant 1, mRNA. |
| ALB | Homo sapiens albumin (ALB), mRNA. |
| WFDC5 | Homo sapiens WAP four-disulfide core domain 5 (WFDC5), mRNA. |
| ORM1 | Homo sapiens orosomucoid 1 (ORM1), mRNA. |
| GPR132 | Homo sapiens G protein-coupled receptor 132 (GPR132), mRNA. |
| SERPINA3 | Homo sapiens serpin peptidase inhibitor, clade A (alpha-1 antiproteinase, antitrypsin), member 3 (SERPINA3), mRNA. |
| GPR30 | Homo sapiens G protein-coupled estrogen receptor 1 (GPER), transcript variant 3, mRNA. |
| BIRC3 | Homo sapiens baculoviral IAP repeat-containing 3 (BIRC3), transcript variant 1, mRNA. |
| BLK | Homo sapiens B lymphoid tyrosine kinase (BLK), mRNA. |
| MYH6 | Homo sapiens myosin, heavy polypeptide 6, cardiac muscle, alpha (cardiomyopathy, hypertrophic 1) (MYH6), mRNA. |
| CCL20 | Homo sapiens chemokine (C-C motif) ligand 20 (CCL20), mRNA. |
| SMAD6 | Homo sapiens SMAD, mothers against DPP homolog 6 (Drosophila) (SMAD6), mRNA. |
| BCL2 | Homo sapiens B-cell CLL/lymphoma 2 (BCL2), nuclear gene encoding mitochondrial protein, transcript variant alpha, mRNA. |
| CCDC6 | Homo sapiens coiled-coil domain containing 6 (CCDC6), mRNA. |
| BIRC3 | Homo sapiens baculoviral IAP repeat-containing 3 (BIRC3), transcript variant 2, mRNA. |
| SPOCK2 | Homo sapiens sparc/osteonectin, cwcv and kazal-like domains proteoglycan (testican) 2 (SPOCK2), mRNA. |
| C5AR1 | Homo sapiens complement component 5a receptor 1 (C5AR1), mRNA. |
| KCNK5 | Homo sapiens potassium channel, subfamily K, member 5 (KCNK5), mRNA. |
| CFLAR | Homo sapiens CASP8 and FADD-like apoptosis regulator (CFLAR), mRNA. |
| NR5A2 | Homo sapiens nuclear receptor subfamily 5, group A, member 2 (NR5A2), transcript variant 1, mRNA. |
| APOD | Homo sapiens apolipoprotein D (APOD), mRNA. |
| PALM | Homo sapiens paralemmin (PALM), mRNA. |
| PALM | Homo sapiens paralemmin (PALM), transcript variant 1, mRNA. |
| TNIP2 | Homo sapiens TNFAIP3 interacting protein 2 (TNIP2), mRNA. |
| SSTR2 | Homo sapiens somatostatin receptor 2 (SSTR2), mRNA. |
| AQP9 | Homo sapiens aquaporin 9 (AQP9), mRNA. |
| TRIM31 | Homo sapiens tripartite motif-containing 31 (TRIM31), mRNA." |
| TEKT4 | Homo sapiens tektin 4 (TEKT4), mRNA." |
| UBD | Homo sapiens ubiquitin D (UBD), mRNA." |
| UPB1 | Homo sapiens ureidopropionase, beta (UPB1), mRNA." |
| TMEM173 | Homo sapiens transmembrane protein 173 (TMEM173), mRNA." |
| LAIR2 | Homo sapiens leukocyte-associated Ig-like receptor 2 (LAIR2), transcript variant 1, mRNA. |
| DUSP10 | Homo sapiens dual specificity phosphatase 10 (DUSP10), transcript variant 1, mRNA." |
| TAPBP | Homo sapiens TAP binding protein (tapasin) (TAPBP), transcript variant 3, mRNA." |
| LACTB | Homo sapiens lactamase, beta (LACTB), nuclear gene encoding mitochondrial protein, transcript variant 1, mRNA." |
| FKBP5 | Homo sapiens FK506 binding protein 5 (FKBP5), mRNA. |
| CD40 | Homo sapiens CD40 antigen (TNF receptor superfamily member 5) (CD40), transcript variant 1, mRNA. |
| LAIR2 | Homo sapiens leukocyte-associated immunoglobulin-like receptor 2 (LAIR2), transcript variant 2, mRNA. |
| KLF6 | Homo sapiens Kruppel-like factor 6 (KLF6), transcript variant 1, mRNA. |
| EBI3 | Homo sapiens Epstein-Barr virus induced gene 3 (EBI3), mRNA. |
| IRF7 | Homo sapiens interferon regulatory factor 7 (IRF7), transcript variant c, mRNA. |
| RASSF4 | Homo sapiens Ras association (RalGDS/AF-6) domain family 4 (RASSF4), mRNA. |
| GPR30 | Homo sapiens G protein-coupled receptor 30 (GPR30), transcript variant 1, mRNA. |
| CXCR4 | Homo sapiens chemokine (C-X-C motif) receptor 4 (CXCR4), transcript variant 1, mRNA. |
| SKP2 | Homo sapiens S-phase kinase-associated protein 2 (p45) (SKP2), transcript variant 2, mRNA. |
| LINCR | Homo sapiens likely ortholog of mouse lung-inducible Neutralized-related C3HC4 RING domain protein (LINCR), mRNA. |
| FAH | Homo sapiens fumarylacetoacetate hydrolase (fumarylacetoacetase) (FAH), mRNA. |
| C8orf46 | Homo sapiens chromosome 8 open reading frame 46 (C8orf46), mRNA. |
| TAP1 | Homo sapiens transporter 1, ATP-binding cassette, sub-family B (MDR/TAP) (TAP1), mRNA. |
| NFE2L1 | Homo sapiens nuclear factor (erythroid-derived 2)-like 1 (NFE2L1), mRNA. |
| MGC4172 | Homo sapiens short-chain dehydrogenase/reductase (MGC4172), mRNA. |
| OR51B4 | Homo sapiens olfactory receptor, family 51, subfamily B, member 4 (OR51B4), mRNA. |
| DKFZP564O0823 | Homo sapiens DKFZP564O0823 protein (DKFZP564O0823), mRNA. |
| STARD10 | Homo sapiens START domain containing 10 (STARD10), mRNA. |
| GMDS | Homo sapiens GDP-mannose 4,6-dehydratase (GMDS), mRNA. |
| BIN1 | Homo sapiens bridging integrator 1 (BIN1), transcript variant 1, mRNA. |
| MYT1 | Homo sapiens myelin transcription factor 1 (MYT1), mRNA. |
| HCST | Homo sapiens hematopoietic cell signal transducer (HCST), transcript variant 1, mRNA. |
| ICA1 | Homo sapiens islet cell autoantigen 1, 69kDa (ICA1), transcript variant 2, mRNA. |
| FCER1G | Homo sapiens Fc fragment of IgE, high affinity I, receptor for; gamma polypeptide (FCER1G), mRNA. |
| RGS1 | Homo sapiens regulator of G-protein signalling 1 (RGS1), mRNA. |
| DUSP10 | Homo sapiens dual specificity phosphatase 10 (DUSP10), transcript variant 3, mRNA. |
| C3orf54 | Homo sapiens chromosome 3 open reading frame 54 (C3orf54), mRNA. |
| CCL22 | Homo sapiens chemokine (C-C motif) ligand 22 (CCL22), mRNA. |
| KLF6 | Homo sapiens Kruppel-like factor 6 (KLF6), transcript variant 2, mRNA. |
| NRIP1 | Homo sapiens nuclear receptor interacting protein 1 (NRIP1), mRNA. |
| DFNB31 | Homo sapiens deafness, autosomal recessive 31 (DFNB31), mRNA |
| P2RX5 | Homo sapiens purinergic receptor P2X, ligand-gated ion channel, 5 (P2RX5), transcript variant 3, mRNA. |
| OPA1 | Homo sapiens optic atrophy 1 (autosomal dominant) (OPA1), nuclear gene encoding mitochondrial protein, transcript variant 6, mRNA. |
| CD80 | Homo sapiens CD80 antigen (CD28 antigen ligand 1, B7-1 antigen) (CD80), mRNA. |
| SERPINA1 | Homo sapiens serpin peptidase inhibitor, clade A (alpha-1 antiproteinase, antitrypsin), member 1 (SERPINA1), transcript variant 3, mRNA. |
| PNKD | Homo sapiens paroxysmal nonkinesiogenic dyskinesia (PNKD), transcript variant 2, mRNA. |
| TSSC1 | Homo sapiens tumor suppressing subtransferable candidate 1 (TSSC1), mRNA. |
| FSCN1 | Homo sapiens fascin homolog 1, actin-bundling protein (Strongylocentrotus purpuratus) (FSCN1), mRNA. |
| CD82 | Homo sapiens CD82 antigen (CD82), transcript variant 1, mRNA." |
| RASSF4 | Homo sapiens Ras association (RalGDS/AF-6) domain family 4 (RASSF4), transcript variant 2, mRNA. |
| ABTB2 | Homo sapiens ankyrin repeat and BTB (POZ) domain containing 2 (ABTB2), mRNA. |
| GBP4 | Homo sapiens guanylate binding protein 4 (GBP4), mRNA. |
| SRGN | Homo sapiens serglycin (SRGN), mRNA. |
| BANK1 | Homo sapiens B-cell scaffold protein with ankyrin repeats 1 (BANK1), mRNA. |
| GPM6A | Homo sapiens glycoprotein M6A (GPM6A), transcript variant 1, mRNA. |
| LSMD1 | Homo sapiens LSM domain containing 1 (LSMD1), mRNA. |
| APOM | Homo sapiens apolipoprotein M (APOM), mRNA. |
| CD82 | Homo sapiens CD82 molecule (CD82), transcript variant 2, mRNA. |
| SERPINA1 | Homo sapiens serpin peptidase inhibitor, clade A (alpha-1 antiproteinase, antitrypsin), member 1 (SERPINA1), transcript variant 2, mRNA. |
| DHRS3 | Homo sapiens dehydrogenase/reductase (SDR family) member 3 (DHRS3), mRNA. |
| PLCL2 | Homo sapiens phospholipase C-like 2 (PLCL2), mRNA. |
| WBP4 | Homo sapiens WW domain binding protein 4 (formin binding protein 21) (WBP4), mRNA. |
| IMMP2L | Homo sapiens IMP2 inner mitochondrial membrane peptidase-like (S. cerevisiae) (IMMP2L), mRNA." |
| DDX17 | Homo sapiens DEAD (Asp-Glu-Ala-Asp) box polypeptide 17 (DDX17), transcript variant 2, mRNA." |
| TPK1 | Homo sapiens thiamin pyrophosphokinase 1 (TPK1), mRNA." |
| FLJ39155 | Homo sapiens EGF-like, fibronectin type III and laminin G domains (EGFLAM), transcript variant 1, mRNA. |
| FSTL3 | Homo sapiens follistatin-like 3 (secreted glycoprotein) (FSTL3), mRNA. |
| TGM5 | Homo sapiens transglutaminase 5 (TGM5), transcript variant 1, mRNA. |
| ADAM8 | Homo sapiens ADAM metallopeptidase domain 8 (ADAM8), mRNA. |
| CNIH3 | Homo sapiens cornichon homolog 3 (Drosophila) (CNIH3), mRNA. |
| RFPL3S | Homo sapiens ret finger protein-like 3 antisense (RFPL3S) on chromosome 22. |
| CLYBL | Homo sapiens citrate lyase beta like (CLYBL), mRNA. |
| CASZ1 | Homo sapiens castor homolog 1, zinc finger (Drosophila) (CASZ1), mRNA. |
| TF | Homo sapiens transferrin (TF), mRNA. |
| VIL2 | Homo sapiens villin 2 (ezrin) (VIL2), mRNA. |
| SEC14L1 | Homo sapiens SEC14-like 1 (S. cerevisiae) (SEC14L1), transcript variant 1, mRNA. |
| DHDH | Homo sapiens dihydrodiol dehydrogenase (dimeric) (DHDH), mRNA. |
| ELMO1 | Homo sapiens engulfment and cell motility 1 (ced-12 homolog, C. elegans) (ELMO1), transcript variant 1, mRNA. |
| TSPYL2 | Homo sapiens TSPY-like 2 (TSPYL2), mRNA. |
| MTMR11 | Homo sapiens myotubularin related protein 11 (MTMR11), transcript variant 2, mRNA. |
| UBE2E2 | Homo sapiens ubiquitin-conjugating enzyme E2E 2 (UBC4/5 homolog, yeast) (UBE2E2), mRNA. |
| TNFSF4 | Homo sapiens tumor necrosis factor (ligand) superfamily, member 4 (tax-transcriptionally activated glycoprotein 1, 34kDa) (TNFSF4), mRNA |
| PCGF2 | Homo sapiens polycomb group ring finger 2 (PCGF2), mRNA. |
| CXCR4 | Homo sapiens chemokine (C-X-C motif) receptor 4 (CXCR4), transcript variant 2, mRNA. |
| PRKCZ | Homo sapiens protein kinase C, zeta (PRKCZ), transcript variant 1, mRN |
| TSC22D3 | "Homo sapiens TSC22 domain family, member 3 (TSC22D3), transcript variant 2, mRNA. |
| C16orf24 | Homo sapiens chromosome 16 open reading frame 24 (C16orf24), mRNA. |
| ACACA | Homo sapiens acetyl-Coenzyme A carboxylase alpha (ACACA), transcript variant 6, mRNA |
| CETP | Homo sapiens cholesteryl ester transfer protein, plasma (CETP), mRNA. |
| AXUD1 | Homo sapiens AXIN1 up-regulated 1 (AXUD1), mRNA. |
| C6orf125 | Homo sapiens chromosome 6 open reading frame 125 (C6orf125), mRNA. |
| EDN1 | Homo sapiens endothelin 1 (EDN1), mRNA. |
| DUSP2 | Homo sapiens dual specificity phosphatase 2 (DUSP2), mRNA. |
| SELS | Homo sapiens selenoprotein S (SELS), transcript variant 2, mRNA. |
| CABC1 | Homo sapiens chaperone, ABC1 activity of bc1 complex like (S. pombe) (CABC1), mRNA. |
| C9orf23 | Homo sapiens chromosome 9 open reading frame 23 (C9orf23), transcript variant 2, mRNA. |
| EHD1 | Homo sapiens EH-domain containing 1 (EHD1), mRNA. |
| ZC3H12A | Homo sapiens zinc finger CCCH-type containing 12A (ZC3H12A), mRNA. |
| GLIPR1 | Homo sapiens GLI pathogenesis-related 1 (glioma) (GLIPR1), mRNA. |
| SLC39A9 | Homo sapiens solute carrier family 39 (zinc transporter), member 9 (SLC39A9), mRNA. |
| SSH1 | Homo sapiens slingshot homolog 1 (Drosophila) (SSH1), mRNA. |
| RPS21 | Homo sapiens ribosomal protein S21 (RPS21), mRNA. |
| MKNK2 | Homo sapiens MAP kinase interacting serine/threonine kinase 2 (MKNK2), transcript variant 2, mRNA. |
| CD40 | Homo sapiens CD40 molecule, TNF receptor superfamily member 5 (CD40), transcript variant 2, mRNA. |
| CDC42EP4 | Homo sapiens CDC42 effector protein (Rho GTPase binding) 4 (CDC42EP4), mRNA." |
| TSC22D3 | Homo sapiens TSC22 domain family, member 3 (TSC22D3), transcript variant 2, mRNA." |
| FLJ39155 | Homo sapiens EGF-like, fibronectin type III and laminin G domains (EGFLAM), transcript variant 4, mRNA. |
| ZDHHC18 | Homo sapiens zinc finger, DHHC-type containing 18 (ZDHHC18), mRNA. |
| FLJ46321 | Homo sapiens FLJ46321 protein (FLJ46321), mRNA. |
| TRIM29 | Homo sapiens tripartite motif-containing 29 (TRIM29), transcript variant 1, mRNA. |
| HIC1 | Homo sapiens hypermethylated in cancer 1 (HIC1), mRNA. |
| FGR | Homo sapiens Gardner-Rasheed feline sarcoma viral (v-fgr) oncogene homolog (FGR), mRNA. |
| STAU2 | Homo sapiens staufen, RNA binding protein, homolog 2 (Drosophila) (STAU2), mRNA. |
| ST6GALNAC4 | Homo sapiens ST6 (alpha-N-acetyl-neuraminyl-2,3-beta-galactosyl-1, 3)-N-acetylgalactosaminide alpha-2,6-sialyltransferase 4 (ST6GALNAC4), transcript variant 3, mRNA. |
| TMEM119 | Homo sapiens transmembrane protein 119 (TMEM119), mRNA. |
| LRMP | Homo sapiens lymphoid-restricted membrane protein (LRMP), mRNA. |
| GPATC2 | Homo sapiens G patch domain containing 2 (GPATCH2), mRNA. |
| MAPKBP1 | Homo sapiens mitogen activated protein kinase binding protein 1 (MAPKBP1), mRNA. |
| SP140 | Homo sapiens SP140 nuclear body protein (SP140), transcript variant 2, mRNA. |
| OSTbeta | Homo sapiens organic solute transporter beta (OSTbeta), mRNA. |
| GLB1 | Homo sapiens galactosidase, beta 1 (GLB1), transcript variant 2, mRNA. |
| TGM5 | Homo sapiens transglutaminase 5 (TGM5), transcript variant 1, mRNA. |
| FGG | Homo sapiens fibrinogen gamma chain (FGG), transcript variant gamma-A, mRNA. |
| HRASLS2 | Homo sapiens HRAS-like suppressor 2 (HRASLS2), mRNA. |
| HAPLN3 | Homo sapiens hyaluronan and proteoglycan link protein 3 (HAPLN3), mRNA. |
| IL7R | Homo sapiens interleukin 7 receptor (IL7R), mRNA. |
| SEC14L1 | Homo sapiens SEC14-like 1 (S. cerevisiae) (SEC14L1), transcript variant 1, mRNA. |
| PIM2 | Homo sapiens pim-2 oncogene (PIM2), mRNA. |
| INPP1 | Homo sapiens inositol polyphosphate-1-phosphatase (INPP1), mRNA. |
| FBXL10 | Homo sapiens F-box and leucine-rich repeat protein 10 (FBXL10), transcript variant 1, mRNA. |
| MYH9 | Homo sapiens myosin, heavy chain 9, non-muscle (MYH9), mRNA |
| DENND3 | Homo sapiens DENN/MADD domain containing 3 (DENND3), mRNA. |
| GAMT | Homo sapiens guanidinoacetate N-methyltransferase (GAMT), transcript variant 1, mRNA. |
| FGA | Homo sapiens fibrinogen alpha chain (FGA), transcript variant alpha, mRNA. |
| TAPBP | Homo sapiens TAP binding protein (tapasin) (TAPBP), transcript variant 1, mRNA. |
| GPR55 | Homo sapiens G protein-coupled receptor 55 (GPR55), mRNA. |
| APOE | Homo sapiens apolipoprotein E (APOE), mRNA. |
| NAPSB | Homo sapiens napsin B aspartic peptidase pseudogene (NAPSB) on chromosome 19. XR_00141 |
| C6orf108 | Homo sapiens chromosome 6 open reading frame 108 (C6orf108), transcript variant 2, mRNA. |
| FAM65A | Homo sapiens family with sequence similarity 65, member A (FAM65A), mRNA. |
| SNTB1 | Homo sapiens syntrophin, beta 1 (dystrophin-associated protein A1, 59kDa, basic component 1) (SNTB1), mRNA. |
| ITGAM | Homo sapiens integrin, alpha M (complement component receptor 3, alpha; also known as CD11b (p170), macrophage antigen alpha polypeptide) (ITGAM), mRNA. |
| ICA1 | Homo sapiens islet cell autoantigen 1, 69kDa (ICA1), transcript variant 3, mRNA. |
| ACACA | Homo sapiens acetyl-Coenzyme A carboxylase alpha (ACACA), transcript variant 3, mRNA." |
| ICAM4 | Homo sapiens intercellular adhesion molecule 4, Landsteiner-Wiener blood group (ICAM4), transcript variant 1, mRNA." |
| BIN1 | Homo sapiens bridging integrator 1 (BIN1), transcript variant 6, mRNA. |
| IRF7 | Homo sapiens interferon regulatory factor 7 (IRF7), transcript variant b, mRNA. |
| CRP | Homo sapiens C-reactive protein, pentraxin-related (CRP), mRNA. |
| FARS2 | Homo sapiens phenylalanine-tRNA synthetase 2 (mitochondrial) (FARS2), nuclear gene encoding mitochondrial protein, mRNA. |
| PBX4 | Homo sapiens pre-B-cell leukemia homeobox 4 (PBX4), mRNA. |
| RALGPS1 | Homo sapiens Ral GEF with PH domain and SH3 binding motif 1 (RALGPS1), mRNA. |
| ATG7 | Homo sapiens ATG7 autophagy related 7 homolog (S. cerevisiae) (ATG7), mRNA. |
| ASB6 | Homo sapiens ankyrin repeat and SOCS box-containing 6 (ASB6), transcript variant 2, mRNA. |
| CDCA7 | Homo sapiens cell division cycle associated 7 (CDCA7), transcript variant 2, mRNA. |
| TSC22D3 | Homo sapiens TSC22 domain family, member 3 (TSC22D3), transcript variant 2, mRNA. |
| GLB1 | Homo sapiens galactosidase, beta 1 (GLB1), transcript variant 179423, mRNA. |
| ARS2 | Homo sapiens ARS2 protein (ARS2), transcript variant 1, mRNA. |
| PLA1A | Homo sapiens phospholipase A1 member A (PLA1A), mRNA. |
| PTPN3 | Homo sapiens protein tyrosine phosphatase, non-receptor type 3 (PTPN3), mRNA. |
| FLJ10661 | Homo sapiens family with sequence similarity 86, member C (FAM86C), transcript variant 2, mRNA. |
| STMN1 | Homo sapiens stathmin 1/oncoprotein 18 (STMN1), transcript variant 3, mRNA. |
| IL10RA | Homo sapiens interleukin 10 receptor, alpha (IL10RA), mRNA. |
| ACTA2 | Homo sapiens actin, alpha 2, smooth muscle, aorta (ACTA2), mRNA. |
| ZNF621 | Homo sapiens zinc finger protein 621 (ZNF621), mRNA. |
| FRMD4A | Homo sapiens FERM domain containing 4A (FRMD4A), mRNA. |
| DHRS4 | Homo sapiens dehydrogenase/reductase (SDR family) member 4 (DHRS4), mRNA. |
| NUDT3 | Homo sapiens nudix (nucleoside diphosphate linked moiety X)-type motif 3 (NUDT3), mRNA. |
| CPEB2 | Homo sapiens cytoplasmic polyadenylation element binding protein 2 (CPEB2), transcript variant A, mRNA. |
| NFKB2 | Homo sapiens nuclear factor of kappa light polypeptide gene enhancer in B-cells 2 (p49/p100) (NFKB2), mRNA |
| PMVK | Homo sapiens phosphomevalonate kinase (PMVK), mRNA. |
| FGR | Homo sapiens Gardner-Rasheed feline sarcoma viral (v-fgr) oncogene homolog (FGR), transcript variant 3, mRNA. |
| LSR | Homo sapiens lipolysis stimulated lipoprotein receptor (LSR), transcript variant 3, mRNA. |
| TCP10L | Homo sapiens t-complex 10 (mouse)-like (TCP10L), mRNA. |
| TP53I3 | Homo sapiens tumor protein p53 inducible protein 3 (TP53I3), transcript variant 2, mRNA. |
| SMAD3 | Homo sapiens SMAD family member 3 (SMAD3), mRNA. |
| LNK | Homo sapiens SH2B adaptor protein 3 (SH2B3), mRNA. |
| IRF8 | Homo sapiens interferon regulatory factor 8 (IRF8), mRNA. |
| ANKDD1A | Homo sapiens ankyrin repeat and death domain containing 1A (ANKDD1A), mRNA. |
| NHP2L1 | Homo sapiens NHP2 non-histone chromosome protein 2-like 1 (S. cerevisiae) (NHP2L1), transcript variant 2, mRNA. |
| CD86 | Homo sapiens CD86 antigen (CD28 antigen ligand 2, B7-2 antigen) (CD86), transcript variant 1, mRNA. |
| GNA15 | Homo sapiens guanine nucleotide binding protein (G protein), alpha 15 (Gq class) (GNA15), mRNA." |
| DUSP5 | Homo sapiens dual specificity phosphatase 5 (DUSP5), mRNA. |
| CDS2 | Homo sapiens CDP-diacylglycerol synthase (phosphatidate cytidylyltransferase) 2 CDS2), mRNA. |
| CAMK2D | Homo sapiens calcium/calmodulin-dependent protein kinase (CaM kinase) II delta (CAMK2D), transcript variant 2, mRNA. |
| LOC441150 | Homo sapiens similar to RIKEN cDNA 2310039H08 (LOC441150), mRNA. |
| CCDC71 | Homo sapiens coiled-coil domain containing 71 (CCDC71), mRNA. |
| C19orf12 | Homo sapiens chromosome 19 open reading frame 12 (C19orf12), transcript variant 1, mRNA. |
| HECW2 | Homo sapiens HECT, C2 and WW domain containing E3 ubiquitin protein ligase 2 (HECW2), mRNA. |
| GPR137B | Homo sapiens G protein-coupled receptor 137B (GPR137B), mRNA. |
| MULK | Homo sapiens acylglycerol kinase (AGK), mRNA. |
| CTNNBIP1 | Homo sapiens catenin, beta interacting protein 1 (CTNNBIP1), transcript variant 2, mRNA. |
| LRBA | Homo sapiens LPS-responsive vesicle trafficking, beach and anchor containing (LRBA), mRNA. |
| NECAP2 | Homo sapiens NECAP endocytosis associated 2 (NECAP2), mRNA. |
| ASB6 | Homo sapiens ankyrin repeat and SOCS box-containing 6 (ASB6), transcript variant 2, mRNA. |
| CD86 | Homo sapiens CD86 antigen (CD28 antigen ligand 2, B7-2 antigen) (CD86), transcript variant 2, mRNA. |
| CDCA7L | Homo sapiens cell division cycle associated 7-like (CDCA7L), mRNA. |
| C9orf23 | Homo sapiens chromosome 9 open reading frame 23 (C9orf23), transcript variant 1, mRNA. |
| SYT15 | Homo sapiens synaptotagmin XV (SYT15), transcript variant b, mRNA. |
| CBLB | Homo sapiens Cas-Br-M (murine) ecotropic retroviral transforming sequence b (CBLB), mRNA. |
| C8orf13 | Homo sapiens chromosome 8 open reading frame 13 (C8orf13), mRNA. |
| BCL2L1 | Homo sapiens BCL2-like 1 (BCL2L1), nuclear gene encoding mitochondrial protein, transcript variant 1, mRNA. |
| LOC57149 | Homo sapiens hypothetical protein A-211C6.1 (LOC57149), mRNA. |
| VPS26B | Homo sapiens vacuolar protein sorting 26 homolog B (yeast) (VPS26B), mRNA. |
| BMF | Homo sapiens Bcl2 modifying factor (BMF), transcript variant 4, mRNA. |
| NBPF10 | Homo sapiens neuroblastoma breakpoint family, member 10 (NBPF10), mRNA. |
| LYL1 | Homo sapiens lymphoblastic leukemia derived sequence 1 (LYL1), mRNA. |
| GADD45B | Homo sapiens growth arrest and DNA-damage-inducible, beta (GADD45B), mRNA. |
| FSD2 | Homo sapiens fibronectin type III and SPRY domain containing 2 (FSD2), mRNA. |
| SLC25A25 | Homo sapiens solute carrier family 25 (mitochondrial carrier; phosphate carrier), member 25 (SLC25A25), nuclear gene encoding mitochondrial protein, transcript variant 1, mRNA. |
| TATDN3 | PREDICTED: Homo sapiens TatD DNase domain containing 3, transcript variant 2 (TATDN3), mRNA. |
| CLYBL | Homo sapiens citrate lyase beta like (CLYBL), mRNA. |
| LOC349236 | Homo sapiens chromosome 9 open reading frame 164 (C9orf164), mRNA |
| OTP | Homo sapiens orthopedia homeobox (OTP), mRNA |
| ELP4 | Homo sapiens elongation protein 4 homolog (S. cerevisiae) (ELP4), mRNA. |
| CD47 | Homo sapiens CD47 antigen (Rh-related antigen, integrin-associated signal transducer) (CD47), transcript variant 4, mRNA. |
| FES | Homo sapiens feline sarcoma oncogene (FES), mRNA. |
| ST3GAL1 | Homo sapiens ST3 beta-galactoside alpha-2,3-sialyltransferase 1 (ST3GAL1), transcript variant 2, mRNA. |
| TDRD9 | Homo sapiens tudor domain containing 9 (TDRD9), mRNA. |
| NFKB2 | "Homo sapiens nuclear factor of kappa light polypeptide geneenhancer in B-cells 2 (p49/p100) (NFKB2), mRNA." |
| CAMK2D | Homo sapiens calcium/calmodulin-dependent protein kinase (CaM kinase) II delta (CAMK2D), transcript variant 4, mRNA. |
| KIAA0409 | Homo sapiens KIAA0409 (KIAA0409), mRNA. |
| RELB | Homo sapiens v-rel reticuloendotheliosis viral oncogene homolog B, nuclear factor of kappa light polypeptide gene enhancer in B-cells 3 (avian) (RELB), mRNA. |
| BMF | Homo sapiens Bcl2 modifying factor (BMF), transcript variant 3, mRNA. |
| NR5A2 | Homo sapiens nuclear receptor subfamily 5, group A, member 2 (NR5A2), transcript variant 2, mRNA. |
| C22orf9 | Homo sapiens chromosome 22 open reading frame 9 (C22orf9), transcript variant 1, mRNA. |
| C5 | Homo sapiens complement component 5 (C5), mRNA. |
| ARHGAP15 | "Homo sapiens Rho GTPase activating protein 15 (ARHGAP15), mRNA |
| WRN | Homo sapiens Werner syndrome (WRN), mRNA. |
| FLJ45803 | Homo sapiens FLJ45803 protein (FLJ45803), mRNA. |
| CEBPD | Homo sapiens CCAAT/enhancer binding protein (C/EBP), delta (CEBPD), mRNA. |
| HP | Homo sapiens haptoglobin (HP), mRNA. |
| LAMB3 | Homo sapiens laminin, beta 3 (LAMB3), transcript variant 1, mRNA. |
| SEPW1 | Homo sapiens selenoprotein W, 1 (SEPW1), mRNA. |
| SRC | Homo sapiens v-src sarcoma (Schmidt-Ruppin A-2) viral oncogene homolog (avian) (SRC), transcript variant 1, mRNA." |
| MITF | Homo sapiens microphthalmia-associated transcription factor (MITF), transcript variant 5, mRNA. |
| FAM107B | Homo sapiens family with sequence similarity 107, member B (FAM107B), mRNA. |
| C3orf62 | Homo sapiens chromosome 3 open reading frame 62 (C3orf62), mRNA. |
| BMP2 | Homo sapiens bone morphogenetic protein 2 (BMP2), mRNA. |
| PILRA | Homo sapiens paired immunoglobin-like type 2 receptor alpha (PILRA), transcript variant 1, mRNA. |
| C3orf9 | Homo sapiens KTEL (Lys-Tyr-Glu-Leu) containing 1 (KTELC1), mRNA. |
| FILIP1 | Homo sapiens filamin A interacting protein 1 (FILIP1), mRNA. |
| IPO11 | Homo sapiens importin 11 (IPO11), mRNA. |
| FKRP | Homo sapiens fukutin related protein (FKRP), transcript variant 1, mRNA. |
| Magmas | Homo sapiens mitochondria-associated protein involved in granulocyte-macrophage colony-stimulating factor signal transduction (Magmas), nuclear gene encoding mitochondrial protein, mRNA. |
| C1orf53 | Homo sapiens chromosome 1 open reading frame 53 (C1orf53), mRNA. |
| DOK4 | Homo sapiens docking protein 4 (DOK4), mRNA. |
| TPST2 | Homo sapiens tyrosylprotein sulfotransferase 2 (TPST2), transcript variant 1, mRNA. |
| TRERF1 | Homo sapiens transcriptional regulating factor 1 (TRERF1), transcript variant 2, mRNA. |
| FLJ10379 | Homo sapiens S1 RNA binding domain 1 (SRBD1), mRNA." |
| ERMAP | Homo sapiens erythroblast membrane-associated protein (Scianna blood group) (ERMAP), transcript variant 1, mRNA. |
| SIX4 | Homo sapiens sine oculis homeobox homolog 4 (Drosophila) (SIX4), mRNA. |
| AHNAK | Homo sapiens AHNAK nucleoprotein (AHNAK), transcript variant 1, mRNA. |
| CASP7 | Homo sapiens caspase 7, apoptosis-related cysteine peptidase (CASP7), transcript variant gamma, mRNA. |
| ESRRB | Homo sapiens estrogen-related receptor beta (ESRRB), mRNA. |
| PSCD4 | Homo sapiens pleckstrin homology, Sec7 and coiled-coil domains 4 (PSCD4), mRNA. |
| RGNEF | Homo sapiens Rho-guanine nucleotide exchange factor (RGNEF), mRNA. |
| STX4A | Homo sapiens syntaxin 4 (STX4), mRNA. |
| POU5F1P1 | Homo sapiens POU class 5 homeobox 1 pseudogene 1 (POU5F1P1) on chromosome 8. |
| DFNA5 | Homo sapiens deafness, autosomal dominant 5 (DFNA5), mRNA. |
| FGL1 | Homo sapiens fibrinogen-like 1 (FGL1), transcript variant 4, mRNA. |
| SLC2A6 | Homo sapiens solute carrier family 2 (facilitated glucose transporter), member 6 (SLC2A6), mRNA. |
| THSD1 | Homo sapiens thrombospondin, type I, domain containing 1 (THSD1), transcript variant 1, mRNA |
| DHODH | Homo sapiens dihydroorotate dehydrogenase (DHODH), nuclear gene encoding mitochondrial protein, mRNA. |
| FLJ21986 | Homo sapiens hypothetical protein FLJ21986 (FLJ21986), mRNA. |
| C10orf10 | Homo sapiens chromosome 10 open reading frame 10 (C10orf10), mRNA. |
| MRPS16 | Homo sapiens mitochondrial ribosomal protein S16 (MRPS16), nuclear gene encoding mitochondrial protein, mRNA. |
| TRIM38 | Homo sapiens tripartite motif-containing 38 (TRIM38), mRNA. |
| ANXA2 | Homo sapiens annexin A2 (ANXA2), transcript variant 1, mRNA. |
| RPS23 | Homo sapiens ribosomal protein S23 (RPS23), mRNA. |
| FLJ10081 | Homo sapiens hypothetical protein FLJ10081 (FLJ10081), mRNA. |
| DENND1A | Homo sapiens DENN/MADD domain containing 1A (DENND1A), transcript variant 2, mRNA. |
| APOL1 | Homo sapiens apolipoprotein L, 1 (APOL1), transcript variant 3, mRNA. |
| QTRT1 | Homo sapiens queuine tRNA-ribosyltransferase 1 (tRNA-guanine transglycosylase) (QTRT1), mRNA. |
| ARID4B | Homo sapiens AT rich interactive domain 4B (RBP1-like) (ARID4B), transcript variant 1, mRNA. |
| CCBL2 | Homo sapiens cysteine conjugate-beta lyase 2 (CCBL2), transcript variant 1, mRNA. |
| MRPL24 | Homo sapiens mitochondrial ribosomal protein L24 (MRPL24), nuclear gene encoding mitochondrial protein, transcript variant 2, mRNA. |
| ISG20 | Homo sapiens interferon stimulated exonuclease gene 20kDa (ISG20), mRNA. |
| YWHAZ | Homo sapiens tyrosine 3-monooxygenase/tryptophan 5-monooxygenase activation protein, zeta polypeptide (YWHAZ), transcript variant 1, mRNA. |
| MGC10992 | Homo sapiens coiled-coil domain containing 102A (CCDC102A), mRNA. |
| ALDH1L1 | Homo sapiens aldehyde dehydrogenase 1 family, member L1 (ALDH1L1), mRNA. |
| FCRL2 | Homo sapiens Fc receptor-like 2 (FCRL2), transcript variant 1, mRNA. |
| MLH1 | Homo sapiens mutL homolog 1, colon cancer, nonpolyposis type 2 (E. coli) (MLH1), mRNA. |
| DARC | Homo sapiens Duffy blood group, chemokine receptor (DARC), mRNA. |
| C20orf111 | Homo sapiens chromosome 20 open reading frame 111 (C20orf111), mRNA. |
| HPX | Homo sapiens hemopexin (HPX), mRNA. |
| ADK | Homo sapiens adenosine kinase (ADK), transcript variant ADK-short, mRNA. |
| RABL3 | Homo sapiens RAB, member of RAS oncogene family-like 3 (RABL3), mRNA." |
| ST6GALNAC4 | Homo sapiens ST6 (alpha-N-acetyl-neuraminyl-2,3-beta-galactosyl-1, 3)-N-acetylgalactosaminide alpha-2,6-sialyltransferase 4 (ST6GALNAC4), transcript variant 1, mRNA." |
| HAK | Homo sapiens heart alpha-kinase (HAK), mRNA." |
| REPS2 | PREDICTED: Homo sapiens RALBP1 associated Eps domain containing 2 (REPS2), mRNA." |
| ARHGAP4 | Homo sapiens Rho GTPase activating protein 4 (ARHGAP4), mRNA." |
| HDDC3 | Homo sapiens HD domain containing 3 (HDDC3), mRNA." |
| SLAMF1 | Homo sapiens signaling lymphocytic activation molecule family member 1 (SLAMF1), mRNA." |
| IL27RA | Homo sapiens interleukin 27 receptor, alpha (IL27RA), mRNA." |
| BCCIP | Homo sapiens BRCA2 and CDKN1A interacting protein (BCCIP), transcript variant B, mRNA." |
| ADK | Homo sapiens adenosine kinase (ADK), transcript variant ADK-long, mRNA." |
| MKNK2 | Homo sapiens MAP kinase interacting serine/threonine kinase 2 (MKNK2), transcript variant 1, mRNA. |
| POU2AF1 | Homo sapiens POU domain, class 2, associating factor 1 (POU2AF1), mRNA. |
| PKN1 | Homo sapiens protein kinase N1 (PKN1), transcript variant 1, mRNA. |
| APRT | Homo sapiens adenine phosphoribosyltransferase (APRT), transcript variant 2, mRNA. |
| ATP5G2 | Homo sapiens ATP synthase, H+ transporting, mitochondrial F0 complex, subunit c (subunit 9), isoform 2 (ATP5G2), nuclear gene encoding mitochondrial protein, transcript variant 2, mRNA. |
| LAMA5 | Homo sapiens laminin, alpha 5 (LAMA5), mRNA. |
| KLHDC4 | Homo sapiens kelch domain containing 4 (KLHDC4), mRNA. |
| PXMP3 | Homo sapiens peroxisomal membrane protein 3, 35kDa (Zellweger syndrome) (PXMP3), mRNA. |
| TSPAN4 | Homo sapiens tetraspanin 4 (TSPAN4), transcript variant 3, mRNA. |
| THEDC1 | Homo sapiens oleoyl-ACP hydrolase (OLAH), transcript variant 1, mRNA. |
| TICAM2 | Homo sapiens toll-like receptor adaptor molecule 2 (TICAM2), mRNA." |
| WHDC1 | "PREDICTED: Homo sapiens WAS protein homology region 2 domain containing 1, transcript variant 1 (WHDC1), mRNA." |
| CRADD | Homo sapiens CASP2 and RIPK1 domain containing adaptor with death domain (CRADD), mRNA. |
| PRMT2 | Homo sapiens protein arginine methyltransferase 2 (PRMT2), transcript variant 1, mRNA. |
| JUNB | Homo sapiens jun B proto-oncogene (JUNB), mRNA. |
| GLS | Homo sapiens glutaminase (GLS), mRNA. |
| C17orf65 | Homo sapiens chromosome 17 open reading frame 65 (C17orf65), mRNA. |
| SNN | Homo sapiens stannin (SNN), mRNA. |
| ZDHHC6 | Homo sapiens zinc finger, DHHC-type containing 6 (ZDHHC6), mRNA. |
| SLC6A4 | Homo sapiens solute carrier family 6 (neurotransmitter transporter, serotonin), member 4 (SLC6A4), mRNA. |
| IER3 | Homo sapiens immediate early response 3 (IER3), transcript variant long, mRNA. |
| CDK7 | Homo sapiens cyclin-dependent kinase 7 (MO15 homolog, Xenopus laevis, cdk-activating kinase) (CDK7), mRNA. |
| DAK | Homo sapiens dihydroxyacetone kinase 2 homolog (yeast) (DAK), mRNA. |
| C18orf37 | Homo sapiens chromosome 18 open reading frame 37 (C18orf37), mRNA. |
| GMIP | Homo sapiens GEM interacting protein (GMIP), mRNA. |
| DMBT1 | Homo sapiens deleted in malignant brain tumors 1 (DMBT1), transcript variant 3, mRNA. |
| MS4A8B | Homo sapiens membrane-spanning 4-domains, subfamily A, member 8B (MS4A8B), mRNA. |
| NFKB1 | Homo sapiens nuclear factor of kappa light polypeptide gene enhancer in B-cells 1 (p105) (NFKB1), mRNA. |
| PTPRE | "Homo sapiens protein tyrosine phosphatase, receptor type, E (PTPRE), transcript variant 2, mRNA. |
| SCMH1 | Homo sapiens sex comb on midleg homolog 1 (Drosophila) (SCMH1), transcript variant 1, mRNA." |
| GEM | Homo sapiens GTP binding protein overexpressed in skeletal muscle (GEM), transcript variant 2, mRNA. |
| BRD3 | Homo sapiens bromodomain containing 3 (BRD3), mRNA. |
| TSPYL2 | Homo sapiens TSPY-like 2 (TSPYL2), mRNA. |
| SAA1 | Homo sapiens serum amyloid A1 (SAA1), transcript variant 2, mRNA." |
| FAM125A | Homo sapiens family with sequence similarity 125, member A (FAM125A), mRNA." |
| BTG1 | Homo sapiens B-cell translocation gene 1, anti-proliferative (BTG1), mRNA." |
| C9orf142 | Homo sapiens chromosome 9 open reading frame 142 (C9orf142), mRNA. |
| LMNA | Homo sapiens lamin A/C (LMNA), transcript variant 2, mRNA. |
| AKAP14 | Homo sapiens A kinase (PRKA) anchor protein 14 (AKAP14), transcript variant 3, mRNA. |
| DCPS | Homo sapiens decapping enzyme, scavenger (DCPS), mRNA. |
| FLJ22222 | Homo sapiens hypothetical protein FLJ22222 (FLJ22222), transcript variant 1, mRNA. |
| C1orf19 | Homo sapiens chromosome 1 open reading frame 19 (C1orf19), mRNA. |
| DHRS4L2 | Homo sapiens dehydrogenase/reductase (SDR family) member 4 like 2 (DHRS4L2), mRNA. |
| LRRC20 | Homo sapiens leucine rich repeat containing 20 (LRRC20), transcript variant 1, mRNA. |
| RPS6KB2 | Homo sapiens ribosomal protein S6 kinase, 70kDa, polypeptide 2 (RPS6KB2), mRNA. |
| SLC45A3 | Homo sapiens solute carrier family 45, member 3 (SLC45A3), mRNA. |
| KHK | Homo sapiens ketohexokinase (fructokinase) (KHK), transcript variant a, mRNA. |
| ATP5I | Homo sapiens ATP synthase, H+ transporting, mitochondrial F0 complex, subunit E (ATP5I), nuclear gene encoding mitochondrial protein, mRNA. |
| PSD3 | Homo sapiens pleckstrin and Sec7 domain containing 3 (PSD3), transcript variant 2, mRNA. |
| CASZ1 | Homo sapiens castor zinc finger 1 (CASZ1), transcript variant 1, mRNA. |
| FLJ14466 | Homo sapiens transmembrane protein 142A (TMEM142A), mRNA. |
| PLEC1 | Homo sapiens plectin 1, intermediate filament binding proein 500kDa (PLEC1), transcript variant 11, mRNA. |
| MGC3196 | Homo sapiens hypothetical protein MGC3196 (MGC3196), mRNA. |
| BRE | Homo sapiens brain and reproductive organ-expressed (TNFRSF1A modulator) (BRE), transcript variant 3, mRNA. |
| C4orf14 | Homo sapiens chromosome 4 open reading frames 14 (C4orf14), mRNA. |
| EXOSC6 | Homo sapiens exosome component 6 (EXOSC6), mRNA. |
| NUDT6 | Homo sapiens nudix (nucleoside diphosphate linked moiety X)-type motif 6 (NUDT6), transcript variant 2, mRNA. |
| GRINA | Homo sapiens glutamate receptor, ionotropic, N-methyl D-asparate-associated protein 1 (glutamate binding) (GRINA), transcript variant 1, mRNA. |
| ZBTB10 | Homo sapiens zinc finger and BTB domain containing 10 (ZBTB10), mRNA. |
| PLEKHC1 | Homo sapiens pleckstrin homology domain containing, family C (with FERM domain) member 1 (PLEKHC1), mRNA. |
| SPATA13 | Homo sapiens spermatogenesis associated 13 (SPATA13), mRNA. |
| AKR1C2 | Homo sapiens aldo-keto reductase family 1, member C2 (dihydrodiol dehydrogenase 2; bile acid binding protein; 3-alpha hydroxysteroid dehydrogenase, type III) (AKR1C2), transcript variant 1, mRNA. |
| FAM113B | Homo sapiens family with sequence similarity 113, member B (FAM113B), mRNA. |
| PRMT2 | Homo sapiens protein arginine methyltransferase 2 (PRMT2), transcript variant 2, mRNA. |
| CRISPLD2 | Homo sapiens cysteine-rich secretory protein LCCL domain containing 2 (CRISPLD2), mRNA. |
| MARCKSL1 | Homo sapiens MARCKS-like 1 (MARCKSL1), mRNA. |
| KIAA0746 | Homo sapiens KIAA0746 protein (KIAA0746), mRNA. |
| ZDHHC14 | Homo sapiens zinc finger, DHHC-type containing 14 (ZDHHC14), transcript variant 1, mRNA. |
| AUTS2 | Homo sapiens autism susceptibility candidate 2 (AUTS2), mRNA. |
| CCND3 | "Homo sapiens cyclin D3 (CCND3), mRNA. |
| B3GNTL1 | "Homo sapiens UDP-GlcNAc:betaGal beta-1,3-N-acetylglucosaminyltransferase-like 1 (B3GNTL1), mRNA. |
| REPS2 | "Homo sapiens RALBP1 associated Eps domain containing 2 (REPS2), transcript variant 2, mRNA. |
| TLN2 | "Homo sapiens talin 2 (TLN2), mRNA. |
| PPP3R1 | "Homo sapiens protein phosphatase 3 (formerly 2B), regulatory subunit B, alpha isoform (PPP3R1), mRNA. |
| PHCA | Homo sapiens phytoceramidase, alkaline (PHCA), mRNA. |
| NSUN4 | Homo sapiens NOL1/NOP2/Sun domain family, member 4 (NSUN4), mRNA. |
| ISG15 | Homo sapiens ISG15 ubiquitin-like modifier (ISG15), mRNA. |
| TEGT | Homo sapiens testis enhanced gene transcript (BAX inhibitor 1) (TEGT), mRNA. |
| CYBRD1 | Homo sapiens cytochrome b reductase 1 (CYBRD1), mRNA. |
| SYNGR1 | Homo sapiens synaptogyrin 1 (SYNGR1), transcript variant 1b, mRNA. |
| PIP5K1C | Homo sapiens phosphatidylinositol-4-phosphate 5-kinase, type I, gamma (PIP5K1C), mRNA |
| C21orf127 | Homo sapiens chromosome 21 open reading frame 127 (C21orf127), transcript variant 1, mRNA. |
| RBM12 | Homo sapiens RNA binding motif protein 12 (RBM12), transcript variant 1, mRNA. |
| C1orf186 | Homo sapiens chromosome 1 open reading frame 186 (C1orf186), mRNA. |
| CYB561D2 | Homo sapiens cytochrome b-561 domain containing 2 (CYB561D2), mRNA. |
| BLR1 | Homo sapiens Burkitt lymphoma receptor 1, GTP binding protein (chemokine (C-X-C motif) receptor 5) (BLR1), transcript variant 2, mRNA. |
| PLOD3 | Homo sapiens procollagen-lysine, 2-oxoglutarate 5-dioxygenase 3 (PLOD3), mRNA. |
| C1orf188 | Homo sapiens chromosome 1 open reading frame 188 (C1orf188), mRNA. |
| BDH | Homo sapiens 3-hydroxybutyrate dehydrogenase (heart, mitochondrial) (BDH), nuclear gene encoding mitochondrial protein, transcript variant 1, mRNA. |
| PRIM1 | Homo sapiens primase, polypeptide 1, 49kDa (PRIM1), mRNA. |
| TM4SF4 | Homo sapiens transmembrane 4 L six family member 4 (TM4SF4), mRNA. |
| SLC20A1 | Homo sapiens solute carrier family 20 (phosphate transporter), member 1 (SLC20A1), mRNA. |
| AK2 | Homo sapiens adenylate kinase 2 (AK2), transcript variant AK2B, mRNA. |
| IRAK2 | Homo sapiens interleukin-1 receptor-associated kinase 2 (IRAK2), mRNA. |
| BZW2 | Homo sapiens basic leucine zipper and W2 domains 2 (BZW2), mRNA. |
| LRRC51 | Homo sapiens leucine rich repeat containing 51 (LRRC51), mRNA. |
| CCR7 | Homo sapiens chemokine (C-C motif) receptor 7 (CCR7), mRNA. |
| NIBP | Homo sapiens NIK and IKK{beta} binding protein (NIBP), mRNA. |
| STAT1 | Homo sapiens signal transducer and activator of transcription 1, 91kDa (STAT1), transcript variant beta, mRNA. |
| CAP1 | Homo sapiens CAP, adenylate cyclase-associated protein 1 (yeast) (CAP1), mRNA. |
| TNFRSF6B | Homo sapiens tumor necrosis factor receptor superfamily, member 6b, decoy (TNFRSF6B), transcript variant M68C, mRNA. |
| TMEM16J | Homo sapiens transmembrane protein 16J (TMEM16J), mRNA. |
| RWDD1 | Homo sapiens RWD domain containing 1 (RWDD1), transcript variant 2, mRNA. |
| C10orf99 | Homo sapiens chromosome 10 open reading frame 99 (C10orf99), mRNA. |
| P2RY8 | Homo sapiens purinergic receptor P2Y, G-protein coupled, 8 (P2RY8), mRNA. |
| DAAM2 | Homo sapiens dishevelled associated activator of morphogenesis 2 (DAAM2), mRNA. |
| WARS2 | Homo sapiens tryptophanyl tRNA synthetase 2, mitochondrial (WARS2), nuclear gene encoding mitochondrial protein, transcript variant 2, mRNA." |
| HPS1 | Homo sapiens Hermansky-Pudlak syndrome 1 (HPS1), transcript variant 3, mRNA. |
| H2AFY | Homo sapiens H2A histone family, member Y (H2AFY), transcript variant 1, mRNA. |
| DUSP1 | Homo sapiens dual specificity phosphatase 1 (DUSP1), mRNA. |
| FBXO4 | Homo sapiens F-box protein 4 (FBXO4), transcript variant 1, mRNA. |
| SELS | Homo sapiens selenoprotein S (SELS), transcript variant 1, mRNA. |
| UBE2L3 | Homo sapiens ubiquitin-conjugating enzyme E2L 3 (UBE2L3), transcript variant 2, mRNA. |
| RBM14 | Homo sapiens RNA binding motif protein 14 (RBM14), mRNA. |
| GAMT | Homo sapiens guanidinoacetate N-methyltransferase (GAMT), transcript variant 1, mRNA. |
| CARS | Homo sapiens cysteinyl-tRNA synthetase (CARS), transcript variant 4, mRNA. |
| PEMT | Homo sapiens phosphatidylethanolamine N-methyltransferase (PEMT), nuclear gene encoding mitochondrial protein, transcript variant 3, mRNA. |
| WDR69 | Homo sapiens WD repeat domain 69 (WDR69), mRNA. |
| PLA2G4C | Homo sapiens phospholipase A2, group IVC (cytosolic, calcium-independent) (PLA2G4C), mRNA. |
| SHB | Homo sapiens Src homology 2 domain containing adaptor protein B (SHB), mRNA. |
| ABHD11 | Homo sapiens abhydrolase domain containing 11 (ABHD11), transcript variant 3, mRNA. |
| STAG3 | Homo sapiens stromal antigen 3 (STAG3), mRNA. |
| RC74 | Homo sapiens integrator complex subunit 9 (INTS9), mRNA. |
| MGC3207 | Homo sapiens hypothetical protein MGC3207 (MGC3207), transcript variant 1, mRNA. |
| CPD | Homo sapiens carboxypeptidase D (CPD), mRNA. |
| COQ2 | Homo sapiens coenzyme Q2 homolog, prenyltransferase (yeast) (COQ2), mRNA. |
| C3orf37 | "Homo sapiens chromosome 3 open reading frame 37 (C3orf37), transcript variant 2, mRNA |
| HSPC049 | Homo sapiens WD repeat domain 91 (WDR91), mRNA. |
| C6orf81 | Homo sapiens chromosome 6 open reading frame 81 (C6orf81), mRNA. |
| PILRA | Homo sapiens paired immunoglobin-like type 2 receptor alpha (PILRA), transcript variant 2, mRNA. |
| HSPC142 | Homo sapiens chromosome 19 open reading frame 62 (C19orf62), transcript variant 2, mRNA. |
| ARID5A | Homo sapiens AT rich interactive domain 5A (MRF1-like) (ARID5A), mRNA. |
| RARRES3 | Homo sapiens retinoic acid receptor responder (tazarotene induced) 3 (RARRES3), mRNA. |
| GSTK1 | Homo sapiens glutathione S-transferase kappa 1 (GSTK1), mRNA. |
| DDX17 | Homo sapiens DEAD (Asp-Glu-Ala-Asp) box polypeptide 17 (DDX17), transcript variant 1, mRNA. |
| LOC124512 | Homo sapiens hypothetical protein LOC124512 (LOC124512), mRNA. |
| NIPSNAP1 | Homo sapiens nipsnap homolog 1 (C. elegans) (NIPSNAP1), mRNA. |
| RHBDF1 | Homo sapiens rhomboid 5 homolog 1 (Drosophila) (RHBDF1), mRNA. |
| C15orf39 | Homo sapiens chromosome 15 open reading frame 39 (C15orf39), mRNA. |
| PTPRK | Homo sapiens protein tyrosine phosphatase, receptor type, K (PTPRK), mRNA. |
| MFSD3 | Homo sapiens major facilitator superfamily domain containing 3 (MFSD3), mRNA. |
| S100A4 | Homo sapiens S100 calcium binding protein A4 (S100A4), transcript variant 1, mRNA. |
| NRM | Homo sapiens nurim (nuclear envelope membrane protein) (NRM), mRNA. |
| LOC642323 | "PREDICTED: Homo sapiens similar to Rap1 GTPase-GDP dissociation stimulator 1 (SMG P21 stimulatory GDP/GTP exchange protein) (SMG GDS protein) (Exchange factor smgGDS) (LOC642323), mRNA. |
| BCL2L12 | "Homo sapiens BCL2-like 12 (proline rich) (BCL2L12), transcript variant 1, mRNA. |
| PCK2 | Homo sapiens phosphoenolpyruvate carboxykinase 2 (mitochondrial) (PCK2), nuclear gene encoding mitochondrial protein, transcript variant 1, mRNA. |
| PPM1A | Homo sapiens protein phosphatase 1A (formerly 2C), magnesium-dependent, alpha isoform (PPM1A), transcript variant 2, mRNA. |
| EXOC6 | Homo sapiens exocyst complex component 6 (EXOC6), transcript variant 2, mRNA. |
| DLST | Homo sapiens dihydrolipoamide S-succinyltransferase (E2 component of 2-oxo-glutarate complex) (DLST), mRNA. |
| NEK6 | Homo sapiens NIMA (never in mitosis gene a)-related kinase 6 (NEK6), mRNA. |
| RXRA | Homo sapiens retinoid X receptor, alpha (RXRA), mRNA. |
| MKKS | Homo sapiens McKusick-Kaufman syndrome (MKKS), transcript variant 2, mRNA. |
| SLC4A2 | Homo sapiens solute carrier family 4, anion exchanger, member 2 (erythrocyte membrane protein band 3-like 1) (SLC4A2), mRNA. |
| ATPAF2 | Homo sapiens ATP synthase mitochondrial F1 complex assembly factor 2 (ATPAF2), nuclear gene encoding mitochondrial protein, mRNA. |
| MGC33648 | "Homo sapiens chromosome 5 open reading frame 35 (C5orf35), mRNA. |
| UNC119 | "Homo sapiens unc-119 homolog (C. elegans) (UNC119), transcript variant 2, mRNA. |
| VCAM1 | "Homo sapiens vascular cell adhesion molecule 1 (VCAM1), transcript variant 1, mRNA. |
| FMO4 | "Homo sapiens flavin containing monooxygenase 4 (FMO4), mRNA. |
| URP2 | "Homo sapiens UNC-112 related protein 2 (URP2), transcript variant URP2SF, mRNA. |
| PCGF1 | Homo sapiens polycomb group ring finger 1 (PCGF1), mRNA. |
| GLYCTK | Homo sapiens glycerate kinase (GLYCTK), mRNA. |
| MAP3K8 | Homo sapiens mitogen-activated protein kinase kinase kinase 8 (MAP3K8), mRNA. |
| NSFL1C | Homo sapiens NSFL1 (p97) cofactor (p47) (NSFL1C), transcript variant 1, mRNA. |
| DNAJC18 | Homo sapiens DnaJ (Hsp40) homolog, subfamily C, member 18 (DNAJC18), mRNA. |
| CSF2 | Homo sapiens colony stimulating factor 2 (granulocyte-macrophage) (CSF2), mRNA. |
| KNS2 | Homo sapiens kinesin 2 (KNS2), transcript variant 1, mRNA. |
| FAM89B | Homo sapiens family with sequence similarity 89, member B (FAM89B), mRNA. |
| PIGF | Homo sapiens phosphatidylinositol glycan, class F (PIGF), transcript variant 2, mRNA |
| C9orf123 | Homo sapiens chromosome 9 open reading frame 123 (C9orf123), mRNA. |
| IAH1 | Homo sapiens isoamyl acetate-hydrolyzing esterase 1 homolog (S. cerevisiae) (IAH1), mRNA. |
| IL1R2 | Homo sapiens interleukin 1 receptor, type II (IL1R2), transcript variant 2, mRNA. |
| S100A9 | Homo sapiens S100 calcium binding protein A9 (calgranulin B) (S100A9), mRNA. |
| AKAP7 | Homo sapiens A kinase (PRKA) anchor protein 7 (AKAP7), transcript variant gamma, mRNA. |
| CEP192 | Homo sapiens centrosomal protein 192kDa (CEP192), mRNA. |
| IFIT3 | Homo sapiens interferon-induced protein with tetratricopeptide repeats 3 (IFIT3), mRNA. |
| PARP14 | Homo sapiens poly (ADP-ribose) polymerase family, member 14 (PARP14), mRNA. |
| TRIM31 | Homo sapiens tripartite motif-containing 31 (TRIM31), transcript variant 2, mRNA. |
| MAPK10 | Homo sapiens mitogen-activated protein kinase 10 (MAPK10), transcript variant 3, mRNA." |
| ARID3A | Homo sapiens AT rich interactive domain 3A (BRIGHT- like) (ARID3A), mRNA. |
| KTN1 | Homo sapiens kinectin 1 (kinesin receptor) (KTN1), mRNA. |
| MTCH2 | Homo sapiens mitochondrial carrier homolog 2 (C. elegans) (MTCH2), nuclear gene encoding mitochondrial protein, mRNA. |
| APEH | Homo sapiens N-acylaminoacyl-peptide hydrolase (APEH), mRNA. |
| DENND1A | Homo sapiens DENN/MADD domain containing 1A (DENND1A), transcript variant 2, mRNA. |
| WDR61 | Homo sapiens WD repeat domain 61 (WDR61), mRNA. |
| RPS7 | Homo sapiens ribosomal protein S7 (RPS7), mRNA. |
| ADARB1 | Homo sapiens adenosine deaminase, RNA-specific, B1 (RED1 homolog rat) (ADARB1), transcript variant 4, mRNA. |
| JUN | Homo sapiens jun oncogene (JUN), mRNA. |
| FASTK | Homo sapiens Fas-activated serine/threonine kinase (FASTK), transcript variant 1, mRNA. |
| DHODH | Homo sapiens dihydroorotate dehydrogenase (DHODH), nuclear gene encoding mitochondrial protein, transcript variant 2, mRNA. |
| LOC150383 | Homo sapiens similar to RIKEN cDNA 2210021J22 (LOC150383), transcript variant 2, mRNA. |
| ALDH4A1 | Homo sapiens aldehyde dehydrogenase 4 family, member A1 (ALDH4A1), nuclear gene encoding mitochondrial protein, transcript variant P5CDhS, mRNA. |
| SLC44A1 | Homo sapiens solute carrier family 44, member 1 (SLC44A1), transcript variant 1, mRNA. |
| HIVEP1 | Homo sapiens human immunodeficiency virus type I enhancer binding protein 1 (HIVEP1), mRNA. |
| GRN | Homo sapiens granulin (GRN), mRNA." |
| C10orf12 | Homo sapiens chromosome 10 open reading frame 12 (C10orf12), mRNA. |
| OTOA | Homo sapiens otoancorin (OTOA), transcript variant 1, mRNA. |
| LOC90355 | Homo sapiens chromosome 5 open reading frame 30 (C5orf30), mRNA. |
| MSL3L1 | Homo sapiens male-specific lethal 3-like 1 (Drosophila) (MSL3L1), transcript variant 2, mRNA. |
| EPSTI1 | Homo sapiens epithelial stromal interaction 1 (breast) (EPSTI1), transcript ariant 2, mRNA. |
| COG6 | Homo sapiens component of oligomeric golgi complex 6 (COG6), mRNA. |
| CDGAP | Homo sapiens Cdc42 GTPase-activating protein (CDGAP), mRNA. |
| CDC42EP3 | Homo sapiens CDC42 effector protein (Rho GTPase binding) 3 (CDC42EP3), mRNA. |
| MGC13170 | Homo sapiens chromosome 19 open reading frame 48 (C19orf48), mRNA. |
| C3orf17 | Homo sapiens chromosome 3 open reading frame 17 (C3orf17), transcript variant 1, mRNA. |
| ZNF17 | Homo sapiens zinc finger protein 17 (HPF3, KOX 10) (ZNF17), mRNA. |
| IMP4 | Homo sapiens IMP4, U3 small nucleolar ribonucleoprotein, homolog (yeast) (IMP4), mRNA. |
| FLJ20125 | Homo sapiens hypothetical protein FLJ20125 (FLJ20125), mRNA. |
| HIGD2A | Homo sapiens HIG1 domain family, member 2A (HIGD2A), mRNA. |
| HERPUD1 | Homo sapiens homocysteine-inducible, endoplasmic reticulum stress-inducible, ubiquitin-like domain member 1 (HERPUD1), transcript variant 3, mRNA. |
| DNAJC5 | Homo sapiens DnaJ (Hsp40) homolog, subfamily C, member 5 (DNAJC5), mRNA. |
| APRT | Homo sapiens adenine phosphoribosyltransferase (APRT), transcript variant 1, mRNA. |
| PILRA | Homo sapiens paired immunoglobin-like type 2 receptor alpha (PILRA), transcript variant 2, mRNA. |
| BCL10 | Homo sapiens B-cell CLL/lymphoma 10 (BCL10), mRNA. |
| CHCHD5 | Homo sapiens coiled-coil-helix-coiled-coil-helix domain containing 5 (CHCHD5), mRNA. |
| TPMT | Homo sapiens thiopurine S-methyltransferase (TPMT), mRNA. |
| TMLHE | Homo sapiens trimethyllysine hydroxylase, epsilon (TMLHE), mRNA. |
| TRIM41 | Homo sapiens tripartite motif-containing 41 (TRIM41), transcript variant 1, mRNA. |
| C21orf70 | Homo sapiens chromosome 21 open reading frame 70 (C21orf70), mRNA. |
| FAM108A2 | Homo sapiens family with sequence similarity 108, member A3 (FAM108A3), mRNA. |
| ATP1A1 | Homo sapiens ATPase, Na+/K+ transporting, alpha 1 polypeptide (ATP1A1), transcript variant 1, mRNA. |
| ZFP36L2 | Homo sapiens zinc finger protein 36, C3H type-like 2 (ZFP36L2), mRNA. |
| DNAJA1 | Homo sapiens DnaJ (Hsp40) homolog, subfamily A, member 1 (DNAJA1), mRNA. |
| NBPF20 | Homo sapiens neuroblastoma breakpoint family, member 20 (NBPF20), mRNA. |
| ERN1 | Homo sapiens endoplasmic reticulum to nucleus signalling 1 (ERN1), transcript variant 2, mRNA. |
| PCNA | Homo sapiens proliferating cell nuclear antigen (PCNA), transcript variant 2, mRNA. |
| CHMP2A | Homo sapiens chromatin modifying protein 2A (CHMP2A), transcript variant 1, mRNA. |
| PDCD2 | Homo sapiens programmed cell death 2 (PDCD2), transcript variant 2, mRNA. |
| BRE | Homo sapiens brain and reproductive organ-expressed (TNFRSF1A modulator) (BRE), transcript variant 3, mRNA. |
| PHB2 | Homo sapiens prohibitin 2 (PHB2), mRNA. |
| HIST2H2AA3 | Homo sapiens histone cluster 2, H2aa3 (HIST2H2AA3), mRNA. |
| BCL3 | Homo sapiens B-cell CLL/lymphoma 3 (BCL3), mRNA. |
| CD83 | Homo sapiens CD83 antigen (activated B lymphocytes, immunoglobulin superfamily) (CD83), mRNA. |
| RHOQ | Homo sapiens ras homolog gene family, member Q (RHOQ), mRNA. |
| HARS2 | Homo sapiens histidyl-tRNA synthetase 2 (HARS2), mRNA. |
| SPG7 | Homo sapiens spastic paraplegia 7 (pure and complicated autosomal recessive) (SPG7), nuclear gene encoding mitochondrial protein, transcript variant 2, mRNA. |
| LRP16 | Homo sapiens MACRO domain containing 1 (MACROD1), mRNA. |
| NLRP3 | Homo sapiens NLR family, pyrin domain containing 3 (NLRP3), transcript variant 1, mRNA. |
| GSS | Homo sapiens glutathione synthetase (GSS), mRNA. |
| ASB9 | Homo sapiens ankyrin repeat and SOCS box-containing 9 (ASB9), transcript variant 2, mRNA. |
| PTPN22 | Homo sapiens protein tyrosine phosphatase, non-receptor type 22 (lymphoid) (PTPN22), transcript variant 2, mRNA. |
| EIF5 | Homo sapiens eukaryotic translation initiation factor 5 (EIF5), transcript variant 1, mRNA. |
| TNFRSF1B | Homo sapiens tumor necrosis factor receptor superfamily, member 1B (TNFRSF1B), mRNA. |
| PTGES2 | Homo sapiens prostaglandin E synthase 2 (PTGES2), transcript variant 2, mRNA. |
| PPP3CB | Homo sapiens protein phosphatase 3 (formerly 2B), catalytic subunit, beta isoform (PPP3CB), mRNA. |
| SRRM1 | Homo sapiens serine/arginine repetitive matrix 1 (SRRM1), mRNA. |
| C6orf75 | Homo sapiens chromosome 6 open reading frame 75 (C6orf75), transcript variant 1, mRNA. |
| ESR2 | Homo sapiens estrogen receptor 2 (ER beta) (ESR2), transcript variant b, mRNA. |
| TNFAIP3 | Homo sapiens tumor necrosis factor, alpha-induced protein 3 (TNFAIP3), mRNA. |
| CD79B | Homo sapiens CD79B antigen (immunoglobulin-associated beta) (CD79B), transcript variant 2, mRNA. |
| SLC38A1 | Homo sapiens solute carrier family 38, member 1 (SLC38A1), mRNA. |
| C21orf6 | Homo sapiens RWD domain containing 2B (RWDD2B), mRNA. |
| RPS24 | Homo sapiens ribosomal protein S24 (RPS24), transcript variant 2, mRNA. |
| EXTL2 | Homo sapiens exostoses (multiple)-like 2 (EXTL2), transcript variant 2, mRNA. |
| CABLES1 | Homo sapiens Cdk5 and Abl enzyme substrate 1 (CABLES1), transcript variant 1, mRNA. |
| NPL | Homo sapiens N-acetylneuraminate pyruvate lyase (dihydrodipicolinate synthase) (NPL), mRNA. |
| GCSH | Homo sapiens glycine cleavage system protein H (aminomethyl carrier) (GCSH), mRNA. |
| EPDR1 | Homo sapiens ependymin related protein 1 (zebrafish) (EPDR1), mRNA. |
| THADA | Homo sapiens thyroid adenoma associated (THADA), transcript variant 2, mRNA. |
| POGZ | Homo sapiens pogo transposable element with ZNF domain (POGZ), transcript variant 3, mRNA. |
| PPP1R15A | Homo sapiens protein phosphatase 1, regulatory (inhibitor) subunit 15A (PPP1R15A), mRNA. |
| APOA1BP | Homo sapiens apolipoprotein A-I binding protein (APOA1BP), mRNA. |
| TMEM126B | Homo sapiens transmembrane protein 126B (TMEM126B), mRNA. |
| NCF4 | Homo sapiens neutrophil cytosolic factor 4, 40kDa (NCF4), transcript variant 1, mRNA. |
| DPM3 | Homo sapiens dolichyl-phosphate mannosyltransferase polypeptide 3 (DPM3), transcript variant 1, mRNA. |
| KATNAL1 | Homo sapiens katanin p60 subunit A-like 1 (KATNAL1), transcript variant 1, mRNA. |
| COPS7A | Homo sapiens COP9 constitutive photomorphogenic homolog subunit 7A (Arabidopsis) (COPS7A), mRNA. |
| PNKD | Homo sapiens paroxysmal nonkinesiogenic dyskinesia (PNKD), transcript variant 1, mRNA. |
| PERLD1 | Homo sapiens per1-like domain containing 1 (PERLD1), mRNA. |
| NDUFA10 | Homo sapiens NADH dehydrogenase (ubiquinone) 1 alpha subcomplex, 10, 42kDa (NDUFA10), nuclear gene encoding mitochondrial protein, mRNA |
| BSG | Homo sapiens basigin (Ok blood group) (BSG), transcript variant 2, mRNA. |
| MLLT6 | Homo sapiens myeloid/lymphoid or mixed-lineage leukemia (trithorax homolog, Drosophila); translocated to, 6 (MLLT6), mRNA |
| LIN7A | Homo sapiens lin-7 homolog A (C. elegans) (LIN7A), mRNA. |
| CENTG3 | Homo sapiens centaurin, gamma 3 (CENTG3), transcript variant 2, mRNA. |
| RPL41 | Homo sapiens ribosomal protein L41 (RPL41), transcript variant 1, mRNA. |
| CXorf9 | Homo sapiens chromosome X open reading frame 9 (CXorf9), mRNA. |
| TP53INP2 | Homo sapiens tumor protein p53 inducible nuclear protein 2 (TP53INP2), mRNA. |
| HOP | Homo sapiens homeodomain-only protein (HOP), transcript variant 3, mRNA. |
| SCAND1 | Homo sapiens SCAN domain containing 1 (SCAND1), transcript variant 2, mRNA. |
| CNTFR | Homo sapiens ciliary neurotrophic factor receptor (CNTFR), transcript variant 1, mRNA. |
| MYL5 | Homo sapiens myosin, light chain 5, regulatory (MYL5), mRNA. |
| TSC22D3 | Homo sapiens TSC22 domain family, member 3 (TSC22D3), transcript variant 1, mRNA. |
| LOC124512 | PREDICTED: Homo sapiens hypothetical protein LOC124512 (LOC124512), mRNA. |
| CORO7 | Homo sapiens coronin 7 (CORO7), mRNA. |
| TMEM43 | Homo sapiens transmembrane protein 43 (TMEM43), mRNA. |
| SDC1 | Homo sapiens syndecan 1 (SDC1), transcript variant 2, mRNA. |
| KLF2 | Homo sapiens Kruppel-like factor 2 (lung) (KLF2), mRNA. |
| CLMN | Homo sapiens calmin (calponin-like, transmembrane) (CLMN), mRNA. |
| ATP6V1D | Homo sapiens ATPase, H+ transporting, lysosomal 34kDa, V1 subunit D (ATP6V1D), mRNA. |
| GC | Homo sapiens group-specific component (vitamin D binding protein) (GC), mRNA." |
| TROVE2 | Homo sapiens TROVE domain family, member 2 (TROVE2), transcript variant 3, mRNA. |
| NRG4 | Homo sapiens neuregulin 4 (NRG4), mRNA. |
| TM4SF11 | Homo sapiens plasma membrane proteolipid (plasmolipin) (PLLP), mRNA. |
| PFKM | Homo sapiens phosphofructokinase, muscle (PFKM), mRNA. |
| CHD1L | Homo sapiens chromodomain helicase DNA binding protein 1-like (CHD1L), mRNA. |
| WNT10B | Homo sapiens wingless-type MMTV integration site family, member 10B (WNT10B), mRNA. |
| SCAND1 | Homo sapiens SCAN domain containing 1 (SCAND1), transcript variant 1, mRNA. |
| HNRPL | Homo sapiens heterogeneous nuclear ribonucleoprotein L (HNRPL), transcript variant 2, mRNA. |
| B4GALT4 | Homo sapiens UDP-Gal:betaGlcNAc beta 1,4- galactosyltransferase, polypeptide 4 (B4GALT4), transcript variant 2, mRNA. |
| ATP1A1 | Homo sapiens ATPase, Na+/K+ transporting, alpha 1 polypeptide (ATP1A1), transcript variant 2, mRNA. |
| IFIH1 | Homo sapiens interferon induced with helicase C domain 1 (IFIH1), mRNA. |
| GSTZ1 | Homo sapiens glutathione transferase zeta 1 (maleylacetoacetate isomerase) (GSTZ1), transcript variant 2, mRNA. |
| ARL5B | Homo sapiens ADP-ribosylation factor-like 5B (ARL5B), mRNA. |
| ZFP91 | Homo sapiens zinc finger protein 91 homolog (mouse) (ZFP91), transcript variant 2, mRNA. |
| TMEM48 | Homo sapiens transmembrane protein 48 (TMEM48), mRNA. |
| SLC2A5 | Homo sapiens solute carrier family 2 (facilitated glucose/fructose transporter), member 5 (SLC2A5), mRNA. |
| ATP7A | Homo sapiens ATPase, Cu++ transporting, alpha polypeptide (Menkes syndrome) (ATP7A), mRNA. |
| ABCA11 | Homo sapiens ATP-binding cassette, sub-family A (ABC1), member 11 (pseudogene) (ABCA11) on chromosome 4. |
| LOC63920 | Homo sapiens transposon-derived Buster3 transposase-like (LOC63920), mRNA. |
| DPP7 | Homo sapiens dipeptidyl-peptidase 7 (DPP7), mRNA. |
| FLRT3 | Homo sapiens fibronectin leucine rich transmembrane protein 3 (FLRT3), transcript variant 1, mRNA. |
| TSPYL2 | Homo sapiens TSPY-like 2 (TSPYL2), mRNA. |
| CIITA | Homo sapiens class II, major histocompatibility complex, transactivator (CIITA), mRNA. |
| ZNRD1 | Homo sapiens zinc ribbon domain containing 1 (ZNRD1), transcript variant a, mRNA. |
| SLC35E4 | Homo sapiens solute carrier family 35, member E4 (SLC35E4), mRNA. |
| PNKD | Homo sapiens paroxysmal nonkinesiogenic dyskinesia (PNKD), transcript variant 2, mRNA. |
| GMPR | Homo sapiens guanosine monophosphate reductase (GMPR), mRNA. |
| HMBS | Homo sapiens hydroxymethylbilane synthase (HMBS), transcript variant 2, mRNA. |
| WDR22 | Homo sapiens WD repeat domain 22 (WDR22), mRNA. |
| PEX7 | Homo sapiens peroxisomal biogenesis factor 7 (PEX7), mRNA. |
| FRAG1 | Homo sapiens FGF receptor activating protein 1 (FRAG1), mRNA. |
| SEPN1 | Homo sapiens selenoprotein N, 1 (SEPN1), transcript variant 1, mRNA. |
| RCL1 | Homo sapiens RNA terminal phosphate cyclase-like 1 (RCL1), mRNA. |
| NDRG3 | Homo sapiens NDRG family member 3 (NDRG3), transcript variant 2, mRNA. |
| GPR84 | Homo sapiens G protein-coupled receptor 84 (GPR84), mRNA. |
| ADAMTSL1 | Homo sapiens ADAMTS-like 1 (ADAMTSL1), transcript variant 2, mRNA. |
| STAMBPL1 | Homo sapiens STAM binding protein-like 1 (STAMBPL1), mRNA. |
| PLOD2 | Homo sapiens procollagen-lysine, 2-oxoglutarate 5-dioxygenase 2 (PLOD2), transcript variant 1, mRNA. |
| BCAS4 | Homo sapiens breast carcinoma amplified sequence 4 (BCAS4), transcript variant 1, mRNA. |
| ERCC2 | Homo sapiens excision repair cross-complementing rodent repair deficiency, complementation group 2 (xeroderma pigmentosum D) (ERCC2), mRNA. |
| SERTAD1 | Homo sapiens SERTA domain containing 1 (SERTAD1), mRNA. |
| EZH2 | Homo sapiens enhancer of zeste homolog 2 (Drosophila) (EZH2), transcript variant 1, mRNA. |
| C10orf33 | Homo sapiens chromosome 10 open reading frame 33 (C10orf33), mRNA. |
| RNF157 | Homo sapiens ring finger protein 157 (RNF157), mRNA. |
| RAD51AP1 | Homo sapiens RAD51 associated protein 1 (RAD51AP1), mRNA. |
| RBAF600 | Homo sapiens retinoblastoma-associated factor 600 (RBAF600), mRNA. |
| G10 | Homo sapiens BUD31 homolog (S. cerevisiae) (BUD31), mRNA. |
| EPM2AIP1 | Homo sapiens EPM2A (laforin) interacting protein 1 (EPM2AIP1), mRNA. |
| PKN1 | Homo sapiens protein kinase N1 (PKN1), transcript variant 1, mRNA. |
| C19orf7 | Homo sapiens chromosome 19 open reading frame 7 (C19orf7), mRNA |
| CCDC28B | Homo sapiens coiled-coil domain containing 28B (CCDC28B), mRNA. |
| LASS4 | Homo sapiens LAG1 homolog, ceramide synthase 4 (LASS4), mRNA. |
| C20orf35 | Homo sapiens SYS1 Golgi-localized integral membrane protein homolog (S. cerevisiae) (SYS1), transcript variant 1, mRNA. |
| SAMD1 | Homo sapiens sterile alpha motif domain containing 1 (SAMD1), mRNA |
| SEMA4C | Homo sapiens sema domain, immunoglobulin domain (Ig), transmembrane domain (TM) and short cytoplasmic domain, (semaphorin) 4C (SEMA4C), mRNA. |
| VASP | Homo sapiens vasodilator-stimulated phosphoprotein (VASP), transcript variant 2, mRNA. |
| SETMAR | Homo sapiens SET domain and mariner transposase fusion gene (SETMAR), mRNA. |
| ASTN2 | Homo sapiens astrotactin 2 (ASTN2), transcript variant 1, mRNA. |
| HNRPM | Homo sapiens heterogeneous nuclear ribonucleoprotein M (HNRPM), transcript variant 2, mRNA. |
| HSPA1B | Homo sapiens heat shock 70kDa protein 1B (HSPA1B), mRNA. |
| SERPINB8 | Homo sapiens serpin peptidase inhibitor, clade B (ovalbumin), member 8 (SERPINB8), transcript variant 3, mRNA. |
| DCP2 | Homo sapiens DCP2 decapping enzyme homolog (S. cerevisiae) (DCP2), mRNA. |
| VARS | Homo sapiens valyl-tRNA synthetase (VARS), mRNA. |
| ACTA1 | Homo sapiens actin, alpha 1, skeletal muscle (ACTA1), mRNA. |
| ATF3 | Homo sapiens activating transcription factor 3 (ATF3), transcript variant 4, mRNA |
| IKBKE | Homo sapiens inhibitor of kappa light polypeptide gene enhancer in B-cells, kinase epsilon (IKBKE), mRNA. |
| APBA2BP | Homo sapiens amyloid beta (A4) precursor protein-binding, family A, member 2 binding protein (APBA2BP), transcript variant 2, mRNA." |
| ATPAF1 | Homo sapiens ATP synthase mitochondrial F1 complex assembly factor 1 (ATPAF1), nuclear gene encoding mitochondrial protein, transcript variant 2, mRNA." |
| REPS2 | Homo sapiens RALBP1 associated Eps domain containing 2 (REPS2), mRNA." |
| CSTF3 | Homo sapiens cleavage stimulation factor, 3' pre-RNA, subunit 3, 77kDa (CSTF3), transcript variant 2, mRNA. |
| HMHA1 | Homo sapiens histocompatibility (minor) HA-1 (HMHA1), mRNA. |
| HEY1 | Homo sapiens hairy/enhancer-of-split related with YRPW motif 1 (HEY1), mRNA. |
| SPAG1 | Homo sapiens sperm associated antigen 1 (SPAG1), transcript variant 1, mRNA. |
| PIGV | Homo sapiens phosphatidylinositol glycan anchor biosynthesis, class V (PIGV), mRNA. |
| POLQ | Homo sapiens polymerase (DNA directed), theta (POLQ), mRNA. |
| CNIH | Homo sapiens cornichon homolog (Drosophila) (CNIH), mRNA. |
| TEX2 | Homo sapiens testis expressed 2 (TEX2), mRNA. |
| BCL2L12 | Homo sapiens BCL2-like 12 (proline rich) (BCL2L12), transcript variant 2, mRNA. |
| MARVELD2 | Homo sapiens MARVEL domain containing 2 (MARVELD2), transcript variant 1, mRNA. |
| IL12A | Homo sapiens interleukin 12A (natural killer cell stimulatory factor 1, cytotoxic lymphocyte maturation factor 1, p35) (IL12A), mRNA. |
| ISGF3G | Homo sapiens interferon-stimulated transcription factor 3, gamma 48kDa (ISGF3G), mRNA. |
| NSFL1C | Homo sapiens NSFL1 (p97) cofactor (p47) (NSFL1C), transcript variant 3, mRNA. |
| BASP1 | Homo sapiens brain abundant, membrane attached signal protein 1 (BASP1), mRNA. |
| C17orf75 | Homo sapiens chromosome 17 open reading frame 75 (C17orf75), mRNA. |
| PP2447 | Homo sapiens TraB domain containing (TRABD), mRNA. |
| ILDR1 | Homo sapiens immunoglobulin-like domain containing receptor 1 (ILDR1), mRNA. |
| LIMA1 | Homo sapiens LIM domain and actin binding 1 (LIMA1), mRNA. |
| TADA3L | Homo sapiens transcriptional adaptor 3 (NGG1 homolog, yeast)-like (TADA3L), transcript variant 1, mRNA. |
| MRPS27 | Homo sapiens mitochondrial ribosomal protein S27 (MRPS27), nuclear gene encoding mitochondrial protein, mRNA. |
| FANCG | Homo sapiens Fanconi anemia, complementation group G (FANCG), mRNA." |
| NME1-NME2 | Homo sapiens NM23-LV (NME1-NME2), mRNA." |
| ATP9B | Homo sapiens ATPase, Class II, type 9B (ATP9B), mRNA." |
| NFAT5 | Homo sapiens nuclear factor of activated T-cells 5, tonicity-responsive (NFAT5), transcript variant 2, mRNA. |
| CGI-96 | Homo sapiens CGI-96 protein (CGI-96), mRNA. |
| ARL4 | Homo sapiens ADP-ribosylation factor-like 4 (ARL4), transcript variant 1, mRNA. |
| LIPT1 | Homo sapiens lipoyltransferase 1 (LIPT1), transcript variant 2, mRNA. |
| GDF15 | Homo sapiens growth differentiation factor 15 (GDF15), mRNA. |
| ATP1B3 | Homo sapiens ATPase, Na+/K+ transporting, beta 3 polypeptide (ATP1B3), mRNA. XM_945518 |
| SH3PX3 | Homo sapiens SH3 and PX domain containing 3 (SH3PX3), mRNA. |
| SNRPA | Homo sapiens small nuclear ribonucleoprotein polypeptide A (SNRPA), mRNA. |
| C6orf149 | Homo sapiens LYR motif containing 4 (LYRM4), mRNA. |
| ORM2 | Homo sapiens orosomucoid 2 (ORM2), mRNA |
| POFUT1 | Homo sapiens protein O-fucosyltransferase 1 (POFUT1), transcript variant 1, mRNA. |
| ERRFI1 | Homo sapiens ERBB receptor feedback inhibitor 1 (ERRFI1), mRNA. |
| ALDH4A1 | Homo sapiens aldehyde dehydrogenase 4 family, member A1 (ALDH4A1), nuclear gene encoding mitochondrial protein, transcript variant P5CDhL, mRNA. |
| BDP1 | Homo sapiens B double prime 1, subunit of RNA polymerase III transcription initiation factor IIIB (BDP1), mRNA. |
| TICAM1 | Homo sapiens toll-like receptor adaptor molecule 1 (TICAM1), transcript variant 1, mRNA |
| EYA3 | Homo sapiens eyes absent homolog 3 (Drosophila) (EYA3), transcript variant 2, mRNA. |
| GBL | Homo sapiens G protein beta subunit-like (GBL), mRNA. |
| RNF19 | Homo sapiens ring finger protein 19 (RNF19), transcript variant 1, mRNA. |
| ADFP | Homo sapiens adipose differentiation-related protein (ADFP), mRNA. |
| C21orf2 | Homo sapiens chromosome 21 open reading frame 2 (C21orf2), mRNA. |
| NPC1 | Homo sapiens Niemann-Pick disease, type C1 (NPC1), mRNA. |
| IL1R2 | Homo sapiens interleukin 1 receptor, type II (IL1R2), transcript variant 2, mRNA. |
| MRPL54 | Homo sapiens mitochondrial ribosomal protein L54 (MRPL54), nuclear gene encoding mitochondrial protein, mRNA. |
| IL4I1 | "Homo sapiens interleukin 4 induced 1 (IL4I1), transcript variant 1, mRNA. |
| ZMYND11 | Homo sapiens zinc finger, MYND domain containing 11 (ZMYND11), transcript variant 2, mRNA. |
| HMBS | Homo sapiens hydroxymethylbilane synthase (HMBS), transcript variant 1, mRNA. |
| GIT2 | Homo sapiens G protein-coupled receptor kinase interactor 2 (GIT2), transcript variant 4, mRNA. |
| LOC205251 | Homo sapiens hypothetical protein LOC205251 (LOC205251), mRNA. |
| SDF2 | Homo sapiens stromal cell-derived factor 2 (SDF2), mRNA. |
| NAG | Homo sapiens neuroblastoma-amplified protein (NAG), mRNA. |
| LACTB2 | Homo sapiens lactamase, beta 2 (LACTB2), mRNA. |
| CLN6 | Homo sapiens ceroid-lipofuscinosis, neuronal 6, late infantile, variant (CLN6), mRNA. |
| DNASE1L3 | Homo sapiens deoxyribonuclease I-like 3 (DNASE1L3), mRNA. |
| SFXN4 | Homo sapiens sideroflexin 4 (SFXN4), transcript variant 2, mRNA. |
| TOMM22 | Homo sapiens translocase of outer mitochondrial membrane 22 homolog (yeast) (TOMM22), nuclear gene encoding mitochondrial protein, mRNA. |
| KIAA1712 | Homo sapiens KIAA1712 (KIAA1712), mRNA. |
| C6orf85 | Homo sapiens chromosome 6 open reading frame 85 (C6orf85), mRNA. |
| PDE7A | Homo sapiens phosphodiesterase 7A (PDE7A), transcript variant 2, mRNA. |
| ASPSCR1 | Homo sapiens alveolar soft part sarcoma chromosome region, candidate 1 (ASPSCR1), mRNA. |
| ST3GAL3 | Homo sapiens ST3 beta-galactoside alpha-2,3-sialyltransferase 3 (ST3GAL3), transcript variant 10, mRNA. |
| HAGHL | Homo sapiens hydroxyacylglutathione hydrolase-like (HAGHL), transcript variant 2, mRNA. |
| DCTN2 | Homo sapiens dynactin 2 (p50) (DCTN2), mRNA. |
| C10orf65 | Homo sapiens chromosome 10 open reading frame 65 (C10orf65), mRNA. |
| CXorf12 | Homo sapiens chromosome X open reading frame 12 (CXorf12), mRNA. |
| MUM1 | Homo sapiens melanoma associated antigen (mutated) 1 (MUM1), mRNA. |
| HIPK2 | Homo sapiens homeodomain interacting protein kinase 2 (HIPK2), mRNA. |
| ZNFX1 | Homo sapiens zinc finger, NFX1-type containing 1 (ZNFX1), mRNA. |
| DNAJB2 | Homo sapiens DnaJ (Hsp40) homolog, subfamily B, member 2 (DNAJB2), transcript variant 2, mRNA. |
| FIBP | Homo sapiens fibroblast growth factor (acidic) intracellular binding protein (FIBP), transcript variant 1, mRNA. |
| NUDT2 | Homo sapiens nudix (nucleoside diphosphate linked moiety X)-type motif 2 (NUDT2), transcript variant 3, mRNA. |
| MMS19L | Homo sapiens MMS19-like (MET18 homolog, S. cerevisiae) (MMS19L), mRNA. |
| DPP3 | Homo sapiens dipeptidylpeptidase 3 (DPP3), transcript variant 2, mRNA. |
| KIAA0738 | Homo sapiens KIAA0738 gene product (KIAA0738), mRNA. |
| ANXA5 | Homo sapiens annexin A5 (ANXA5), mRNA. |
| RPL28 | Homo sapiens ribosomal protein L28 (RPL28), mRNA. |
| PPEF1 | Homo sapiens protein phosphatase, EF-hand calcium binding domain 1 (PPEF1), transcript variant 1, mRNA. |
| FLJ45032 | Homo sapiens similar to F40B5.2b (FLJ45032), mRNA. |
| ILVBL | Homo sapiens ilvB (bacterial acetolactate synthase)-like (ILVBL), mRNA. |
| CCNDBP1 | Homo sapiens cyclin D-type binding-protein 1 (CCNDBP1), transcript variant 2, mRNA. |
| SLC37A1 | Homo sapiens solute carrier family 37 (glycerol-3-phosphate transporter), member 1 (SLC37A1), mRNA. |
| ATP2B4 | Homo sapiens ATPase, Ca++ transporting, plasma membrane 4 (ATP2B4), transcript variant 2, mRNA. |
| OXCT1 | Homo sapiens 3-oxoacid CoA transferase 1 (OXCT1), nuclear gene encoding mitochondrial protein, mRNA. |
| HDHD1A | Homo sapiens haloacid dehalogenase-like hydrolase domain containing 1A (HDHD1A), mRNA. |
| MAPKAPK2 | Homo sapiens mitogen-activated protein kinase-activated protein kinase 2 (MAPKAPK2),transcript variant 2, mRNA. |
| DPH5 | Homo sapiens DPH5 homolog (S. cerevisiae) (DPH5), transcript variant 3, mRNA. |
| SERINC2 | Homo sapiens serine incorporator 2 (SERINC2), mRNA. |
| ZNF641 | Homo sapiens zinc finger protein 641 (ZNF641), mRNA. |
| MSRA | Homo sapiens methionine sulfoxide reductase A (MSRA), mRNA. |
| C16orf55 | Homo sapiens chromosome 16 open reading frame 55 (C16orf55), mRNA. |
| CHD9 | Homo sapiens chromodomain helicase DNA binding protein 9 (CHD9), mRNA. |
| LMBR1 | Homo sapiens limb region 1 homolog (mouse) (LMBR1), mRNA. |
| BTBD7 | Homo sapiens BTB (POZ) domain containing 7 (BTBD7), transcript variant 1, mRNA. |
| MRPS7 | Homo sapiens mitochondrial ribosomal protein S7 (MRPS7), nuclear gene encoding mitochondrial protein, mRNA. |
| ATG10 | Homo sapiens ATG10 autophagy related 10 homolog (S. cerevisiae) (ATG10), mRNA. |
| TOMM20 | Homo sapiens translocase of outer mitochondrial membrane 20 homolog (yeast) (TOMM20), mRNA. |
| LOC643853 | PREDICTED: Homo sapiens similar to F40B5.2b, transcript variant 1 (LOC643853), mRNA. |
| MFI2 | Homo sapiens antigen p97 (melanoma associated) identified by monoclonal antibodies 133.2 and 96.5 (MFI2), transcript variant 2, mRNA. |
| ELF3 | Homo sapiens E74-like factor 3 (ets domain transcription factor, epithelial-specific ) (ELF3), mRNA. |
| MGC4825 | Homo sapiens apolipoprotein O (APOO), mRNA. |
| PTGES2 | Homo sapiens prostaglandin E synthase 2 (PTGES2), transcript variant 2, mRNA. |
| C1QTNF1 | Homo sapiens C1q and tumor necrosis factor related protein 1 (C1QTNF1), mRNA. |
| TAP2 | Homo sapiens transporter 2, ATP-binding cassette, sub-family B (MDR/TAP) (TAP2), transcript variant 1, mRNA |
| DDX41 | Homo sapiens DEAD (Asp-Glu-Ala-Asp) box polypeptide 41 (DDX41), mRNA. |
| RHOF | Homo sapiens ras homolog gene family, member F (in filopodia) (RHOF), mRNA. |
| TMEM116 | Homo sapiens transmembrane protein 116 (TMEM116), mRNA. |
| CDKN2D | Homo sapiens cyclin-dependent kinase inhibitor 2D (p19, inhibits CDK4) (CDKN2D), transcript variant 1, mRNA. |
| ETFB | Homo sapiens electron-transfer-flavoprotein, beta polypeptide (ETFB), transcript variant 2, mRNA. |
| FAM79A | "Homo sapiens family with sequence similarity 79, member A (FAM79A), mRNA. |
| MRPL4 | "Homo sapiens mitochondrial ribosomal protein L4 (MRPL4), nuclear gene encoding mitochondrial protein, transcript variant 1, mRNA. |
| COL7A1 | "Homo sapiens collagen, type VII, alpha 1 (epidermolysis bullosa, dystrophic, dominant and recessive) (COL7A1), mRNA. |
| TCP10 | Homo sapiens t-complex 10 (mouse) (TCP10), mRNA. |
| NCF1 | Homo sapiens neutrophil cytosolic factor 1 (47kDa, chronic granulomatous disease, autosomal 1) (NCF1), mRNA. |
| MPP6 | Homo sapiens membrane protein, palmitoylated 6 (MAGUK p55 subfamily member 6) (MPP6), mRNA. |
| DNAJA3 | Homo sapiens DnaJ (Hsp40) homolog, subfamily A, member 3 (DNAJA3), mRNA. |
| ASAH1 | Homo sapiens N-acylsphingosine amidohydrolase (acid ceramidase) 1 (ASAH1), transcript variant 1, mRNA. |
| GLIS2 | Homo sapiens GLIS family zinc finger 2 (GLIS2), mRNA. |
| DPP7 | Homo sapiens dipeptidyl-peptidase 7 (DPP7), mRNA. |
| CPNE7 | Homo sapiens copine VII (CPNE7), transcript variant 1, mRNA. |
| SLC25A40 | Homo sapiens solute carrier family 25, member 40 (SLC25A40), mRNA. |
| KIAA1328 | "Homo sapiens KIAA1328 (KIAA1328), mRNA. |
| LNPEP | Homo sapiens leucyl/cystinyl aminopeptidase (LNPEP), transcript variant 1, mRNA. |
| CHCHD8 | Homo sapiens coiled-coil-helix-coiled-coil-helix domain containing 8 (CHCHD8), mRNA. |
| UROS | Homo sapiens uroporphyrinogen III synthase (congenital erythropoietic porphyria) (UROS), mRNA. |
| CBX7 | Homo sapiens chromobox homolog 7 (CBX7), mRNA. |
| LOC643591 | PREDICTED: Homo sapiens hypothetical protein LOC643591 (LOC643591), mRNA. |
| BCAS4 | Homo sapiens breast carcinoma amplified sequence 4 (BCAS4), transcript variant 1, mRNA. |
| RASSF2 | Homo sapiens Ras association (RalGDS/AF-6) domain family 2 (RASSF2), transcript variant 2, mRNA. |
| FLJ20558 | Homo sapiens chromosome 2 open reading frame 42 (C2orf42), mRNA. |
| CKAP1 | Homo sapiens cytoskeleton associated protein 1 (CKAP1), mRNA. |
| CEACAM1 | Homo sapiens carcinoembryonic antigen-related cell adhesion molecule 1 (biliary glycoprotein) (CEACAM1), transcript variant 2, mRNA. |
| C1orf56 | Homo sapiens chromosome 1 open reading frame 56 (C1orf56), mRNA. |
| KLC2 | Homo sapiens kinesin light chain 2 (KLC2), mRNA. |
| EPRS | Homo sapiens glutamyl-prolyl-tRNA synthetase (EPRS), mRNA. |
| FLJ22222 | PREDICTED: Homo sapiens hypothetical protein FLJ22222 (FLJ22222), mRNA. |
| CFB | Homo sapiens complement factor B (CFB), mRNA. |
| CIP29 | Homo sapiens cytokine induced protein 29 kDa (CIP29), mRNA. |
| HNRPUL2 | Homo sapiens heterogeneous nuclear ribonucleoprotein U-like 2 (HNRPUL2), mRNA. |
| SCRG1 | Homo sapiens scrapie responsive protein 1 (SCRG1), mRNA |
| PTK2B | Homo sapiens PTK2B protein tyrosine kinase 2 beta (PTK2B), transcript variant 2, mRNA. |
| ADD1 | Homo sapiens adducin 1 (alpha) (ADD1), transcript variant 1, mRNA. |
| PLA2G4B | Homo sapiens phospholipase A2, group IVB (cytosolic) (PLA2G4B), mRNA." |
| PUSL1 | Homo sapiens pseudouridylate synthase-like 1 (PUSL1), mRNA. |
| SLC6A12 | Homo sapiens solute carrier family 6 (neurotransmitter transporter, betaine/GABA), member 12 (SLC6A12), mRNA. |
| DYNLL2 | Homo sapiens dynein, light chain, LC8-type 2 (DYNLL2), mRNA. |
| IVNS1ABP | Homo sapiens influenza virus NS1A binding protein (IVNS1ABP), mRNA. |
| CCND2 | Homo sapiens cyclin D2 (CCND2), mRNA. |
| CALCOCO2 | Homo sapiens calcium binding and coiled-coil domain 2 (CALCOCO2), mRNA. |
| WDR70 | Homo sapiens WD repeat domain 70 (WDR70), mRNA. |
| CEACAM1 | Homo sapiens carcinoembryonic antigen-related cell adhesion molecule 1 (biliary glycoprotein) (CEACAM1), transcript variant 2, mRNA. |
| PNKP | Homo sapiens polynucleotide kinase 3'-phosphatase (PNKP), mRNA. |
| GLRX2 | Homo sapiens glutaredoxin 2 (GLRX2), transcript variant 2, mRNA. |
| WARS | Homo sapiens tryptophanyl-tRNA synthetase (WARS), transcript variant 1, mRNA. |
| LIG3 | Homo sapiens ligase III, DNA, ATP-dependent (LIG3), nuclear gene encoding mitochondrial protein, transcript variant alpha, mRNA. |
| FLJ13305 | Homo sapiens hypothetical protein FLJ13305 (FLJ13305), |
| ANKRD36 | Homo sapiens ankyrin repeat domain 36 (ANKRD36), mRNA. |
| STRA6 | Homo sapiens stimulated by retinoic acid gene 6 homolog (mouse) (STRA6), mRNA. |
| LOC400451 | Homo sapiens hypothetical gene supported by AK075564; BC060873 (LOC400451), mRNA. |
| CASP6 | Homo sapiens caspase 6, apoptosis-related cysteine peptidase (CASP6), transcript variant alpha, mRNA. |
| NEDD4 | Homo sapiens neural precursor cell expressed, developmentally down-regulated 4 (NEDD4), transcript variant 2, mRNA. |
| ANAPC1 | Homo sapiens anaphase promoting complex subunit 1 (ANAPC1), mRNA. |
| TAOK1 | "Homo sapiens TAO kinase 1 (TAOK1), mRNA. |
| HEY1 | Homo sapiens hairy/enhancer-of-split related with YRPW motif 1 (HEY1), transcript variant 2, mRNA. |
| GNRH2 | Homo sapiens gonadotropin-releasing hormone 2 (GNRH2), transcript variant 1, mRNA. |
| EEF1B2 | Homo sapiens eukaryotic translation elongation factor 1 beta 2 (EEF1B2), transcript variant 2, mRNA. |
| SNX11 | Homo sapiens sorting nexin 11 (SNX11), transcript variant 1, mRNA. |
| LOC92196 | Homo sapiens death associated protein-like 1 (DAPL1), mRNA. |
| C1orf71 | Homo sapiens chromosome 1 open reading frame 71 (C1orf71), mRNA |
| TAGLN2 | Homo sapiens transgelin 2 (TAGLN2), mRNA. |
| TMEM101 | Homo sapiens transmembrane protein 101 (TMEM101), mRNA. |
| MUC13 | Homo sapiens mucin 13, cell surface associated (MUC13), mRNA. |
| MEP1A | Homo sapiens meprin A, alpha (PABA peptide hydrolase) (MEP1A), mRNA. |
| CPS1 | Homo sapiens carbamoyl-phosphate synthetase 1, mitochondrial (CPS1), mRNA. |
| FPRL1 | Homo sapiens formyl peptide receptor-like 1 (FPRL1), transcript variant 1, mRNA. |
| PHF1 | Homo sapiens PHD finger protein 1 (PHF1), transcript variant 2, mRNA. |
| TMEM39B | Homo sapiens transmembrane protein 39B (TMEM39B), mRNA. |
| LOC388389 | Homo sapiens coiled-coil domain containing 103 (CCDC103), mRNA. |
| SNORD56 | Homo sapiens small nucleolar RNA, C/D box 56 (SNORD56) on chromosome 20. |
| PARD6G | Homo sapiens par-6 partitioning defective 6 homolog gamma (C. elegans) (PARD6G), mRNA. |
| GOLGA | Homo sapiens golgin-like protein (GOLGA), mRNA. |
| PDIA5 | Homo sapiens protein disulfide isomerase family A, member 5 (PDIA5), mRNA. |
| DCI | Homo sapiens dodecenoyl-Coenzyme A delta isomerase (3,2 trans-enoyl-Coenzyme A isomerase) (DCI), nuclear gene encoding mitochondrial protein, mRNA. |
| FBXL5 | Homo sapiens F-box and leucine-rich repeat protein 5 (FBXL5), transcript variant 1, mRNA. |
| USP13 | Homo sapiens ubiquitin specific peptidase 13 (isopeptidase T-3) (USP13), mRNA. |
| C11orf54 | Homo sapiens chromosome 11 open reading frame 54 (C11orf54), mRNA. |
| ZNF452 | Homo sapiens zinc finger protein 452 (ZNF452), mRNA. |
| BRD9 | Homo sapiens bromodomain containing 9 (BRD9), transcript variant 1, mRNA. |
| FLJ22222 | Homo sapiens hypothetical protein FLJ22222 (FLJ22222), transcript variant 2, mRNA. |
| SGOL1 | Homo sapiens shugoshin-like 1 (S. pombe) (SGOL1), transcript variant C1, mRNA. |
| RIOK3 | Homo sapiens RIO kinase 3 (yeast) (RIOK3), transcript variant 1, mRNA. |
| GJC1 | Homo sapiens gap junction protein, chi 1, 31.9kDa (GJC1), mRNA. |
| CHRFAM7A | Homo sapiens CHRNA7 (cholinergic receptor, nicotinic, alpha 7, exons 5-10) and FAM7A (family with sequence similarity 7A, exons A-E) fusion (CHRFAM7A), transcript variant 1, mRNA. |
| ZP2 | Homo sapiens zona pellucida glycoprotein 2 (sperm receptor) (ZP2), mRNA. |
| EWSR1 | Homo sapiens Ewing sarcoma breakpoint region 1 (EWSR1), transcript variant EWS-b, mRNA. |
| D2HGDH | Homo sapiens D-2-hydroxyglutarate dehydrogenase (D2HGDH), nuclear gene encoding mitochondrial protein, mRNA. |
| GPR114 | Homo sapiens G protein-coupled receptor 114 (GPR114), mRNA. |
| ARRDC2 | Homo sapiens arrestin domain containing 2 (ARRDC2), transcript variant 1, mRNA. |
| PKD1 | Homo sapiens polycystic kidney disease 1 (autosomal dominant) (PKD1), transcript variant 1, mRNA." |
| CDK5RAP2 | Homo sapiens CDK5 regulatory subunit associated protein 2 (CDK5RAP2), transcript variant 2, mRNA." |
| PTPN7 | Homo sapiens protein tyrosine phosphatase, non-receptor type 7 (PTPN7), transcript variant 2, mRNA." |
| LATS1 | Homo sapiens LATS, large tumor suppressor, homolog 1 (Drosophila) (LATS1), mRNA. |
| LOC81691 | Homo sapiens exonuclease NEF-sp (LOC81691), mRNA |
| LMBRD2 | Homo sapiens LMBR1 domain containing 2 (LMBRD2), mRNA. |
| C1orf171 | Homo sapiens tRNA-yW synthesizing protein 3 homolog (S. cerevisiae) (TYW3), mRNA |
| PNPLA2 | Homo sapiens patatin-like phospholipase domain containing 2 (PNPLA2), mRNA. |
| MRPL20 | Homo sapiens mitochondrial ribosomal protein L20 (MRPL20), nuclear gene encoding mitochondrial protein, mRNA. |
| IQCK | Homo sapiens IQ motif containing K (IQCK), mRNA. |
| RASSF2 | Homo sapiens Ras association (RalGDS/AF-6) domain family 2 (RASSF2), transcript variant 2, mRNA. |
| PELI2 | Homo sapiens pellino homolog 2 (Drosophila) (PELI2), mRNA. |
| TMEM86B | Homo sapiens transmembrane protein 86B (TMEM86B), mRNA. |
| MMD | Homo sapiens monocyte to macrophage differentiation-associated (MMD), mRNA. |
| FUBP1 | Homo sapiens far upstream element (FUSE) binding protein 1 (FUBP1), mRNA. |
| COQ10A | Homo sapiens coenzyme Q10 homolog A (S. cerevisiae) (COQ10A), mRNA. |
| ENDOGL1 | Homo sapiens endonuclease G-like 1 (ENDOGL1), mRNA. |
| C11orf77 | Homo sapiens chromosome 11 open reading frame 77 (C11orf77), mRNA. |
| ZNF403 | Homo sapiens zinc finger protein 403 (ZNF403), mRNA. |
| ZMYM3 | Homo sapiens zinc finger, MYM-type 3 (ZMYM3), transcript variant 2, mRNA. |
| PLTP | Homo sapiens phospholipid transfer protein (PLTP), transcript variant 1, mRNA. |
| RNASET2 | Homo sapiens ribonuclease T2 (RNASET2), mRNA. |
| UBL3 | Homo sapiens ubiquitin-like 3 (UBL3), mRNA. |
| NUDT14 | Homo sapiens nudix (nucleoside diphosphate linked moiety X)-type motif 14 (NUDT14), mRNA. |
| TUBG1 | Homo sapiens tubulin, gamma 1 (TUBG1), mRNA. |
| MTMR2 | Homo sapiens myotubularin related protein 2 (MTMR2), transcript variant 3, mRNA. |
| PPP2R2B | Homo sapiens protein phosphatase 2 (formerly 2A), regulatory subunit B, beta isoform (PPP2R2B), transcript variant 5, mRNA. |
| ARMCX6 | Homo sapiens armadillo repeat containing, X-linked 6 (ARMCX6), transcript variant 1, mRNA. |
| ACTR2 | Homo sapiens ARP2 actin-related protein 2 homolog (yeast) (ACTR2), transcript variant 1, mRNA. |
| SPOP | Homo sapiens speckle-type POZ protein (SPOP), transcript variant 5, mRNA. |
| CGN | Homo sapiens cingulin (CGN), mRNA. |
| NKG7 | Homo sapiens natural killer cell group 7 sequence (NKG7), mRNA. |
| FLNB | Homo sapiens filamin B, beta (actin binding protein 278) (FLNB), mRNA. |
| SLC7A7 | Homo sapiens solute carrier family 7 (cationic amino acid transporter, y+ system), member 7 (SLC7A7), mRNA. |
| ARHGAP19 | Homo sapiens Rho GTPase activating protein 19 (ARHGAP19), mRNA. |
| GSG2 | Homo sapiens germ cell associated 2 (haspin) (GSG2), mRNA. |
| GPIAP1 | Homo sapiens cell cycle associated protein 1 (CAPRIN1), transcript variant 2, mRNA. |
| FLJ22471 | Homo sapiens coiled-coil domain containing 92 (CCDC92), mRNA. |
| MGC42174 | Homo sapiens DIS3 mitotic control homolog (S. cerevisiae)-like 2 (DIS3L2), mRNA. |
| C14orf150 | Homo sapiens WD repeat domain 89 (WDR89), transcript variant 2, mRNA. |
| BBX | Homo sapiens bobby sox homolog (Drosophila) (BBX), mRNA. |
| MRPL36 | Homo sapiens mitochondrial ribosomal protein L36 (MRPL36), nuclear gene encoding mitochondrial protein, mRNA |
| IL1F7 | Homo sapiens interleukin 1 family, member 7 (zeta) (IL1F7), transcript variant 1, mRNA. |
| STMN1 | Homo sapiens stathmin 1/oncoprotein 18 (STMN1), transcript variant 3, mRNA. |
| ARAF | Homo sapiens v-raf murine sarcoma 3611 viral oncogene homolog (ARAF), mRNA. |
| NDUFS7 | Homo sapiens NADH dehydrogenase (ubiquinone) Fe-S protein 7, 20kDa (NADH-coenzyme Q reductase) (NDUFS7), mRNA. |
| NASP | Homo sapiens nuclear autoantigenic sperm protein (histone-binding) (NASP), transcript variant 2, mRNA. |
| PTCH | "Homo sapiens patched homolog 1 (Drosophila) (PTCH1), mRNA. |
| KYNU | "Homo sapiens kynureninase (L-kynurenine hydrolase) (KYNU), transcript variant 1, mRNA. |
| TMEM103 | Homo sapiens transmembrane protein 103 (TMEM103), transcript variant 1, mRNA. |
| NDE1 | Homo sapiens nudE nuclear distribution gene E homolog 1 (A. nidulans) (NDE1), mRNA. |
| HIBADH | Homo sapiens 3-hydroxyisobutyrate dehydrogenase (HIBADH), mRNA. |
| DEXI | Homo sapiens dexamethasone-induced transcript (DEXI), mRNA. |
| PHTF1 | Homo sapiens putative homeodomain transcription factor 1 (PHTF1), mRNA. |
| BRCA2 | Homo sapiens breast cancer 2, early onset (BRCA2), mRNA. |
| C9orf37 | Homo sapiens chromosome 9 open reading frame 37 (C9orf37), mRNA. |
| MARCKS | Homo sapiens myristoylated alanine-rich protein kinase C substrate (MARCKS), mRNA |
| ATP5D | Homo sapiens ATP synthase, H+ transporting, mitochondrial F1 complex, delta subunit (ATP5D), nuclear gene encoding mitochondrial protein, transcript variant 1, mRNA. |
| TMEM11 | Homo sapiens transmembrane protein 11 (TMEM11), mRNA. |
| NCF2 | Homo sapiens neutrophil cytosolic factor 2 (65kDa, chronic granulomatous disease, autosomal 2) (NCF2), mRNA. |
| PDLIM5 | Homo sapiens PDZ and LIM domain 5 (PDLIM5), transcript variant 4, mRNA. |
| CIAS1 | Homo sapiens cold autoinflammatory syndrome 1 (CIAS1), transcript variant 1, mRNA." |
| FER | Homo sapiens fer (fps/fes related) tyrosine kinase (phosphoprotein NCP94) (FER), mRNA. |
| ZSWIM4 | Homo sapiens zinc finger, SWIM-type containing 4 (ZSWIM4), mRNA. |
| IL13RA1 | Homo sapiens interleukin 13 receptor, alpha 1 (IL13RA1), mRNA. |
| ARRB1 | Homo sapiens arrestin, beta 1 (ARRB1), transcript variant 1, mRNA. |
| KLHL8 | Homo sapiens kelch-like 8 (Drosophila) (KLHL8), mRNA. |
| RARSL | Homo sapiens arginyl-tRNA synthetase 2, mitochondrial (putative) (RARS2), mRNA. |
| HCFC1R1 | Homo sapiens host cell factor C1 regulator 1 (XPO1 dependent) (HCFC1R1), transcript variant 2, mRNA. |
| TMEM1 | Homo sapiens transmembrane protein 1 (TMEM1), transcript variant 2, mRNA. |
| NAPRT1 | Homo sapiens nicotinate phosphoribosyltransferase domain containing 1 (NAPRT1), mRNA. |
| CRYZ | Homo sapiens crystallin, zeta (quinone reductase) (CRYZ), mRNA. |
| ASCC3L1 | Homo sapiens activating signal cointegrator 1 complex subunit 3-like 1 (ASCC3L1), mRNA. |
| CSRP2BP | Homo sapiens CSRP2 binding protein (CSRP2BP), transcript variant 1, mRNA. |
| CGI-96 | Homo sapiens CGI-96 protein (CGI-96), mRNA. |
| IBRDC3 | Homo sapiens IBR domain containing 3 (IBRDC3), mRNA. |
| SETD4 | Homo sapiens SET domain containing 4 (SETD4), transcript variant 4, mRNA. |
| RP11-19J3.3 | Homo sapiens centromere protein P (CENPP), mRNA. |
| RFX3 | Homo sapiens regulatory factor X, 3 (influences HLA class II expression) (RFX3), transcript variant 2, mRNA. |
| TMEM93 | Homo sapiens transmembrane protein 93 (TMEM93), transcript variant 2, mRNA. |
| RAFTLIN | Homo sapiens raftlin, lipid raft linker 1 (RFTN1), mRNA. |
| RHEBL1 | Homo sapiens Ras homolog enriched in brain like 1 (RHEBL1), mRNA. |
| ASTN2 | Homo sapiens astrotactin 2 (ASTN2), transcript variant 4, mRNA. |
| LHPP | Homo sapiens phospholysine phosphohistidine inorganic pyrophosphate phosphatase (LHPP), mRNA. |
| LAMP1 | Homo sapiens lysosomal-associated membrane protein 1 (LAMP1), mRNA. |
| TMBIM1 | Homo sapiens transmembrane BAX inhibitor motif containing 1 (TMBIM1), mRNA. |
| EIF4EBP1 | Homo sapiens eukaryotic translation initiation factor 4E binding protein 1 (EIF4EBP1), mRNA. |
| PDE3B | Homo sapiens phosphodiesterase 3B, cGMP-inhibited (PDE3B), mRNA. |
| MTHFD1L | Homo sapiens methylenetetrahydrofolate dehydrogenase (NADP+ dependent) 1-like (MTHFD1L), mRNA. |
| C7orf25 | Homo sapiens chromosome 7 open reading frame 25 (C7orf25), mRNA. |
| FLJ44968 | Homo sapiens FLJ44968 protein (FLJ44968), mRNA. |
| MPV17 | Homo sapiens MpV17 transgene, murine homolog, glomerulosclerosis (MPV17), mRNA. |
| STK33 | Homo sapiens serine/threonine kinase 33 (STK33), mRNA." |
| ARPC2 | Homo sapiens actin related protein 2/3 complex, subunit 2, 34kDa (ARPC2), transcript variant 2, mRNA." |
| STK16 | Homo sapiens serine/threonine kinase 16 (STK16), transcript variant 2, mRNA." |
| SSBP4 | Homo sapiens single stranded DNA binding protein 4 (SSBP4), transcript variant 1, mRNA." |
| LSM7 | Homo sapiens LSM7 homolog, U6 small nuclear RNA associated (S. cerevisiae) (LSM7), mRNA." |
| TA-NFKBH | Homo sapiens T-cell activation NFKB-like protein (TA-NFKBH), mRNA. |
| C3orf21 | Homo sapiens chromosome 3 open reading frame 21 (C3orf21), mRNA. |
| LOC339229 | Homo sapiens hypothetical protein LOC339229 (LOC339229), mRNA. |
| IFIT2 | Homo sapiens interferon-induced protein with tetratricopeptide repeats 2 (IFIT2), mRNA. |
| IFIT3 | Homo sapiens interferon-induced protein with tetratricopeptide repeats 3 (IFIT3), mRNA. |
| CLDND2 | Homo sapiens claudin domain containing 2 (CLDND2), mRNA. |
| ADCK5 | Homo sapiens aarF domain containing kinase 5 (ADCK5), mRNA. |
| MTMR9 | Homo sapiens myotubularin related protein 9 (MTMR9), mRNA. |
| LTA | Homo sapiens lymphotoxin alpha (TNF superfamily, member 1) (LTA), mRNA. |
| RIMS2 | Homo sapiens regulating synaptic membrane exocytosis 2 (RIMS2), mRNA. |
| MASA | Homo sapiens enolase-phosphatase 1 (ENOPH1), mRNA. |
| MRPL27 | Homo sapiens mitochondrial ribosomal protein L27 (MRPL27), nuclear gene encoding mitochondrial protein, transcript variant 1, mRNA. |
| RDM1 | Homo sapiens RAD52 motif 1 (RDM1), transcript variant 2, mRNA. |
| MAPBPIP | Homo sapiens mitogen-activated protein-binding protein-interacting protein (MAPBPIP), mRNA. |
| PTD015 | Homo sapiens chromosome 11 open reading frame 67 (C11orf67), mRNA. |
| LRRC20 | Homo sapiens leucine rich repeat containing 20 (LRRC20), transcript variant 2, mRNA. |
| PTK2B | Homo sapiens PTK2B protein tyrosine kinase 2 beta (PTK2B), transcript variant 3, mRNA. |
| FOSB | Homo sapiens FBJ murine osteosarcoma viral oncogene homolog B (FOSB), mRNA. |
| SDCCAG10 | Homo sapiens serologically defined colon cancer antigen 10 (SDCCAG10), mRNA. |
