# Supplementary material for: Effect of Chemical Mutagens and Carcinogens on Gene Expression Profiles in Human TK6 Cells
Source: PLoS One. 2012 Jun 18;7(6):e39205. doi: 10.1371/journal.pone.0039205 (PMC3377624; doi:10.1371/journal.pone.0039205)
Supplement: Table S2 — Complete list of affected GO categories per chemical dose.Functional classification of significantly altered genes by exposure to carcinogens (S9-) at low, medium and high dose into gene ontology (GO) categories were statistically performed. (DOC) [file pone.0039205.s002.doc]

**Supplementary Table S2: Complete list of affected GO categories per chemical dose**

Functional classification of significantly altered genes by exposure to carcinogens (S9-) at low, medium and high dose into gene ontology (GO) categories were statistically performed.

1. GO processes affected at low dose
   1. GO process affected by Acrylamide

| **GO.ID** | **Term** |
| --- | --- |
| GO:0006470 | protein amino acid dephosphorylation |
| GO:0042789 | mRNA transcription from RNA polymerase I |
| GO:0045204 | MAPK export from nucleus |
| GO:0045209 | MAPK phosphatase export from nucleus,le |
| GO:0006954 | inflammatory response |
| GO:0032792 | inhibition of CREB transcription factor |
| GO:0002376 | immune system process |
| GO:0009440 | cyanate catabolic process |
| GO:0046967 | cytosol to ER transport |
| GO:0001892 | embryonic placenta development |
| GO:0001975 | response to amphetamine |
| GO:0046686 | response to cadmium ion |
| GO:0008615 | pyridoxine biosynthetic process |
| GO:0006983 | ER overload response |
| GO:0046649 | lymphocyte activation |
| GO:0006469 | negative regulation of protein kinase ac... |
| GO:0043124 | negative regulation of I-kappaB kinase/N... |
| GO:0006417 | regulation of translation |
| GO:0009070 | serine family amino acid biosynthetic pr... |
| GO:0006857 | oligopeptide transport |
| GO:0019885 | antigen processing and presentation of e... |
| GO:0006950 | response to stress |
| GO:0030503 | regulation of cell redox homeostasis |
| GO:0033344 | cholesterol efflux |
| GO:0045408 | regulation of interleukin-6 biosynthetic... |
| GO:0007050 | cell cycle arrest |
| GO:0019363 | pyridine nucleotide biosynthetic process |
| GO:0006783 | heme biosynthetic process |
| GO:0008206 | bile acid metabolic process |

- 1. GO process affected by Benzo[a]fluoranthene

| **GO.ID** | | **Term** |
| --- | --- | --- |
| GO:0015942 | formate metabolic process | |
| GO:0001306 | age-dependent response to oxidative stre... | |
| GO:0007600 | sensory perception | |
| GO:0006419 | alanyl-tRNA aminoacylation | |
| GO:0018348 | protein amino acid geranylgeranylation | |
| GO:0051935 | glutamate uptake during transmission of ... | |
| GO:0021522 | spinal cord motor neuron differentiation | |
| GO:0035176 | social behavior | |
| GO:0050884 | neuromuscular process controlling postur... | |
| GO:0060041 | retina development in camera-type eye | |
| GO:0008306 | associative learning | |
| GO:0048535 | lymph node development | |
| GO:0060052 | neurofilament cytoskeleton organization ... | |
| GO:0044257 | cellular protein catabolic process | |
| GO:0045861 | negative regulation of proteolysis | |
| GO:0051291 | protein heterooligomerization | |
| GO:0007628 | adult walking behavior | |
| GO:0009396 | folic acid and derivative biosynthetic p... | |
| GO:0043303 | mast cell degranulation | |
| GO:0046513 | ceramide biosynthetic process | |
| GO:0001501 | skeletal development | |
| GO:0007601 | visual perception | |
| GO:0043087 | regulation of GTPase activity | |
| GO:0007040 | lysosome organization and biogenesis | |
| GO:0006479 | protein amino acid methylation | |
| GO:0007006 | mitochondrial membrane organization and ... | |
| GO:0050885 | neuromuscular process controlling balanc... | |
| GO:0006904 | vesicle docking during exocytosis | |
| GO:0042476 | odontogenesis | |
| GO:0016338 | calcium-independent cell-cell adhesion | |
| GO:0006953 | acute-phase response | |
| GO:0050909 | sensory perception of taste | |
| GO:0007606 | sensory perception of chemical stimulus | |
| GO:0006888 | ER to Golgi vesicle-mediated transport | |
| GO:0030521 | androgen receptor signaling pathway | |
| GO:0006511 | ubiquitin-dependent protein catabolic pr... | |
| GO:0032313 | regulation of Rab GTPase activity | |
| GO:0008360 | regulation of cell shape | |
| GO:0006836 | neurotransmitter transport | |
| GO:0008033 | tRNA processing | |
| GO:0008285 | negative regulation of cell proliferatio... | |
| GO:0051348 | negative regulation of transferase activ... | |
| GO:0007186 | G-protein coupled receptor protein signa... | |
| GO:0006512 | ubiquitin cycle | |
| GO:0008283 | cell proliferation | |
| GO:0008203 | cholesterol metabolic process | |
| GO:0048856 | anatomical structure development | |
| GO:0007608 | sensory perception of smell | |
| GO:0006817 | phosphate transport | |
| GO:0006464 | protein modification process | |
| GO:0048731 | system development | |
| GO:0006869 | lipid transport | |
| GO:0043412 | biopolymer modification | |
| GO:0007154 | cell communication | |
| GO:0043687 | post-translational protein modification | |
| GO:0007242 | intracellular signaling cascade | |
| GO:0007165 | signal transduction | |
| GO:0009887 | organ morphogenesis | |
| GO:0006486 | protein amino acid glycosylation | |
| GO:0051246 | regulation of protein metabolic process | |
| GO:0050896 | response to stimulus | |
| GO:0008544 | epidermis development | |
| GO:0016042 | lipid catabolic process | |
| GO:0007605 | sensory perception of sound | |
| GO:0006644 | phospholipid metabolic process | |
| GO:0045786 | negative regulation of cell cycle | |
| GO:0006813 | potassium ion transport | |
| GO:0007155 | cell adhesion | |
| GO:0008361 | regulation of cell size | |
|  |  | |

- 1. GO process affected by Benz[a]anthracene

| **GO.ID** | **Term** |
| --- | --- |
| GO:0046726 | positive regulation of viral protein lev... |
| GO:0050689 | negative regulation of defense response ... |
| GO:0007017 | microtubule-based process |
| GO:0015785 | UDP-galactose transport |
| GO:0032383 | regulation of intracellular cholesterol ... |
| GO:0009440 | cyanate catabolic process |
| GO:0046782 | regulation of viral transcription |
| GO:0045070 | positive regulation of viral genome repl... |
| GO:0000085 | G2 phase of mitotic cell cycle |
| GO:0006469 | negative regulation of protein kinase ac... |
| GO:0006661 | phosphatidylinositol biosynthetic proces... |
| GO:0000041 | transition metal ion transport |
| GO:0007049 | cell cycle |
| GO:0046339 | diacylglycerol metabolic process |
| GO:0006977 | DNA damage response, signal transduction... |
| GO:0006012 | galactose metabolic process |
| GO:0042147 | retrograde transport, endosome to Golgi |
| GO:0006355 | regulation of transcription, DNA-depende... |
| GO:0006350 | transcription |
| GO:0006366 | transcription from RNA polymerase II pro... |
| GO:0051252 | regulation of RNA metabolic process |
| GO:0006512 | ubiquitin cycle |
| GO:0048193 | Golgi vesicle transport |
| GO:0007067 | mitosis |
| GO:0006351 | transcription, DNA-dependent |
| GO:0032774 | RNA biosynthetic process |
| GO:0019363 | pyridine nucleotide biosynthetic process |
| GO:0044238 | primary metabolic process |

- 1. GO process affected by Benzo[a]pyrene

| **GO.ID** | **Term** |
| --- | --- |
| GO:0008380 | RNA splicing |
| GO:0042113 | B cell activation |
| GO:0000722 | telomere maintenance via recombination |
| GO:0002827 | positive regulation of T-helper 1 type i... |
| GO:0006450 | regulation of translational fidelity |
| GO:0015942 | formate metabolic process |
| GO:0045204 | MAPK export from nucleus |
| GO:0045209 | MAPK phosphatase export from nucleus, le... |
| GO:0007163 | establishment and/or maintenance of cell... |
| GO:0001808 | negative regulation of type IV hypersens... |
| GO:0032438 | melanosome organization and biogenesis |
| GO:0045060 | negative thymic T cell selection |
| GO:0050823 | peptide antigen stabilization |
| GO:0002829 | negative regulation of T-helper 2 type i... |
| GO:0048302 | regulation of isotype switching to IgG i... |
| GO:0006470 | protein amino acid dephosphorylation |
| GO:0001562 | response to protozoan |
| GO:0032729 | positive regulation of interferon-gamma ... |
| GO:0032927 | positive regulation of activin receptor ... |
| GO:0042535 | positive regulation of tumor necrosis fa... |
| GO:0006397 | mRNA processing |
| GO:0009952 | anterior/posterior pattern formation |
| GO:0009108 | coenzyme biosynthetic process |
| GO:0031295 | T cell costimulation |
| GO:0050830 | defense response to Gram-positive bacter... |
| GO:0050688 | regulation of defense response to virus |
| GO:0006796 | phosphate metabolic process |
| GO:0042742 | defense response to bacterium |
| GO:0006895 | Golgi to endosome transport |
| GO:0019885 | antigen processing and presentation of e... |
| GO:0042130 | negative regulation of T cell proliferat... |
| GO:0031532 | actin cytoskeleton reorganization |
| GO:0050729 | positive regulation of inflammatory resp... |
| GO:0006607 | NLS-bearing substrate import into nucleu... |
| GO:0006890 | retrograde vesicle-mediated transport, G... |
| GO:0048518 | positive regulation of biological proces... |

- 1. GO process affected by Benzene

| **GO.ID** | **Term** |
| --- | --- |
| GO:0043065 | positive regulation of apoptosis |
| GO:0006563 | L-serine metabolic process |
| GO:0006412 | translation |
| GO:0009070 | serine family amino acid biosynthetic pr... |
| GO:0030503 | regulation of cell redox homeostasis |
| GO:0050678 | regulation of epithelial cell proliferat... |
| GO:0006396 | RNA processing |
| GO:0006450 | regulation of translational fidelity |
| GO:0006529 | asparagine biosynthetic process |
| GO:0010269 | response to selenium ion |
| GO:0022617 | extracellular matrix disassembly |
| GO:0031293 | membrane protein intracellular domain pr... |
| GO:0031581 | hemidesmosome assembly |
| GO:0042789 | mRNA transcription from RNA polymerase I... |
| GO:0045204 | MAPK export from nucleus |
| GO:0045209 | MAPK phosphatase export from nucleus, le... |
| GO:0016311 | dephosphorylation |
| GO:0006790 | sulfur metabolic process |
| GO:0007527 | adult somatic muscle development |
| GO:0030037 | actin filament reorganization during cel... |
| GO:0032792 | inhibition of CREB transcription factor |
| GO:0046653 | tetrahydrofolate metabolic process |
| GO:0046874 | quinolinate metabolic process |
| GO:0051491 | positive regulation of filopodium format... |
| GO:0042542 | response to hydrogen peroxide |
| GO:0000188 | inactivation of MAPK activity |
| GO:0000288 | mRNA catabolic process, deadenylation-de... |
| GO:0001958 | endochondral ossification |
| GO:0006419 | alanyl-tRNA aminoacylation |
| GO:0006478 | peptidyl-tyrosine sulfation |
| GO:0006601 | creatine biosynthetic process |
| GO:0016925 | protein sumoylation |
| GO:0042149 | cellular response to glucose starvation |
| GO:0042989 | sequestering of actin monomers |
| GO:0051497 | negative regulation of stress fiber form... |
| GO:0007568 | aging |
| GO:0000060 | protein import into nucleus, translocati... |
| GO:0051017 | actin filament bundle formation |
| GO:0015986 | ATP synthesis coupled proton transport |
| GO:0045892 | negative regulation of transcription, DN... |
| GO:0000002 | mitochondrial genome maintenance |
| GO:0001892 | embryonic placenta development |
| GO:0001975 | response to amphetamine |
| GO:0007518 | myoblast cell fate determination |
| GO:0043089 | positive regulation of Cdc42 GTPase acti... |
| GO:0065002 | intracellular protein transport across a... |
| GO:0006457 | protein folding |

- 1. GO process affected by Carbontetrachloride

| **GO.ID** | **Term** |
| --- | --- |
| GO:0006878 | cellular copper ion homeostasis |
| GO:0006825 | copper ion transport |
| GO:0019883 | antigen processing and presentation of e... |
| GO:0006099 | tricarboxylic acid cycle |
| GO:0015986 | ATP synthesis coupled proton transport |
| GO:0010269 | response to selenium ion |
| GO:0045919 | positive regulation of cytolysis |
| GO:0033599 | regulation of mammary gland epithelial c... |
| GO:0006107 | oxaloacetate metabolic process |
| GO:0046641 | positive regulation of alpha-beta T cell... |
| GO:0050823 | peptide antigen stabilization |
| GO:0043066 | negative regulation of apoptosis |
| GO:0000085 | G2 phase of mitotic cell cycle |
| GO:0008631 | induction of apoptosis by oxidative stre... |
| GO:0043030 | regulation of macrophage activation |
| GO:0006108 | malate metabolic process |
| GO:0009650 | UV protection |
| GO:0008535 | respiratory chain complex IV assembly |
| GO:0019674 | NAD metabolic process |
| GO:0006977 | DNA damage response, signal transduction... |
| GO:0018279 | protein amino acid N-linked glycosylatio... |
| GO:0019048 | virus-host interaction |
| GO:0019885 | antigen processing and presentation of e... |
| GO:0006917 | induction of apoptosis |
| GO:0048193 | Golgi vesicle transport |
| GO:0043154 | negative regulation of caspase activity |
| GO:0008632 | apoptotic program |
| GO:0012501 | programmed cell death |
| GO:0006979 | response to oxidative stress |
| GO:0030503 | regulation of cell redox homeostasis |
| GO:0042088 | T-helper 1 type immune response |
| GO:0045078 | positive regulation of interferon-gamma ... |
| GO:0001516 | prostaglandin biosynthetic process |
| GO:0008625 | induction of apoptosis via death domain ... |
| GO:0042744 | hydrogen peroxide catabolic process |
| GO:0008219 | cell death |
| GO:0008285 | negative regulation of cell proliferatio... |
| GO:0030163 | protein catabolic process |
| GO:0001836 | release of cytochrome c from mitochondri... |
| GO:0045058 | T cell selection |
| GO:0019363 | pyridine nucleotide biosynthetic process |
| GO:0008344 | adult locomotory behavior |
| GO:0050819 | negative regulation of coagulation |

- 1. GO process affected by Cyclophosphamide

| **GO.ID** | **Term** |
| --- | --- |
| GO:0001711 | endodermal cell fate commitment |
| GO:0030858 | positive regulation of epithelial cell d... |
| GO:0001709 | cell fate determination |
| GO:0000910 | cytokinesis |
| GO:0000921 | septin ring assembly |
| GO:0001706 | endoderm formation |
| GO:0007527 | adult somatic muscle development |
| GO:0015785 | UDP-galactose transport |
| GO:0032331 | negative regulation of chondrocyte diffe... |
| GO:0030323 | respiratory tube development |
| GO:0001833 | inner cell mass cell proliferation |
| GO:0035117 | embryonic arm morphogenesis |
| GO:0048260 | positive regulation of receptor-mediated... |
| GO:0000085 | G2 phase of mitotic cell cycle |
| GO:0009950 | dorsal/ventral axis specification |
| GO:0032205 | negative regulation of telomere maintena... |
| GO:0009954 | proximal/distal pattern formation |
| GO:0035116 | embryonic hindlimb morphogenesis |
| GO:0045669 | positive regulation of osteoblast differ... |
| GO:0060070 | Wnt receptor signaling pathway through b... |
| GO:0006413 | translational initiation |
| GO:0045671 | negative regulation of osteoclast differ... |
| GO:0051291 | protein heterooligomerization |
| GO:0006977 | DNA damage response, signal transduction... |
| GO:0042733 | embryonic digit morphogenesis |
| GO:0006012 | galactose metabolic process |
| GO:0001569 | patterning of blood vessels |
| GO:0001837 | epithelial to mesenchymal transition |
| GO:0031016 | pancreas development |
| GO:0007157 | heterophilic cell adhesion |
| GO:0050729 | positive regulation of inflammatory resp... |
| GO:0007004 | telomere maintenance via telomerase |
| GO:0019363 | pyridine nucleotide biosynthetic process |
| GO:0048593 | camera-type eye morphogenesis |
| GO:0045069 | regulation of viral genome replication |
| GO:0045453 | bone resorption |

- 1. GO process affected by Formaldehyde

| **GO.ID** | **Term** |
| --- | --- |
| GO:0043412 | biopolymer modification |
| GO:0019885 | antigen processing and presentation of e... |
| GO:0045767 | regulation of anti-apoptosis |
| GO:0000072 | M phase specific microtubule process |
| GO:0006450 | regulation of translational fidelity |
| GO:0045023 | G0 to G1 transition |
| GO:0006270 | DNA replication initiation |
| GO:0006552 | leucine catabolic process |
| GO:0019896 | axon transport of mitochondrion |
| GO:0006464 | protein modification process |
| GO:0016310 | phosphorylation |
| GO:0000266 | mitochondrial fission |
| GO:0008053 | mitochondrial fusion |
| GO:0046967 | cytosol to ER transport |
| GO:0050823 | peptide antigen stabilization |
| GO:0001833 | inner cell mass cell proliferation |
| GO:0043687 | post-translational protein modification |
| GO:0000085 | G2 phase of mitotic cell cycle |
| GO:0043249 | erythrocyte maturation |
| GO:0007605 | sensory perception of sound |
| GO:0006352 | transcription initiation |
| GO:0007220 | Notch receptor processing |
| GO:0042177 | negative regulation of protein catabolic... |
| GO:0006474 | N-terminal protein amino acid acetylatio... |
| GO:0006857 | oligopeptide transport |
| GO:0007007 | inner mitochondrial membrane organizatio... |
| GO:0048193 | Golgi vesicle transport |
| GO:0006509 | membrane protein ectodomain proteolysis |
| GO:0042987 | amyloid precursor protein catabolic proc... |
| GO:0007050 | cell cycle arrest |
| GO:0006607 | NLS-bearing substrate import into nucleu... |

- 1. GO process affected by Hydroquinone

| **GO.ID** | **Term** |
| --- | --- |
| GO:0007569 | cell aging |
| GO:0030330 | DNA damage response, signal transduction... |
| GO:0033595 | response to genistein |
| GO:0033600 | negative regulation of mammary gland epi... |
| GO:0051017 | actin filament bundle formation |
| GO:0019896 | axon transport of mitochondrion |
| GO:0050860 | negative regulation of T cell receptor s... |
| GO:0000266 | mitochondrial fission |
| GO:0001556 | oocyte maturation |
| GO:0001880 | Mullerian duct regression |
| GO:0007141 | male meiosis I |
| GO:0008053 | mitochondrial fusion |
| GO:0010225 | response to UV-C |
| GO:0046641 | positive regulation of alpha-beta T cell... |
| GO:0048478 | replication fork protection |
| GO:0050823 | peptide antigen stabilization |
| GO:0006464 | protein modification process |
| GO:0001833 | inner cell mass cell proliferation |
| GO:0007044 | cell-substrate junction assembly |
| GO:0042752 | regulation of circadian rhythm |
| GO:0000085 | G2 phase of mitotic cell cycle |
| GO:0030817 | regulation of cAMP biosynthetic process |
| GO:0033205 | cytokinesis during cell cycle |
| GO:0010332 | response to gamma radiation |
| GO:0045931 | positive regulation of mitotic cell cycl... |
| GO:0051298 | centrosome duplication |
| GO:0047497 | mitochondrion transport along microtubul... |
| GO:0007007 | inner mitochondrial membrane organizatio... |
| GO:0010165 | response to X-ray |
| GO:0019885 | antigen processing and presentation of e... |
| GO:0006477 | protein amino acid sulfation |
| GO:0007017 | microtubule-based process |
| GO:0042088 | T-helper 1 type immune response |
| GO:0045078 | positive regulation of interferon-gamma ... |
| GO:0016573 | histone acetylation |
| GO:0045768 | positive regulation of anti-apoptosis |
| GO:0016568 | chromatin modification |
| GO:0007090 | regulation of S phase of mitotic cell cy... |
| GO:0007613 | memory |
| GO:0006468 | protein amino acid phosphorylation |
| GO:0000724 | double-strand break repair via homologou... |
| GO:0006890 | retrograde vesicle-mediated transport, G... |

- 1. GO process affected by Mitomycin C

| GO.ID | Term |
| --- | --- |
| GO:0050891 | multicellular organismal water homeostas... |
| GO:0030178 | negative regulation of Wnt receptor sign... |
| GO:0031583 | G-protein signaling, phospholipase D act... |
| GO:0042313 | protein kinase C deactivation |
| GO:0043179 | rhythmic excitation |
| GO:0051771 | negative regulation of nitric-oxide synt... |
| GO:0007506 | gonadal mesoderm development |
| GO:0015785 | UDP-galactose transport |
| GO:0030185 | nitric oxide transport |
| GO:0030818 | negative regulation of cAMP biosynthetic... |
| GO:0032383 | regulation of intracellular cholesterol ... |
| GO:0000288 | mRNA catabolic process, deadenylation-de... |
| GO:0001880 | Mullerian duct regression |
| GO:0009440 | cyanate catabolic process |
| GO:0042474 | middle ear morphogenesis |
| GO:0045730 | respiratory burst |
| GO:0008054 | cyclin catabolic process |
| GO:0030147 | natriuresis |
| GO:0055007 | cardiac muscle cell differentiation |
| GO:0000085 | G2 phase of mitotic cell cycle |
| GO:0030146 | diuresis |
| GO:0043488 | regulation of mRNA stability |
| GO:0006950 | response to stress |
| GO:0014065 | phosphoinositide 3-kinase cascade |
| GO:0042119 | neutrophil activation |
| GO:0007204 | elevation of cytosolic calcium ion conce... |
| GO:0045321 | leukocyte activation |
| GO:0007567 | parturition |
| GO:0006857 | oligopeptide transport |
| GO:0006977 | DNA damage response, signal transduction... |
| GO:0019229 | regulation of vasoconstriction |
| GO:0006012 | galactose metabolic process |
| GO:0042147 | retrograde transport, endosome to Golgi |
| GO:0051899 | membrane depolarization |
| GO:0003002 | regionalization |
| GO:0006903 | vesicle targeting |
| GO:0048661 | positive regulation of smooth muscle cel... |
| GO:0001569 | patterning of blood vessels |
| GO:0046887 | positive regulation of hormone secretion |
| GO:0046888 | negative regulation of hormone secretion |
| GO:0030195 | negative regulation of blood coagulation |
| GO:0015671 | oxygen transport |
| GO:0019363 | pyridine nucleotide biosynthetic process |
| GO:0045840 | positive regulation of mitosis |
| GO:0006783 | heme biosynthetic process |
| GO:0007528 | neuromuscular junction development |
| GO:0007507 | heart development |
| GO:0014032 | neural crest cell development |

- 1. GO process affected by Styrene

| **GO.ID** | **Term** |
| --- | --- |
| GO:0010460 | positive regulation of heart rate |
| GO:0042088 | T-helper 1 type immune response |
| GO:0009074 | aromatic amino acid family catabolic pro... |
| GO:0008156 | negative regulation of DNA replication |
| GO:0050731 | positive regulation of peptidyl-tyrosine... |
| GO:0030317 | sperm motility |
| GO:0006550 | isoleucine catabolic process |
| GO:0009098 | leucine biosynthetic process |
| GO:0014061 | regulation of norepinephrine secretion |
| GO:0033600 | negative regulation of mammary gland epi... |
| GO:0042508 | tyrosine phosphorylation of Stat1 protei... |
| GO:0048149 | behavioral response to ethanol |
| GO:0048266 | behavioral response to pain |
| GO:0060112 | generation of ovulation cycle rhythm |
| GO:0051017 | actin filament bundle formation |
| GO:0009266 | response to temperature stimulus |
| GO:0001997 | positive regulation of the force of hear... |
| GO:0002024 | diet induced thermogenesis |
| GO:0009386 | translational attenuation |
| GO:0019673 | GDP-mannose metabolic process |
| GO:0032225 | regulation of synaptic transmission, dop... |
| GO:0032720 | negative regulation of tumor necrosis fa... |
| GO:0042351 | 'de novo' GDP-L-fucose biosynthetic proc... |
| GO:0042133 | neurotransmitter metabolic process |
| GO:0006917 | induction of apoptosis |
| GO:0042108 | positive regulation of cytokine biosynth... |
| GO:0001556 | oocyte maturation |
| GO:0006978 | DNA damage response, signal transduction... |
| GO:0007141 | male meiosis I |
| GO:0009440 | cyanate catabolic process |
| GO:0010225 | response to UV-C |
| GO:0032020 | ISG15-protein conjugation |
| GO:0032715 | negative regulation of interleukin-6 pro... |
| GO:0045627 | positive regulation of T-helper 1 cell d... |
| GO:0046641 | positive regulation of alpha-beta T cell... |
| GO:0050823 | peptide antigen stabilization |
| GO:0001833 | inner cell mass cell proliferation |
| GO:0031649 | heat generation |
| GO:0032691 | negative regulation of interleukin-1 bet... |
| GO:0040015 | negative regulation of multicellular org... |
| GO:0042506 | tyrosine phosphorylation of Stat5 protei... |
| GO:0033138 | positive regulation of peptidyl-serine p... |
| GO:0033205 | cytokinesis during cell cycle |
| GO:0042416 | dopamine biosynthetic process |
| GO:0050873 | brown fat cell differentiation |
| GO:0050890 | cognition |
| GO:0008306 | associative learning |
| GO:0031667 | response to nutrient levels |
| GO:0006955 | immune response |
| GO:0042113 | B cell activation |
| GO:0045776 | negative regulation of blood pressure |
| GO:0006729 | tetrahydrobiopterin biosynthetic process |
| GO:0009435 | NAD biosynthetic process |
| GO:0010332 | response to gamma radiation |
| GO:0031529 | ruffle organization and biogenesis |
| GO:0045931 | positive regulation of mitotic cell cycl... |
| GO:0051298 | centrosome duplication |
| GO:0016337 | cell-cell adhesion |
| GO:0042596 | fear response |
| GO:0010165 | response to X-ray |
| GO:0019885 | antigen processing and presentation of e... |
| GO:0009967 | positive regulation of signal transducti... |
| GO:0014070 | response to organic cyclic substance |
| GO:0042503 | tyrosine phosphorylation of Stat3 protei... |
| GO:0042771 | DNA damage response, signal transduction... |
| GO:0007569 | cell aging |
| GO:0046888 | negative regulation of hormone secretion |
| GO:0048193 | Golgi vesicle transport |
| GO:0007090 | regulation of S phase of mitotic cell cy... |
| GO:0007613 | memory |
| GO:0050728 | negative regulation of inflammatory resp... |
| GO:0007160 | cell-matrix adhesion |
| GO:0000724 | double-strand break repair via homologou... |
| GO:0006607 | NLS-bearing substrate import into nucleu... |
| GO:0007271 | synaptic transmission, cholinergic |
| GO:0045766 | positive regulation of angiogenesis |

- 1. GO process affected by Styrene 7,8-oxide

| **GO.ID** | **Term** |
| --- | --- |
| GO:0001711 | endodermal cell fate commitment |
| GO:0030858 | positive regulation of epithelial cell d... |
| GO:0001706 | endoderm formation |
| GO:0001808 | negative regulation of type IV hypersens... |
| GO:0045060 | negative thymic T cell selection |
| GO:0006259 | DNA metabolic process |
| GO:0015805 | S-adenosylmethionine transport |
| GO:0018348 | protein amino acid geranylgeranylation |
| GO:0032331 | negative regulation of chondrocyte diffe... |
| GO:0048469 | cell maturation |
| GO:0007042 | lysosomal lumen acidification |
| GO:0030150 | protein import into mitochondrial matrix |
| GO:0035117 | embryonic arm morphogenesis |
| GO:0000122 | negative regulation of transcription fro... |
| GO:0000012 | single strand break repair |
| GO:0001562 | response to protozoan |
| GO:0009950 | dorsal/ventral axis specification |
| GO:0042535 | positive regulation of tumor necrosis fa... |
| GO:0048662 | negative regulation of smooth muscle cel... |
| GO:0032989 | cellular structure morphogenesis |
| GO:0009954 | proximal/distal pattern formation |
| GO:0031295 | T cell costimulation |
| GO:0035116 | embryonic hindlimb morphogenesis |
| GO:0045669 | positive regulation of osteoblast differ... |
| GO:0060070 | Wnt receptor signaling pathway through b... |
| GO:0006796 | phosphate metabolic process |
| GO:0000389 | nuclear mRNA 3'-splice site recognition |
| GO:0015858 | nucleoside transport |
| GO:0045671 | negative regulation of osteoclast differ... |
| GO:0016310 | phosphorylation |
| GO:0050688 | regulation of defense response to virus |
| GO:0000398 | nuclear mRNA splicing, via spliceosome |
| GO:0042733 | embryonic digit morphogenesis |
| GO:0006468 | protein amino acid phosphorylation |
| GO:0042130 | negative regulation of T cell proliferat... |
| GO:0001569 | patterning of blood vessels |
| GO:0001837 | epithelial to mesenchymal transition |
| GO:0031016 | pancreas development |
| GO:0030036 | actin cytoskeleton organization and biog... |
| GO:0048593 | camera-type eye morphogenesis |
| GO:0050772 | positive regulation of axonogenesis |

- 1. GO process affected by Trichloroethylene

| **GO.ID** | **Term** |
| --- | --- |
| GO:0045624 | positive regulation of T-helper cell dif... |
| GO:0045086 | positive regulation of interleukin-2 bio... |
| GO:0007530 | sex determination |
| GO:0045908 | negative regulation of vasodilation |
| GO:0001996 | positive regulation of heart rate by epi... |
| GO:0001997 | positive regulation of the force of hear... |
| GO:0002024 | diet induced thermogenesis |
| GO:0002025 | vasodilation by norepinephrine-epinephri... |
| GO:0007506 | gonadal mesoderm development |
| GO:0043017 | positive regulation of lymphotoxin A bio... |
| GO:0051000 | positive regulation of nitric-oxide synt... |
| GO:0015758 | glucose transport |
| GO:0001880 | Mullerian duct regression |
| GO:0006569 | tryptophan catabolic process |
| GO:0045404 | positive regulation of interleukin-4 bio... |
| GO:0045425 | positive regulation of granulocyte macro... |
| GO:0050823 | peptide antigen stabilization |
| GO:0031649 | heat generation |
| GO:0032228 | regulation of synaptic transmission, GAB... |
| GO:0035022 | positive regulation of Rac protein signa... |
| GO:0040015 | negative regulation of multicellular org... |
| GO:0007267 | cell-cell signaling |
| GO:0018108 | peptidyl-tyrosine phosphorylation |
| GO:0000085 | G2 phase of mitotic cell cycle |
| GO:0045909 | positive regulation of vasodilation |
| GO:0048011 | nerve growth factor receptor signaling p... |
| GO:0050873 | brown fat cell differentiation |
| GO:0042692 | muscle cell differentiation |
| GO:0006166 | purine ribonucleoside salvage |
| GO:0042177 | negative regulation of protein catabolic... |
| GO:0048169 | regulation of long-term neuronal synapti... |
| GO:0009435 | NAD biosynthetic process |
| GO:0015858 | nucleoside transport |
| GO:0016079 | synaptic vesicle exocytosis |
| GO:0008542 | visual learning |
| GO:0009409 | response to cold |
| GO:0030539 | male genitalia development |
| GO:0042596 | fear response |
| GO:0006977 | DNA damage response, signal transduction... |
| GO:0019885 | antigen processing and presentation of e... |
| GO:0032868 | response to insulin stimulus |
| GO:0042355 | L-fucose catabolic process |
| GO:0006360 | transcription from RNA polymerase I prom... |
| GO:0045429 | positive regulation of nitric oxide bios... |
| GO:0048009 | insulin-like growth factor receptor sign... |
| GO:0006890 | retrograde vesicle-mediated transport, G... |
| GO:0017156 | calcium ion-dependent exocytosis |

1. GO processes affected at medium dose
   1. GO process affected by Benz[a]anthracene

| **GO.ID** | **Term** |
| --- | --- |
| GO:0001808 | negative regulation of type IV hypersens... |
| GO:0045060 | negative thymic T cell selection |
| GO:0006509 | membrane protein ectodomain proteolysis |
| GO:0042987 | amyloid precursor protein catabolic proc... |
| GO:0001562 | response to protozoan |
| GO:0042535 | positive regulation of tumor necrosis fa... |
| GO:0000080 | G1 phase of mitotic cell cycle |
| GO:0031295 | T cell costimulation |
| GO:0006915 | apoptosis |
| GO:0006968 | cellular defense response |
| GO:0019885 | antigen processing and presentation of e... |
| GO:0008284 | positive regulation of cell proliferatio... |
| GO:0007050 | cell cycle arrest |
| GO:0042130 | negative regulation of T cell proliferat... |
| GO:0006270 | DNA replication initiation |
| GO:0008624 | induction of apoptosis by extracellular ... |
| GO:0042417 | dopamine metabolic process |
| GO:0001711 | endodermal cell fate commitment |
| GO:0001922 | B-1 B cell homeostasis |
| GO:0001988 | positive regulation of heart rate in bar... |
| GO:0006450 | regulation of translational fidelity |
| GO:0014061 | regulation of norepinephrine secretion |
| GO:0030858 | positive regulation of epithelial cell d... |
| GO:0031293 | membrane protein intracellular domain pr... |
| GO:0032469 | endoplasmic reticulum calcium ion homeos... |
| GO:0045023 | G0 to G1 transition |
| GO:0048149 | behavioral response to ethanol |
| GO:0060112 | generation of ovulation cycle rhythm |
| GO:0016310 | phosphorylation |
| GO:0006801 | superoxide metabolic process |
| GO:0000086 | G2/M transition of mitotic cell cycle |
| GO:0006457 | protein folding |
| GO:0006916 | anti-apoptosis |
| GO:0001706 | endoderm formation |
| GO:0006552 | leucine catabolic process |
| GO:0006565 | L-serine catabolic process |
| GO:0015827 | tryptophan transport |
| GO:0032225 | regulation of synaptic transmission, dop... |
| GO:0032720 | negative regulation of tumor necrosis fa... |
| GO:0046080 | dUTP metabolic process |
| GO:0048489 | synaptic vesicle transport |
| GO:0050821 | protein stabilization |
| GO:0045454 | cell redox homeostasis |
| GO:0006420 | arginyl-tRNA aminoacylation |
| GO:0006601 | creatine biosynthetic process |
| GO:0032331 | negative regulation of chondrocyte diffe... |
| GO:0032715 | negative regulation of interleukin-6 pro... |
| GO:0046967 | cytosol to ER transport |
| GO:0050823 | peptide antigen stabilization |
| GO:0042102 | positive regulation of T cell proliferat... |
| GO:0030384 | phosphoinositide metabolic process |
| GO:0006654 | phosphatidic acid biosynthetic process |
| GO:0006930 | substrate-bound cell migration, cell ext... |
| GO:0008054 | cyclin catabolic process |
| GO:0008634 | negative regulation of survival gene pro... |
| GO:0032691 | negative regulation of interleukin-1 bet... |
| GO:0035117 | embryonic arm morphogenesis |
| GO:0006461 | protein complex assembly |

- 1. GO process affected by Benzene

| **GO.ID** | **Term** |
| --- | --- |
| GO:0006917 | induction of apoptosis |
| GO:0016570 | histone modification |
| GO:0000288 | mRNA catabolic process, deadenylation-de... |
| GO:0006903 | vesicle targeting |
| GO:0001833 | inner cell mass cell proliferation |
| GO:0046148 | pigment biosynthetic process |
| GO:0030111 | regulation of Wnt receptor signaling pat... |
| GO:0006900 | membrane budding |
| GO:0000122 | negative regulation of transcription fro... |
| GO:0048193 | Golgi vesicle transport |
| GO:0007015 | actin filament organization |
| GO:0010332 | response to gamma radiation |
| GO:0033261 | regulation of S phase |
| GO:0006473 | protein amino acid acetylation |
| GO:0008535 | respiratory chain complex IV assembly |
| GO:0051291 | protein heterooligomerization |
| GO:0006413 | translational initiation |
| GO:0009411 | response to UV |
| GO:0006476 | protein amino acid deacetylation |
| GO:0030330 | DNA damage response, signal transduction... |
| GO:0045941 | positive regulation of transcription |
| GO:0019885 | antigen processing and presentation of e... |
| GO:0046834 | lipid phosphorylation |
| GO:0000072 | M phase specific microtubule process |
| GO:0000718 | nucleotide-excision repair, DNA damage r... |
| GO:0002326 | B cell lineage commitment |
| GO:0006450 | regulation of translational fidelity |
| GO:0006550 | isoleucine catabolic process |
| GO:0007503 | fat body development |
| GO:0009098 | leucine biosynthetic process |
| GO:0014049 | positive regulation of glutamate secreti... |
| GO:0019046 | reactivation of latent virus |
| GO:0031665 | negative regulation of lipopolysaccharid... |
| GO:0033169 | histone H3-K9 demethylation |
| GO:0033595 | response to genistein |
| GO:0033600 | negative regulation of mammary gland epi... |
| GO:0042488 | positive regulation of odontogenesis of ... |
| GO:0043461 | proton-transporting ATP synthase complex... |
| GO:0045368 | positive regulation of interleukin-13 bi... |
| GO:0046293 | formaldehyde biosynthetic process |
| GO:0046939 | nucleotide phosphorylation |
| GO:0000910 | cytokinesis |
| GO:0006012 | galactose metabolic process |
| GO:0006477 | protein amino acid sulfation |
| GO:0006559 | L-phenylalanine catabolic process |
| GO:0032367 | intracellular cholesterol transport |
| GO:0042147 | retrograde transport, endosome to Golgi |
| GO:0006879 | cellular iron ion homeostasis |
| GO:0043392 | negative regulation of DNA binding |
| GO:0051492 | regulation of stress fiber formation |

- 1. GO process affected by Carbontetrachloride

| **GO.ID** | **Term** |
| --- | --- |
| GO:0006412 | translation |
| GO:0051438 | regulation of ubiquitin-protein ligase a... |
| GO:0008615 | pyridoxine biosynthetic process |
| GO:0016574 | histone ubiquitination |
| GO:0006446 | regulation of translational initiation |
| GO:0009070 | serine family amino acid biosynthetic pr... |
| GO:0006919 | caspase activation |
| GO:0001711 | endodermal cell fate commitment |
| GO:0006450 | regulation of translational fidelity |
| GO:0006529 | asparagine biosynthetic process |
| GO:0030858 | positive regulation of epithelial cell d... |
| GO:0042789 | mRNA transcription from RNA polymerase I... |
| GO:0045204 | MAPK export from nucleus |
| GO:0045209 | MAPK phosphatase export from nucleus, le... |
| GO:0016071 | mRNA metabolic process |
| GO:0030968 | unfolded protein response |
| GO:0008380 | RNA splicing |
| GO:0007049 | cell cycle |
| GO:0000729 | DNA double-strand break processing |
| GO:0001706 | endoderm formation |
| GO:0002268 | follicular dendritic cell differentiatio... |
| GO:0006679 | glucosylceramide biosynthetic process |
| GO:0015788 | UDP-N-acetylglucosamine transport |
| GO:0019049 | evasion of host defenses by virus |
| GO:0031058 | positive regulation of histone modificat... |
| GO:0032792 | inhibition of CREB transcription factor |
| GO:0008219 | cell death |
| GO:0006605 | protein targeting |
| GO:0042542 | response to hydrogen peroxide |
| GO:0007249 | I-kappaB kinase/NF-kappaB cascade |
| GO:0006419 | alanyl-tRNA aminoacylation |
| GO:0015805 | S-adenosylmethionine transport |
| GO:0015909 | long-chain fatty acid transport |
| GO:0032331 | negative regulation of chondrocyte diffe... |
| GO:0042149 | cellular response to glucose starvation |
| GO:0043623 | cellular protein complex assembly |
| GO:0001892 | embryonic placenta development |
| GO:0001975 | response to amphetamine |
| GO:0006047 | UDP-N-acetylglucosamine metabolic proces... |
| GO:0006434 | seryl-tRNA aminoacylation |
| GO:0006926 | virus-infected cell apoptosis |
| GO:0035117 | embryonic arm morphogenesis |
| GO:0045739 | positive regulation of DNA repair |
| GO:0046686 | response to cadmium ion |
| GO:0048536 | spleen development |
| GO:0006406 | mRNA export from nucleus |

- 1. GO process affected by Cyclophosphamide

| **GO.ID** | **Term** |
| --- | --- |
| GO:0045908 | negative regulation of vasodilation |
| GO:0006468 | protein amino acid phosphorylation |
| GO:0048193 | Golgi vesicle transport |
| GO:0019896 | axon transport of mitochondrion |
| GO:0051000 | positive regulation of nitric-oxide synt... |
| GO:0045045 | secretory pathway |
| GO:0000266 | mitochondrial fission |
| GO:0001958 | endochondral ossification |
| GO:0008053 | mitochondrial fusion |
| GO:0016925 | protein sumoylation |
| GO:0050823 | peptide antigen stabilization |
| GO:0007259 | JAK-STAT cascade |
| GO:0001833 | inner cell mass cell proliferation |
| GO:0045909 | positive regulation of vasodilation |
| GO:0042177 | negative regulation of protein catabolic... |
| GO:0042531 | positive regulation of tyrosine phosphor... |
| GO:0008380 | RNA splicing |
| GO:0001974 | blood vessel remodeling |
| GO:0002062 | chondrocyte differentiation |
| GO:0006895 | Golgi to endosome transport |
| GO:0007007 | inner mitochondrial membrane organizatio... |
| GO:0019885 | antigen processing and presentation of e... |

- 1. GO process affected by Epichlorohydrin

| **GO.ID** | **Term** |
| --- | --- |
| GO:0006984 | ER-nuclear signaling pathway |
| GO:0007220 | Notch receptor processing |
| GO:0045941 | positive regulation of transcription |
| GO:0001808 | negative regulation of type IV hypersens... |
| GO:0045060 | negative thymic T cell selection |
| GO:0009083 | branched chain family amino acid catabol... |
| GO:0006551 | leucine metabolic process |
| GO:0042149 | cellular response to glucose starvation |
| GO:0007173 | epidermal growth factor receptor signali... |
| GO:0006509 | membrane protein ectodomain proteolysis |
| GO:0042987 | amyloid precursor protein catabolic proc... |
| GO:0007050 | cell cycle arrest |
| GO:0001562 | response to protozoan |
| GO:0042535 | positive regulation of tumor necrosis fa... |
| GO:0007049 | cell cycle |
| GO:0000209 | protein polyubiquitination |
| GO:0031295 | T cell costimulation |
| GO:0046902 | regulation of mitochondrial membrane per... |
| GO:0046834 | lipid phosphorylation |
| GO:0006289 | nucleotide-excision repair |
| GO:0006270 | DNA replication initiation |
| GO:0000080 | G1 phase of mitotic cell cycle |
| GO:0009411 | response to UV |
| GO:0045786 | negative regulation of cell cycle |
| GO:0001666 | response to hypoxia |
| GO:0050688 | regulation of defense response to virus |
| GO:0006457 | protein folding |
| GO:0008286 | insulin receptor signaling pathway |
| GO:0045454 | cell redox homeostasis |
| GO:0008284 | positive regulation of cell proliferatio... |
| GO:0006405 | RNA export from nucleus |
| GO:0007492 | endoderm development |
| GO:0019885 | antigen processing and presentation of e... |
| GO:0000188 | inactivation of MAPK activity |
| GO:0050851 | antigen receptor-mediated signaling path... |
| GO:0006012 | galactose metabolic process |
| GO:0001711 | endodermal cell fate commitment |
| GO:0001922 | B-1 B cell homeostasis |
| GO:0001988 | positive regulation of heart rate in bar... |
| GO:0006450 | regulation of translational fidelity |
| GO:0006529 | asparagine biosynthetic process |
| GO:0010269 | response to selenium ion |
| GO:0014061 | regulation of norepinephrine secretion |
| GO:0018144 | RNA-protein covalent cross-linking |
| GO:0019408 | dolichol biosynthetic process |
| GO:0022617 | extracellular matrix disassembly |
| GO:0030858 | positive regulation of epithelial cell d... |
| GO:0031293 | membrane protein intracellular domain pr... |
| GO:0031581 | hemidesmosome assembly |
| GO:0033169 | histone H3-K9 demethylation |
| GO:0042789 | mRNA transcription from RNA polymerase I... |
| GO:0043461 | proton-transporting ATP synthase complex... |
| GO:0045023 | G0 to G1 transition |
| GO:0045204 | MAPK export from nucleus |
| GO:0045209 | MAPK phosphatase export from nucleus, le... |
| GO:0045368 | positive regulation of interleukin-13 bi... |
| GO:0046293 | formaldehyde biosynthetic process |
| GO:0048149 | behavioral response to ethanol |
| GO:0051097 | negative regulation of helicase activity |
| GO:0060112 | generation of ovulation cycle rhythm |
| GO:0042130 | negative regulation of T cell proliferat... |
| GO:0051492 | regulation of stress fiber formation |
| GO:0045792 | negative regulation of cell size |
| GO:0031647 | regulation of protein stability |
| GO:0042981 | regulation of apoptosis |
| GO:0001836 | release of cytochrome c from mitochondri... |
| GO:0006378 | mRNA polyadenylation |
| GO:0042417 | dopamine metabolic process |
| GO:0048168 | regulation of neuronal synaptic plastici... |
| GO:0006423 | cysteinyl-tRNA aminoacylation |
| GO:0006565 | L-serine catabolic process |
| GO:0006597 | spermine biosynthetic process |
| GO:0007023 | post-chaperonin tubulin folding pathway |
| GO:0007527 | adult somatic muscle development |
| GO:0014010 | Schwann cell proliferation |
| GO:0015785 | UDP-galactose transport |
| GO:0015827 | tryptophan transport |
| GO:0019049 | evasion of host defenses by virus |
| GO:0032007 | negative regulation of TOR signaling pat... |
| GO:0032225 | regulation of synaptic transmission, dop... |
| GO:0032720 | negative regulation of tumor necrosis fa... |
| GO:0032792 | inhibition of CREB transcription factor |
| GO:0033599 | regulation of mammary gland epithelial c... |
| GO:0035090 | maintenance of apical/basal cell polarit... |
| GO:0042524 | negative regulation of tyrosine phosphor... |
| GO:0045082 | positive regulation of interleukin-10 bi... |
| GO:0046080 | dUTP metabolic process |
| GO:0046653 | tetrahydrofolate metabolic process |
| GO:0051894 | positive regulation of focal adhesion fo... |

- 1. GO process affected by Trichloroethylene

| **GO.ID** | **Term** |
| --- | --- |
| GO:0012501 | programmed cell death |
| GO:0006419 | alanyl-tRNA aminoacylation |
| GO:0006470 | protein amino acid dephosphorylation |
| GO:0007183 | SMAD protein complex assembly |
| GO:0007220 | Notch receptor processing |
| GO:0031295 | T cell costimulation |
| GO:0008380 | RNA splicing |
| GO:0007249 | I-kappaB kinase/NF-kappaB cascade |
| GO:0019885 | antigen processing and presentation of e... |
| GO:0002062 | chondrocyte differentiation |
| GO:0016458 | gene silencing |
| GO:0042993 | positive regulation of transcription fac... |
| GO:0008624 | induction of apoptosis by extracellular ... |
| GO:0006509 | membrane protein ectodomain proteolysis |
| GO:0042987 | amyloid precursor protein catabolic proc... |
| GO:0009615 | response to virus |
| GO:0000122 | negative regulation of transcription fro... |
| GO:0051252 | regulation of RNA metabolic process |
| GO:0001711 | endodermal cell fate commitment |
| GO:0006450 | regulation of translational fidelity |
| GO:0006529 | asparagine biosynthetic process |
| GO:0030858 | positive regulation of epithelial cell d... |
| GO:0031293 | membrane protein intracellular domain pr... |
| GO:0042789 | mRNA transcription from RNA polymerase I... |
| GO:0045023 | G0 to G1 transition |
| GO:0045204 | MAPK export from nucleus |
| GO:0045209 | MAPK phosphatase export from nucleus, le... |
| GO:0006919 | caspase activation |
| GO:0030968 | unfolded protein response |
| GO:0006401 | RNA catabolic process |
| GO:0001706 | endoderm formation |
| GO:0001808 | negative regulation of type IV hypersens... |
| GO:0006679 | glucosylceramide biosynthetic process |
| GO:0019049 | evasion of host defenses by virus |
| GO:0032792 | inhibition of CREB transcription factor |
| GO:0045060 | negative thymic T cell selection |
| GO:0043687 | post-translational protein modification |
| GO:0017015 | regulation of transforming growth factor... |
| GO:0001958 | endochondral ossification |
| GO:0016925 | protein sumoylation |
| GO:0019344 | cysteine biosynthetic process |
| GO:0032331 | negative regulation of chondrocyte diffe... |
| GO:0042149 | cellular response to glucose starvation |
| GO:0046967 | cytosol to ER transport |
| GO:0050823 | peptide antigen stabilization |
| GO:0007264 | small GTPase mediated signal transductio... |
| GO:0045941 | positive regulation of transcription |
| GO:0007568 | aging |
| GO:0000060 | protein import into nucleus, translocati... |
| GO:0001892 | embryonic placenta development |
| GO:0001975 | response to amphetamine |
| GO:0006654 | phosphatidic acid biosynthetic process |
| GO:0016246 | RNA interference |
| GO:0035117 | embryonic arm morphogenesis |
| GO:0046686 | response to cadmium ion |
| GO:0031647 | regulation of protein stability |

1. GO processes affected at high dose
   1. GO process affected by Acrylamide

| **GO.ID** | **Term** |
| --- | --- |
| GO:0030503 | regulation of cell redox homeostasis |
| GO:0042542 | response to hydrogen peroxide |
| GO:0007568 | aging |
| GO:0006954 | inflammatory response |
| GO:0010269 | response to selenium ion |
| GO:0042789 | mRNA transcription from RNA polymerase I... |
| GO:0045204 | MAPK export from nucleus |
| GO:0045209 | MAPK phosphatase export from nucleus, le... |
| GO:0007050 | cell cycle arrest |
| GO:0019049 | evasion of host defenses by virus |
| GO:0032792 | inhibition of CREB transcription factor |
| GO:0033599 | regulation of mammary gland epithelial c... |
| GO:0009968 | negative regulation of signal transducti... |
| GO:0043065 | positive regulation of apoptosis |
| GO:0009440 | cyanate catabolic process |
| GO:0019344 | cysteine biosynthetic process |
| GO:0045730 | respiratory burst |
| GO:0046967 | cytosol to ER transport |
| GO:0051302 | regulation of cell division |
| GO:0008219 | cell death |
| GO:0006470 | protein amino acid dephosphorylation |
| GO:0040029 | regulation of gene expression, epigeneti... |
| GO:0001892 | embryonic placenta development |
| GO:0001975 | response to amphetamine |
| GO:0046686 | response to cadmium ion |
| GO:0006865 | amino acid transport |
| GO:0007183 | SMAD protein complex assembly |
| GO:0008631 | induction of apoptosis by oxidative stre... |
| GO:0032909 | regulation of transforming growth factor... |
| GO:0006916 | anti-apoptosis |
| GO:0045935 | positive regulation of nucleobase, nucle... |
| GO:0006469 | negative regulation of protein kinase ac... |
| GO:0006983 | ER overload response |
| GO:0009650 | UV protection |
| GO:0006510 | ATP-dependent proteolysis |
| GO:0006857 | oligopeptide transport |
| GO:0019885 | antigen processing and presentation of e... |
| GO:0043154 | negative regulation of caspase activity |
| GO:0042993 | positive regulation of transcription fac... |
| GO:0033344 | cholesterol efflux |
| GO:0045408 | regulation of interleukin-6 biosynthetic... |
| GO:0001836 | release of cytochrome c from mitochondri... |
| GO:0019363 | pyridine nucleotide biosynthetic process |
| GO:0045930 | negative regulation of mitotic cell cycl... |
| GO:0006783 | heme biosynthetic process |
| GO:0008206 | bile acid metabolic process |
| GO:0006955 | immune response |
| GO:0043405 | regulation of MAP kinase activity |
| GO:0006950 | response to stress |
| GO:0007041 | lysosomal transport |
| GO:0030968 | unfolded protein response |

- 1. GO process affected by Benz[a]anthracene

| **GO.ID** | **Term** |
| --- | --- |
| GO:0045471 | response to ethanol |
| GO:0006863 | purine transport |
| GO:0019062 | virion attachment to host cell surface r... |
| GO:0033599 | regulation of mammary gland epithelial c... |
| GO:0006749 | glutathione metabolic process |
| GO:0042542 | response to hydrogen peroxide |
| GO:0007569 | cell aging |
| GO:0032272 | negative regulation of protein polymeriz... |
| GO:0016071 | mRNA metabolic process |
| GO:0008380 | RNA splicing |
| GO:0006188 | IMP biosynthetic process |
| GO:0019079 | viral genome replication |
| GO:0006563 | L-serine metabolic process |
| GO:0006007 | glucose catabolic process |
| GO:0032981 | mitochondrial respiratory chain complex ... |
| GO:0048678 | response to axon injury |
| GO:0006401 | RNA catabolic process |
| GO:0007059 | chromosome segregation |
| GO:0042311 | vasodilation |
| GO:0010038 | response to metal ion |
| GO:0006596 | polyamine biosynthetic process |
| GO:0030104 | water homeostasis |
| GO:0048538 | thymus development |
| GO:0000028 | ribosomal small subunit assembly and mai... |
| GO:0001711 | endodermal cell fate commitment |
| GO:0006231 | dTMP biosynthetic process |
| GO:0006450 | regulation of translational fidelity |
| GO:0006713 | glucocorticoid catabolic process |
| GO:0006789 | bilirubin conjugation |
| GO:0007400 | neuroblast fate determination |
| GO:0015855 | pyrimidine transport |
| GO:0018879 | biphenyl metabolic process |
| GO:0019322 | pentose biosynthetic process |
| GO:0019510 | S-adenosylhomocysteine catabolic process |
| GO:0030858 | positive regulation of epithelial cell d... |
| GO:0032287 | myelin maintenance in the peripheral ner... |
| GO:0033081 | regulation of T cell differentiation in ... |
| GO:0033595 | response to genistein |
| GO:0043128 | positive regulation of 1-phosphatidylino... |
| GO:0045199 | maintenance of epithelial cell polarity |
| GO:0045870 | positive regulation of retroviral genome... |
| GO:0045919 | positive regulation of cytolysis |
| GO:0046795 | intracellular virion transport |
| GO:0046968 | peptide antigen transport |
| GO:0060163 | subpallium neuron fate commitment |
| GO:0060165 | regulation of timing of subpallium neuro... |
| GO:0030262 | apoptotic nuclear changes |
| GO:0000187 | activation of MAPK activity |
| GO:0030503 | regulation of cell redox homeostasis |
| GO:0008217 | regulation of blood pressure |
| GO:0001837 | epithelial to mesenchymal transition |

- 1. GO process affected by Benzene

| **GO.ID** | **Term** |
| --- | --- |
| GO:0045935 | positive regulation of nucleobase, nucle... |
| GO:0006470 | protein amino acid dephosphorylation |
| GO:0043065 | positive regulation of apoptosis |
| GO:0046649 | lymphocyte activation |
| GO:0016575 | histone deacetylation |
| GO:0043280 | positive regulation of caspase activity |
| GO:0006783 | heme biosynthetic process |
| GO:0007528 | neuromuscular junction development |
| GO:0006529 | asparagine biosynthetic process |
| GO:0042789 | mRNA transcription from RNA polymerase I... |
| GO:0043461 | proton-transporting ATP synthase complex... |
| GO:0045204 | MAPK export from nucleus |
| GO:0045209 | MAPK phosphatase export from nucleus, le... |
| GO:0048568 | embryonic organ development |
| GO:0006780 | uroporphyrinogen III biosynthetic proces... |
| GO:0007527 | adult somatic muscle development |
| GO:0019049 | evasion of host defenses by virus |
| GO:0046653 | tetrahydrofolate metabolic process |
| GO:0000188 | inactivation of MAPK activity |
| GO:0012501 | programmed cell death |
| GO:0006954 | inflammatory response |
| GO:0000022 | mitotic spindle elongation |
| GO:0009440 | cyanate catabolic process |
| GO:0019344 | cysteine biosynthetic process |
| GO:0042149 | cellular response to glucose starvation |
| GO:0045425 | positive regulation of granulocyte macro... |
| GO:0045627 | positive regulation of T-helper 1 cell d... |
| GO:0045730 | respiratory burst |
| GO:0050823 | peptide antigen stabilization |
| GO:0051302 | regulation of cell division |
| GO:0001892 | embryonic placenta development |
| GO:0001975 | response to amphetamine |
| GO:0007518 | myoblast cell fate determination |
| GO:0046686 | response to cadmium ion |
| GO:0006122 | mitochondrial electron transport, ubiqui... |
| GO:0007183 | SMAD protein complex assembly |
| GO:0032909 | regulation of transforming growth factor... |
| GO:0033209 | tumor necrosis factor-mediated signaling... |
| GO:0042921 | glucocorticoid receptor signaling pathwa... |
| GO:0006983 | ER overload response |
| GO:0042347 | negative regulation of NF-kappaB import ... |

- 1. GO process affected by Ethylenedibromide

| **GO.ID** | **Term** |
| --- | --- |
| GO:0006166 | purine ribonucleoside salvage |
| GO:0008631 | induction of apoptosis by oxidative stre... |
| GO:0006168 | adenine salvage |
| GO:0006627 | mitochondrial protein processing during ... |
| GO:0015827 | tryptophan transport |
| GO:0006477 | protein amino acid sulfation |
| GO:0007625 | grooming behavior |
| GO:0008285 | negative regulation of cell proliferatio... |
| GO:0008625 | induction of apoptosis via death domain ... |
| GO:0046718 | entry of virus into host cell |
| GO:0031424 | keratinization |
| GO:0042100 | B cell proliferation |
| GO:0009411 | response to UV |
| GO:0007050 | cell cycle arrest |
| GO:0006729 | tetrahydrobiopterin biosynthetic process |
| GO:0042311 | vasodilation |
| GO:0006919 | caspase activation |
| GO:0045776 | negative regulation of blood pressure |
| GO:0006559 | L-phenylalanine catabolic process |
| GO:0007403 | glial cell fate determination |
| GO:0008049 | male courtship behavior |
| GO:0009229 | thiamin diphosphate biosynthetic process |
| GO:0009399 | nitrogen fixation |
| GO:0010269 | response to selenium ion |
| GO:0015961 | diadenosine polyphosphate catabolic proc... |
| GO:0018146 | keratan sulfate biosynthetic process |
| GO:0018350 | protein amino acid esterification |
| GO:0019068 | virus assembly |
| GO:0030828 | positive regulation of cGMP biosynthetic... |
| GO:0030889 | negative regulation of B cell proliferat... |
| GO:0042789 | mRNA transcription from RNA polymerase I... |
| GO:0043071 | positive regulation of non-apoptotic pro... |
| GO:0044258 | intestinal lipid catabolic process |
| GO:0046597 | negative regulation of virion penetratio... |
| GO:0046963 | 3'-phosphoadenosine 5'-phosphosulfate tr... |
| GO:0051044 | positive regulation of membrane protein ... |
| GO:0065004 | protein-DNA complex assembly |
| GO:0006707 | cholesterol catabolic process |
| GO:0030503 | regulation of cell redox homeostasis |
| GO:0042551 | neuron maturation |
| GO:0006418 | tRNA aminoacylation for protein translat... |
| GO:0000302 | response to reactive oxygen species |
| GO:0007339 | binding of sperm to zona pellucida |
| GO:0016601 | Rac protein signal transduction |
| GO:0006916 | anti-apoptosis |
| GO:0001658 | ureteric bud branching |
| GO:0006228 | UTP biosynthetic process |
| GO:0030195 | negative regulation of blood coagulation |
| GO:0006241 | CTP biosynthetic process |
| GO:0015671 | oxygen transport |
| GO:0001835 | blastocyst hatching |
| GO:0006222 | UMP biosynthetic process |
| GO:0006346 | methylation-dependent chromatin silencin... |
| GO:0006537 | glutamate biosynthetic process |
| GO:0007506 | gonadal mesoderm development |
| GO:0009313 | oligosaccharide catabolic process |
| GO:0015785 | UDP-galactose transport |
| GO:0016075 | rRNA catabolic process |
| GO:0016556 | mRNA modification |
| GO:0019673 | GDP-mannose metabolic process |
| GO:0030187 | melatonin biosynthetic process |
| GO:0032582 | negative regulation of gene-specific tra... |
| GO:0032792 | inhibition of CREB transcription factor |
| GO:0033599 | regulation of mammary gland epithelial c... |
| GO:0042351 | 'de novo' GDP-L-fucose biosynthetic proc... |
| GO:0043537 | negative regulation of blood vessel endo... |
| GO:0045343 | regulation of MHC class I biosynthetic p... |
| GO:0046475 | glycerophospholipid catabolic process |
| GO:0048711 | positive regulation of astrocyte differe... |
| GO:0051000 | positive regulation of nitric-oxide synt... |
| GO:0060056 | mammary gland involution |

- 1. GO process affected by Epichlorohydrin

| **GO.ID** | **Term** |
| --- | --- |
| GO:0007163 | establishment and/or maintenance of cell... |
| GO:0031295 | T cell costimulation |
| GO:0001808 | negative regulation of type IV hypersens... |
| GO:0045060 | negative thymic T cell selection |
| GO:0051043 | regulation of membrane protein ectodomai... |
| GO:0007050 | cell cycle arrest |
| GO:0006397 | mRNA processing |
| GO:0016246 | RNA interference |
| GO:0043065 | positive regulation of apoptosis |
| GO:0001562 | response to protozoan |
| GO:0032024 | positive regulation of insulin secretion |
| GO:0042535 | positive regulation of tumor necrosis fa... |
| GO:0043249 | erythrocyte maturation |
| GO:0006986 | response to unfolded protein |
| GO:0031274 | positive regulation of pseudopodium form... |
| GO:0035116 | embryonic hindlimb morphogenesis |
| GO:0046836 | glycolipid transport |
| GO:0060070 | Wnt receptor signaling pathway through b... |
| GO:0046834 | lipid phosphorylation |
| GO:0006953 | acute-phase response |
| GO:0050688 | regulation of defense response to virus |
| GO:0001711 | endodermal cell fate commitment |
| GO:0006450 | regulation of translational fidelity |
| GO:0006789 | bilirubin conjugation |
| GO:0006851 | mitochondrial calcium ion transport |
| GO:0007403 | glial cell fate determination |
| GO:0007443 | Malpighian tubule morphogenesis |
| GO:0008356 | asymmetric cell division |
| GO:0008611 | ether lipid biosynthetic process |
| GO:0015855 | pyrimidine transport |
| GO:0018879 | biphenyl metabolic process |
| GO:0019747 | regulation of isoprenoid metabolic proce... |
| GO:0030046 | parallel actin filament bundle formation |
| GO:0030828 | positive regulation of cGMP biosynthetic... |
| GO:0030858 | positive regulation of epithelial cell d... |
| GO:0042538 | hyperosmotic salinity response |
| GO:0042789 | mRNA transcription from RNA polymerase I... |
| GO:0043071 | positive regulation of non-apoptotic pro... |
| GO:0048312 | intracellular distribution of mitochondr... |
| GO:0051988 | regulation of attachment of spindle micr... |
| GO:0006688 | glycosphingolipid biosynthetic process |
| GO:0007263 | nitric oxide mediated signal transductio... |
| GO:0006968 | cellular defense response |
| GO:0006099 | tricarboxylic acid cycle |
| GO:0006878 | cellular copper ion homeostasis |
| GO:0009070 | serine family amino acid biosynthetic pr... |
| GO:0042130 | negative regulation of T cell proliferat... |
| GO:0045471 | response to ethanol |
| GO:0031047 | RNA-mediated gene silencing |
| GO:0045736 | negative regulation of cyclin-dependent ... |
| GO:0006446 | regulation of translational initiation |
| GO:0001755 | neural crest cell migration |
| GO:0006825 | copper ion transport |
| GO:0031532 | actin cytoskeleton reorganization |
| GO:0048168 | regulation of neuronal synaptic plastici... |

- 1. GO process affected by Hydroquinone

| GO.ID | Term |
| --- | --- |
| GO:0001808 | negative regulation of type IV hypersens... |
| GO:0045060 | negative thymic T cell selection |
| GO:0007163 | establishment and/or maintenance of cell... |
| GO:0008624 | induction of apoptosis by extracellular ... |
| GO:0001562 | response to protozoan |
| GO:0042535 | positive regulation of tumor necrosis fa... |
| GO:0007220 | Notch receptor processing |
| GO:0031295 | T cell costimulation |
| GO:0050688 | regulation of defense response to virus |
| GO:0006098 | pentose-phosphate shunt |
| GO:0019885 | antigen processing and presentation of e... |
| GO:0002062 | chondrocyte differentiation |
| GO:0007050 | cell cycle arrest |
| GO:0042130 | negative regulation of T cell proliferat... |
| GO:0006509 | membrane protein ectodomain proteolysis |
| GO:0042987 | amyloid precursor protein catabolic proc... |
| GO:0045941 | positive regulation of transcription |
| GO:0006890 | retrograde vesicle-mediated transport, G... |
| GO:0001711 | endodermal cell fate commitment |
| GO:0006010 | glucose 6-phosphate utilization |
| GO:0006450 | regulation of translational fidelity |
| GO:0015961 | diadenosine polyphosphate catabolic proc... |
| GO:0018350 | protein amino acid esterification |
| GO:0019322 | pentose biosynthetic process |
| GO:0019408 | dolichol biosynthetic process |
| GO:0030858 | positive regulation of epithelial cell d... |
| GO:0031293 | membrane protein intracellular domain pr... |
| GO:0042789 | mRNA transcription from RNA polymerase I... |
| GO:0044258 | intestinal lipid catabolic process |
| GO:0006366 | transcription from RNA polymerase II pro... |
| GO:0030968 | unfolded protein response |
| GO:0006968 | cellular defense response |
| GO:0000059 | protein import into nucleus, docking |
| GO:0016485 | protein processing |
| GO:0001706 | endoderm formation |
| GO:0002268 | follicular dendritic cell differentiatio... |
| GO:0006679 | glucosylceramide biosynthetic process |
| GO:0032792 | inhibition of CREB transcription factor |
| GO:0046022 | positive regulation of transcription fro... |
| GO:0009615 | response to virus |
| GO:0006355 | regulation of transcription, DNA-depende... |
| GO:0000184 | mRNA catabolic process, nonsense-mediate... |
| GO:0042102 | positive regulation of T cell proliferat... |
| GO:0007568 | aging |
| GO:0001958 | endochondral ossification |
| GO:0006880 | intracellular sequestering of iron ion |
| GO:0030157 | pancreatic juice secretion |
| GO:0032331 | negative regulation of chondrocyte diffe... |
| GO:0043011 | myeloid dendritic cell differentiation |
| GO:0046967 | cytosol to ER transport |
| GO:0050823 | peptide antigen stabilization |
| GO:0000060 | protein import into nucleus, translocati... |
| GO:0001892 | embryonic placenta development |
| GO:0001975 | response to amphetamine |
| GO:0016246 | RNA interference |
| GO:0035117 | embryonic arm morphogenesis |
| GO:0048536 | spleen development |
| GO:0006406 | mRNA export from nucleus |
| GO:0030100 | regulation of endocytosis |
| GO:0006955 | immune response |

- 1. GO process affected by Styrene

| **GO.ID** | **Term** |
| --- | --- |
| GO:0032792 | inhibition of CREB transcription factor |
| GO:0019344 | cysteine biosynthetic process |
| GO:0001975 | response to amphetamine |
| GO:0006983 | ER overload response |
| GO:0043065 | positive regulation of apoptosis |
| GO:0030503 | regulation of cell redox homeostasis |
| GO:0019363 | pyridine nucleotide biosynthetic process |
| GO:0030968 | unfolded protein response |
| GO:0048568 | embryonic organ development |
| GO:0042542 | response to hydrogen peroxide |
| GO:0007568 | aging |
| GO:0042127 | regulation of cell proliferation |
| GO:0007584 | response to nutrient |
| GO:0006955 | immune response |
| GO:0009605 | response to external stimulus |
| GO:0007267 | cell-cell signaling |
| GO:0031667 | response to nutrient levels |
| GO:0009991 | response to extracellular stimulus |
| GO:0042742 | defense response to bacterium |
| GO:0008283 | cell proliferation |
| GO:0009617 | response to bacterium |
| GO:0002376 | immune system process |
| GO:0007050 | cell cycle arrest |
| GO:0006935 | chemotaxis |
| GO:0007626 | locomotory behavior |
| GO:0009607 | response to biotic stimulus |
| GO:0006917 | induction of apoptosis |
| GO:0012502 | induction of programmed cell death |
| GO:0008284 | positive regulation of cell proliferatio... |
| GO:0008285 | negative regulation of cell proliferatio... |
| GO:0045941 | positive regulation of transcription |
| GO:0007610 | behavior |
| GO:0006954 | inflammatory response |
| GO:0006974 | response to DNA damage stimulus |
| GO:0031325 | positive regulation of cellular metaboli... |
| GO:0009719 | response to endogenous stimulus |
| GO:0009893 | positive regulation of metabolic process |
| GO:0009611 | response to wounding |
| GO:0022402 | cell cycle process |
| GO:0006952 | defense response |
| GO:0007049 | cell cycle |
| GO:0006950 | response to stress |
| GO:0007165 | signal transduction |
| GO:0007154 | cell communication |

- 1. GO process affected by Styrene 7,8-oxide

| **GO.ID** | **Term** |
| --- | --- |
| GO:0001504 | neurotransmitter uptake |
| GO:0006863 | purine transport |
| GO:0042311 | vasodilation |
| GO:0006461 | protein complex assembly |
| GO:0045494 | photoreceptor cell maintenance |
| GO:0045745 | positive regulation of G-protein coupled... |
| GO:0006662 | glycerol ether metabolic process |
| GO:0042158 | lipoprotein biosynthetic process |
| GO:0048193 | Golgi vesicle transport |
| GO:0043691 | reverse cholesterol transport |
| GO:0006839 | mitochondrial transport |
| GO:0030104 | water homeostasis |
| GO:0030162 | regulation of proteolysis |
| GO:0007254 | JNK cascade |
| GO:0048167 | regulation of synaptic plasticity |
| GO:0043066 | negative regulation of apoptosis |
| GO:0048538 | thymus development |
| GO:0050885 | neuromuscular process controlling balanc... |
| GO:0000028 | ribosomal small subunit assembly and mai... |
| GO:0000114 | G1-specific transcription in mitotic cel... |
| GO:0006649 | phospholipid transfer to membrane |
| GO:0006713 | glucocorticoid catabolic process |
| GO:0006837 | serotonin transport |
| GO:0007196 | metabotropic glutamate receptor, adenyla... |
| GO:0007400 | neuroblast fate determination |
| GO:0008611 | ether lipid biosynthetic process |
| GO:0010248 | establishment and/or maintenance of tran... |
| GO:0015855 | pyrimidine transport |
| GO:0030046 | parallel actin filament bundle formation |
| GO:0030828 | positive regulation of cGMP biosynthetic... |
| GO:0032235 | negative regulation of calcium ion trans... |
| GO:0045199 | maintenance of epithelial cell polarity |
| GO:0050748 | negative regulation of lipoprotein metab... |
| GO:0051044 | positive regulation of membrane protein ... |
| GO:0051988 | regulation of attachment of spindle micr... |
| GO:0055009 | atrial cardiac muscle morphogenesis |
| GO:0060163 | subpallium neuron fate commitment |
| GO:0060165 | regulation of timing of subpallium neuro... |
| GO:0030049 | muscle filament sliding |
| GO:0016570 | histone modification |
| GO:0016558 | protein import into peroxisome matrix |
| GO:0042472 | inner ear morphogenesis |
| GO:0043523 | regulation of neuron apoptosis |
| GO:0030308 | negative regulation of cell growth |
| GO:0007611 | learning and/or memory |
| GO:0001937 | negative regulation of endothelial cell ... |
| GO:0006637 | acyl-CoA metabolic process |

- 1. GO process affected by Trichloroethylene

| GO.ID | Term |
| --- | --- |
| GO:0030036 | actin cytoskeleton organization and biog... |
| GO:0006166 | purine ribonucleoside salvage |
| GO:0031295 | T cell costimulation |
| GO:0000398 | nuclear mRNA splicing, via spliceosome |
| GO:0001808 | negative regulation of type IV hypersens... |
| GO:0045060 | negative thymic T cell selection |
| GO:0045226 | extracellular polysaccharide biosyntheti... |
| GO:0051451 | myoblast migration |
| GO:0006556 | S-adenosylmethionine biosynthetic proces... |
| GO:0007163 | establishment and/or maintenance of cell... |
| GO:0045941 | positive regulation of transcription |
| GO:0035022 | positive regulation of Rac protein signa... |
| GO:0030521 | androgen receptor signaling pathway |
| GO:0001562 | response to protozoan |
| GO:0007183 | SMAD protein complex assembly |
| GO:0016071 | mRNA metabolic process |
| GO:0006487 | protein amino acid N-linked glycosylatio... |
| GO:0007220 | Notch receptor processing |
| GO:0045624 | positive regulation of T-helper cell dif... |
| GO:0006512 | ubiquitin cycle |
| GO:0006621 | protein retention in ER |
| GO:0009113 | purine base biosynthetic process |
| GO:0030308 | negative regulation of cell growth |
| GO:0000080 | G1 phase of mitotic cell cycle |
| GO:0008624 | induction of apoptosis by extracellular ... |
| GO:0016601 | Rac protein signal transduction |
| GO:0006596 | polyamine biosynthetic process |
| GO:0050688 | regulation of defense response to virus |
| GO:0045892 | negative regulation of transcription, DN... |
| GO:0008380 | RNA splicing |
| GO:0006857 | oligopeptide transport |
| GO:0009071 | serine family amino acid catabolic proce... |
| GO:0019885 | antigen processing and presentation of e... |
| GO:0048538 | thymus development |
| GO:0000290 | deadenylation-dependent decapping |
| GO:0001711 | endodermal cell fate commitment |
| GO:0006450 | regulation of translational fidelity |
| GO:0015855 | pyrimidine transport |
| GO:0015961 | diadenosine polyphosphate catabolic proc... |
| GO:0018144 | RNA-protein covalent cross-linking |
| GO:0019408 | dolichol biosynthetic process |
| GO:0019510 | S-adenosylhomocysteine catabolic process |
| GO:0030858 | positive regulation of epithelial cell d... |
| GO:0031293 | membrane protein intracellular domain pr... |
| GO:0032417 | positive regulation of sodium:hydrogen a... |
| GO:0032469 | endoplasmic reticulum calcium ion homeos... |
| GO:0033962 | cytoplasmic mRNA processing body assembl... |
| GO:0034063 | stress granule assembly |
| GO:0045175 | basal protein localization |
| GO:0045657 | positive regulation of monocyte differen... |
| GO:0051036 | regulation of endosome size |
| GO:0051097 | negative regulation of helicase activity |
| GO:0055009 | atrial cardiac muscle morphogenesis |
| GO:0050658 | RNA transport |
| GO:0000184 | mRNA catabolic process, nonsense-mediate... |
| GO:0030262 | apoptotic nuclear changes |
| GO:0042102 | positive regulation of T cell proliferat... |
| GO:0045944 | positive regulation of transcription fro... |
| GO:0042130 | negative regulation of T cell proliferat... |
| GO:0048704 | embryonic skeletal morphogenesis |
| GO:0006493 | protein amino acid O-linked glycosylatio... |
| GO:0006506 | GPI anchor biosynthetic process |
| GO:0051016 | barbed-end actin filament capping |
| GO:0006968 | cellular defense response |
| GO:0006916 | anti-apoptosis |
| GO:0001837 | epithelial to mesenchymal transition |
| GO:0006509 | membrane protein ectodomain proteolysis |
| GO:0007569 | cell aging |
| GO:0042987 | amyloid precursor protein catabolic proc... |
