# Supplementary material for: Effect of Chemical Mutagens and Carcinogens on Gene Expression Profiles in Human TK6 Cells
Source: PLoS One. 2012 Jun 18;7(6):e39205. doi: 10.1371/journal.pone.0039205 (PMC3377624; doi:10.1371/journal.pone.0039205)
Supplement: Table S3 — Functional classification of significantly impacted genes by exposure to carcinogens (S9+) at low, medium and high dose into gene ontology (GO) categories. (DOC) [file pone.0039205.s003.doc]

**Supplementary Table S3: Functional classification of significantly impacted genes by exposure to carcinogens (S9+) at low, medium and high dose into gene ontology (GO) categories.**

1. GO processes affected at low dose
   - 1. GO processes affected by Acrylamide

| **GO.ID** | **Term** |
| --- | --- |
| GO:0001808 | negative regulation of type IV hypersensitivity |
| GO:0045060 | negative thymic T cell selection |
| GO:0001562 | response to protozoan |
| GO:0042535 | positive regulation of tumor necrosis factor |
| GO:0031295 | T cell costimulation |
| GO:0050688 | regulation of defense response to virus |
| GO:0042130 | negative regulation of T cell proliferation |
| GO:0042102 | positive regulation of T cell proliferation |
| GO:0007162 | negative regulation of cell adhesion |
| GO:0007163 | establishment and/or maintenance of cell |
| GO:0008624 | induction of apoptosis by extracellular |
| GO:0042742 | defense response to bacterium |
| GO:0006968 | cellular defense response |
| GO:0042254 | ribosome biogenesis and assembly |
| GO:0006869 | lipid transport |
| GO:0006935 | chemotaxis |
| GO:0000122 | negative regulation of transcription fro. |
| GO:0012501 | programmed cell death |
| GO:0002376 | immune system process |
| GO:0006468 | protein amino acid phosphorylation |
| GO:0007275 | multicellular organismal development |
| GO:0007166 | cell surface receptor linked signal transduction |
| GO:0000902 | cell morphogenesis |
| GO:0001775 | cell activation |
| GO:0001806 | type IV hypersensitivity |
| GO:0001807 | regulation of type IV hypersensitivity |
| GO:0001816 | cytokine production |
| GO:0002250 | adaptive immune response |
| GO:0002252 | immune effector process |
| GO:0002437 | inflammatory response to antigenic stimulation |
| GO:0002438 | acute inflammatory response to antigenic |
| GO:0002443 | leukocyte mediated immunity |
| GO:0002449 | lymphocyte mediated immunity |
| GO:0002456 | T cell mediated immunity |
| GO:0002460 | adaptive immune response based |
| GO:0002520 | immune system development |
| GO:0002521 | leukocyte differentiation |
| GO:0002524 | hypersensitivity |
| GO:0002526 | acute inflammatory response |
| GO:0002673 | regulation of acute inflammatory response |
| GO:0002674 | negative regulation of acute inflammatory |
| GO:0002682 | regulation of immune system process |
| GO:0002683 | negative regulation of immune system process |
| GO:0002684 | positive regulation of immune system process |
| GO:0002694 | regulation of leukocyte activation |
| GO:0002695 | negative regulation of leukocyte activatation |
| GO:0002696 | positive regulation of leukocyte activation |
| GO:0002697 | regulation of immune effector process |
| GO:0002698 | negative regulation of immune effector |
| GO:0002703 | regulation of leukocyte mediated immunity |
| GO:0002704 | negative regulation of leukocyte mediate... |
| GO:0002706 | regulation of lymphocyte mediated immunity |
| GO:0002707 | negative regulation of lymphocyte |
| GO:0002709 | regulation of T cell mediated immunity |
| GO:0002710 | negative regulation of T cell mediated |
| GO:0002819 | regulation of adaptive immune response |
| GO:0002820 | negative regulation of adaptive immune |
| GO:0002822 | regulation of adaptive immune response |
| GO:0002823 | negative regulation of adaptive immune |
| GO:0002831 | regulation of response to biotic stimulus |
| GO:0002861 | regulation of inflammatory response |
| GO:0002862 | negative regulation of inflammatory response |
| GO:0002864 | regulation of acute inflammatory response |
| GO:0002865 | negative regulation of acute inflammatory |
| GO:0002883 | regulation of hypersensitivity |
| GO:0002884 | negative regulation of hypersensitivity |
| GO:0006139 | nucleobase, nucleoside, nucleotide |
| GO:0006350 | transcription |
| GO:0006351 | transcription, DNA-dependent |
| GO:0006355 | regulation of transcription, DNA-dependendent |
| GO:0006357 | regulation of transcription from RNA polymerase |
| GO:0006366 | transcription from RNA polymerase II |
| GO:0006412 | translation |
| GO:0006417 | regulation of translation |
| GO:0006464 | protein modification process |
| GO:0006793 | phosphorus metabolic process |
| GO:0006796 | phosphate metabolic process |
| GO:0006810 | transport |
| GO:0006915 | apoptosis |
| GO:0006917 | induction of apoptosis |
| GO:0006950 | response to stress |
| GO:0006952 | defense response |
| GO:0006954 | inflammatory response |
| GO:0006955 | immune response |
| GO:0006996 | organelle organization and biogenesis |
| GO:0007154 | cell communication |
| GO:0007155 | cell adhesion |
| GO:0007165 | signal transduction |
| GO:0007610 | behavior |
| GO:0007626 | locomotory behavior |
| GO:0008150 | biological_process |
| GO:0008152 | metabolic process |
| GO:0008219 | cell death |
| GO:0008283 | cell proliferation |
| GO:0008284 | positive regulation of cell proliferation |
| GO:0008285 | negative regulation of cell proliferation |
| GO:0009058 | biosynthetic process |
| GO:0009059 | macromolecule biosynthetic process |
| GO:0009605 | response to external stimulus |
| GO:0009607 | response to biotic stimulus |

- - 1. GO processes affected by Benzene

| **GO.ID** | **Term** |
| --- | --- |
| GO:0001808 | negative regulation of type IV hypersensitivity |
| GO:0045060 | negative thymic T cell selection |
| GO:0001562 | response to protozoan |
| GO:0042535 | positive regulation of tumor necrosis factor |
| GO:0031295 | T cell costimulation |
| GO:0046902 | regulation of mitochondrial membrane |
| GO:0050688 | regulation of defense response to virus |
| GO:0019885 | antigen processing and presentation |
| GO:0008624 | induction of apoptosis by extracellular |
| GO:0042130 | negative regulation of T cell proliferation |
| GO:0007569 | cell aging |
| GO:0001711 | endodermal cell fate commitment |
| GO:0002827 | positive regulation of T-helper 1 type |
| GO:0006450 | regulation of translational fidelity |
| GO:0018144 | RNA-protein covalent cross-linking |
| GO:0019408 | dolichol biosynthetic process |
| GO:0030858 | positive regulation of epithelial cell |
| GO:0032469 | endoplasmic reticulum calcium ion |
| GO:0045023 | G0 to G1 transition |
| GO:0051097 | negative regulation of helicase activity |
| GO:0007050 | cell cycle arrest |
| GO:0006801 | superoxide metabolic process |
| GO:0043687 | post-translational protein modification |
| GO:0050821 | protein stabilization |
| GO:0006461 | protein complex assembly |
| GO:0006916 | anti-apoptosis |
| GO:0001706 | endoderm formation |
| GO:0009648 | photoperiodism |
| GO:0019478 | D-amino acid catabolic process |
| GO:0046080 | dUTP metabolic process |
| GO:0042102 | positive regulation of T cell proliferation |
| GO:0006357 | regulation of transcription from RNA |
| GO:0006984 | ER-nuclear signaling pathway |
| GO:0051016 | barbed-end actin filament capping |
| GO:0006284 | base-excision repair |
| GO:0032331 | negative regulation of chondrocyte |
| GO:0042149 | cellular response to glucose starvation |
| GO:0046967 | cytosol to ER transport |
| GO:0050823 | peptide antigen stabilization |
| GO:0000398 | nuclear mRNA splicing, via spliceosome |
| GO:0042742 | defense response to bacterium |
| GO:0016310 | phosphorylation |
| GO:0012501 | programmed cell death |
| GO:0006289 | nucleotide-excision repair |
| GO:0002829 | negative regulation of T-helper 2 type |
| GO:0006654 | phosphatidic acid biosynthetic process |
| GO:0008634 | negative regulation of survival gene |
| GO:0035117 | embryonic arm morphogenesis |
| GO:0048302 | regulation of isotype switching to IgG |
| GO:0007162 | negative regulation of cell adhesion |
| GO:0007163 | establishment and/or maintenance of cell |
| GO:0006968 | cellular defense response |
| GO:0048193 | Golgi vesicle transport |
| GO:0009950 | dorsal/ventral axis specification |
| GO:0032729 | positive regulation of interferon-gamma |
| GO:0043249 | erythrocyte maturation |
| GO:0030521 | androgen receptor signaling pathway |
| GO:0002347 | response to tumor cell |
| GO:0007220 | Notch receptor processing |
| GO:0008635 | caspase activation via cytochrome c |
| GO:0009954 | proximal/distal pattern formation |
| GO:0035116 | embryonic hindlimb morphogenesis |
| GO:0042177 | negative regulation of protein catabolic |
| GO:0045669 | positive regulation of osteoblast differ... |
| GO:0060070 | Wnt receptor signaling pathway through |
| GO:0045786 | negative regulation of cell cycle |
| GO:0006621 | protein retention in ER |
| GO:0009103 | lipopolysaccharide biosynthetic process |
| GO:0045671 | negative regulation of osteoclast differentation |
| GO:0006596 | polyamine biosynthetic process |
| GO:0006777 | Mo-molybdopterin cofactor biosynthetic |
| GO:0050853 | B cell receptor signaling pathway |
| GO:0006857 | oligopeptide transport |
| GO:0042733 | embryonic digit morphogenesis |
| GO:0006096 | glycolysis |
| GO:0051881 | regulation of mitochondrial membrane |
| GO:0006491 | N-glycan processing |
| GO:0006903 | vesicle targeting |
| GO:0042771 | DNA damage response, signal transduction |
| GO:0001569 | patterning of blood vessels |
| GO:0001837 | epithelial to mesenchymal transition |
| GO:0006509 | membrane protein ectodomain proteolysis |
| GO:0030433 | ER-associated protein catabolic process |
| GO:0031016 | pancreas development |
| GO:0042987 | amyloid precursor protein catabolic proc... |
| GO:0001836 | release of cytochrome c from mitochondri |
| GO:0007059 | chromosome segregation |
| GO:0030308 | negative regulation of cell growth |
| GO:0048593 | camera-type eye morphogenesis |
| GO:0032943 | mononuclear cell proliferation |
| GO:0006783 | heme biosynthetic process |
| GO:0031032 | actomyosin structure organization and bi... |
| GO:0045453 | bone resorption |
| GO:0000209 | protein polyubiquitination |
| GO:0006000 | fructose metabolic process |
| GO:0006935 | chemotaxis |
| GO:0000059 | protein import into nucleus, docking |
| GO:0000080 | G1 phase of mitotic cell cycle |
| GO:0001708 | cell fate specification |
| GO:0042475 | odontogenesis of dentine-containing teet.. |

1. GO processes affected by Benz[a]anthracene

| **GO.ID** | **Term** |
| --- | --- |
| GO:0001808 | negative regulation of type IV hypersens... |
| GO:0045060 | negative thymic T cell selection |
| GO:0019885 | antigen processing and presentation of e... |
| GO:0007569 | cell aging |
| GO:0050823 | peptide antigen stabilization |
| GO:0006915 | apoptosis |
| GO:0001562 | response to protozoan |
| GO:0042535 | positive regulation of tumor necrosis fa... |
| GO:0031295 | T cell costimulation |
| GO:0046902 | regulation of mitochondrial membrane per... |
| GO:0043065 | positive regulation of apoptosis |
| GO:0006461 | protein complex assembly |
| GO:0051607 | defense response to virus |
| GO:0006596 | polyamine biosynthetic process |
| GO:0032989 | cellular structure morphogenesis |
| GO:0048193 | Golgi vesicle transport |
| GO:0042130 | negative regulation of T cell proliferat... |
| GO:0045786 | negative regulation of cell cycle |
| GO:0007050 | cell cycle arrest |
| GO:0001711 | endodermal cell fate commitment |
| GO:0001922 | B-1 B cell homeostasis |
| GO:0002827 | positive regulation of T-helper 1 type i... |
| GO:0006450 | regulation of translational fidelity |
| GO:0006843 | mitochondrial citrate transport |
| GO:0018144 | RNA-protein covalent cross-linking |
| GO:0019408 | dolichol biosynthetic process |
| GO:0030858 | positive regulation of epithelial cell d... |
| GO:0045023 | G0 to G1 transition |
| GO:0051036 | regulation of endosome size |
| GO:0051097 | negative regulation of helicase activity |
| GO:0008286 | insulin receptor signaling pathway |
| GO:0030521 | androgen receptor signaling pathway |
| GO:0006470 | protein amino acid dephosphorylation |
| GO:0006801 | superoxide metabolic process |
| GO:0006487 | protein amino acid N-linked glycosylatio... |
| GO:0001706 | endoderm formation |
| GO:0006552 | leucine catabolic process |
| GO:0010259 | multicellular organismal aging |
| GO:0016557 | peroxisome membrane biogenesis |
| GO:0019478 | D-amino acid catabolic process |
| GO:0030836 | positive regulation of actin filament de... |
| GO:0032581 | ER-dependent peroxisome biogenesis |
| GO:0032855 | positive regulation of Rac GTPase activi... |
| GO:0046080 | dUTP metabolic process |
| GO:0000059 | protein import into nucleus, docking |
| GO:0048489 | synaptic vesicle transport |
| GO:0050821 | protein stabilization |
| GO:0006968 | cellular defense response |
| GO:0006313 | transposition, DNA-mediated |
| GO:0006601 | creatine biosynthetic process |
| GO:0032331 | negative regulation of chondrocyte diffe... |
| GO:0040009 | regulation of growth rate |
| GO:0042149 | cellular response to glucose starvation |
| GO:0042420 | dopamine catabolic process |
| GO:0046967 | cytosol to ER transport |
| GO:0000188 | inactivation of MAPK activity |
| GO:0007016 | cytoskeletal anchoring |
| GO:0042102 | positive regulation of T cell proliferat... |
| GO:0050851 | antigen receptor-mediated signaling path... |
| GO:0051329 | interphase of mitotic cell cycle |
| GO:0000060 | protein import into nucleus, translocati... |
| GO:0001662 | behavioral fear response |
| GO:0001881 | receptor recycling |
| GO:0002829 | negative regulation of T-helper 2 type i... |
| GO:0006207 | 'de novo' pyrimidine base biosynthetic p... |
| GO:0006636 | unsaturated fatty acid biosynthetic proc... |
| GO:0006654 | phosphatidic acid biosynthetic process |
| GO:0008634 | negative regulation of survival gene pro... |
| GO:0035022 | positive regulation of Rac protein signa... |
| GO:0035117 | embryonic arm morphogenesis |
| GO:0045046 | protein import into peroxisome membrane |
| GO:0048302 | regulation of isotype switching to IgG i... |
| GO:0015986 | ATP synthesis coupled proton transport |
| GO:0006284 | base-excision repair |
| GO:0022613 | ribonucleoprotein complex biogenesis and... |
| GO:0002726 | positive regulation of T cell cytokine p... |
| GO:0006390 | transcription from mitochondrial promote... |
| GO:0009950 | dorsal/ventral axis specification |
| GO:0016255 | attachment of GPI anchor to protein |
| GO:0032729 | positive regulation of interferon-gamma ... |
| GO:0032743 | positive regulation of interleukin-2 pro... |
| GO:0035249 | synaptic transmission, glutamatergic |
| GO:0042518 | negative regulation of tyrosine phosphor... |
| GO:0043249 | erythrocyte maturation |
| GO:0043496 | regulation of protein homodimerization a... |
| GO:0048662 | negative regulation of smooth muscle cel... |
| GO:0000122 | negative regulation of transcription fro... |
| GO:0030308 | negative regulation of cell growth |
| GO:0045893 | positive regulation of transcription, DN... |
| GO:0006289 | nucleotide-excision repair |
| GO:0002347 | response to tumor cell |
| GO:0006983 | ER overload response |
| GO:0008635 | caspase activation via cytochrome c |
| GO:0009954 | proximal/distal pattern formation |
| GO:0010224 | response to UV-B |
| GO:0035116 | embryonic hindlimb morphogenesis |
| GO:0042177 | negative regulation of protein catabolic... |
| GO:0042791 | 5S class rRNA transcription |
| GO:0042797 | tRNA transcription from RNA polymerase I... |
| GO:0045669 | positive regulation of osteoblast differ... |

1. GO processes affected at medium dose
   - 1. GO processes affected by Benzo[a]fluoranthene

| **GO.ID** | **Term** |
| --- | --- |
| GO:0043687 | post-translational protein modification |
| GO:0016310 | phosphorylation |
| GO:0000389 | nuclear mRNA 3'-splice site recognition |
| GO:0001808 | negative regulation of type IV hypersens... |
| GO:0045060 | negative thymic T cell selection |
| GO:0045226 | extracellular polysaccharide biosyntheti... |
| GO:0016481 | negative regulation of transcription |
| GO:0019885 | antigen processing and presentation of e... |
| GO:0043666 | regulation of phosphoprotein phosphatase... |
| GO:0006556 | S-adenosylmethionine biosynthetic proces... |
| GO:0006601 | creatine biosynthetic process |
| GO:0050823 | peptide antigen stabilization |
| GO:0008624 | induction of apoptosis by extracellular ... |
| GO:0035088 | establishment and/or maintenance of apic... |
| GO:0007569 | cell aging |
| GO:0000398 | nuclear mRNA splicing, via spliceosome |
| GO:0045786 | negative regulation of cell cycle |
| GO:0012501 | programmed cell death |
| GO:0001562 | response to protozoan |
| GO:0006390 | transcription from mitochondrial promote... |
| GO:0042535 | positive regulation of tumor necrosis fa... |
| GO:0048662 | negative regulation of smooth muscle cel... |
| GO:0008635 | caspase activation via cytochrome c |
| GO:0031295 | T cell costimulation |
| GO:0045995 | regulation of embryonic development |
| GO:0046902 | regulation of mitochondrial membrane per... |
| GO:0007243 | protein kinase cascade |
| GO:0032989 | cellular structure morphogenesis |
| GO:0006275 | regulation of DNA replication |
| GO:0046474 | glycerophospholipid biosynthetic process |
| GO:0045941 | positive regulation of transcription |
| GO:0016567 | protein ubiquitination |
| GO:0000059 | protein import into nucleus, docking |
| GO:0048193 | Golgi vesicle transport |
| GO:0006596 | polyamine biosynthetic process |
| GO:0050688 | regulation of defense response to virus |
| GO:0051168 | nuclear export |
| GO:0042733 | embryonic digit morphogenesis |
| GO:0001711 | endodermal cell fate commitment |
| GO:0001922 | B-1 B cell homeostasis |
| GO:0001988 | positive regulation of heart rate in bar... |
| GO:0006256 | UDP catabolic process |
| GO:0006450 | regulation of translational fidelity |
| GO:0006843 | mitochondrial citrate transport |
| GO:0009051 | pentose-phosphate shunt, oxidative branc... |
| GO:0014061 | regulation of norepinephrine secretion |
| GO:0015976 | carbon utilization |
| GO:0018009 | N-terminal peptidyl-L-cysteine N-palmito... |
| GO:0018144 | RNA-protein covalent cross-linking |
| GO:0019322 | pentose biosynthetic process |
| GO:0019408 | dolichol biosynthetic process |
| GO:0030858 | positive regulation of epithelial cell d... |
| GO:0032469 | endoplasmic reticulum calcium ion homeos... |
| GO:0045023 | G0 to G1 transition |
| GO:0045175 | basal protein localization |
| GO:0048149 | behavioral response to ethanol |
| GO:0051097 | negative regulation of helicase activity |
| GO:0060112 | generation of ovulation cycle rhythm |
| GO:0007050 | cell cycle arrest |
| GO:0006461 | protein complex assembly |
| GO:0030155 | regulation of cell adhesion |
| GO:0006303 | double-strand break repair via nonhomolo... |
| GO:0007176 | regulation of epidermal growth factor re... |
| GO:0051881 | regulation of mitochondrial membrane pot... |
| GO:0009615 | response to virus |
| GO:0016571 | histone methylation |
| GO:0042130 | negative regulation of T cell proliferat... |
| GO:0051492 | regulation of stress fiber formation |
| GO:0006886 | intracellular protein transport |
| GO:0001569 | patterning of blood vessels |
| GO:0001953 | negative regulation of cell-matrix adhes... |
| GO:0006284 | base-excision repair |
| GO:0001843 | neural tube closure |
| GO:0042417 | dopamine metabolic process |
| GO:0001667 | ameboidal cell migration |
| GO:0001706 | endoderm formation |
| GO:0006438 | valyl-tRNA aminoacylation |
| GO:0006552 | leucine catabolic process |
| GO:0006679 | glucosylceramide biosynthetic process |
| GO:0006768 | biotin metabolic process |
| GO:0010259 | multicellular organismal aging |
| GO:0014010 | Schwann cell proliferation |
| GO:0015827 | tryptophan transport |
| GO:0019478 | D-amino acid catabolic process |
| GO:0030187 | melatonin biosynthetic process |
| GO:0030951 | establishment and/or maintenance of micr... |
| GO:0032007 | negative regulation of TOR signaling pat... |
| GO:0032225 | regulation of synaptic transmission, dop... |
| GO:0032720 | negative regulation of tumor necrosis fa... |
| GO:0042524 | negative regulation of tyrosine phosphor... |
| GO:0046080 | dUTP metabolic process |
| GO:0051300 | spindle pole body organization and bioge... |
| GO:0051451 | myoblast migration |
| GO:0051894 | positive regulation of focal adhesion fo... |
| GO:0031647 | regulation of protein stability |
| GO:0045792 | negative regulation of cell size |
| GO:0006260 | DNA replication |
| GO:0008283 | cell proliferation |
| GO:0007219 | Notch signaling pathway |
| GO:0009953 | dorsal/ventral pattern formation |

1. GO processes affected at high dose
   - 1. GO processes affected by Styrene

| **GO.ID** | **Term** |
| --- | --- |
| GO:0043687 | post-translational protein modification |
| GO:0000059 | protein import into nucleus, docking |
| GO:0050658 | RNA transport |
| GO:0065002 | intracellular protein transport across a... |
| GO:0001808 | negative regulation of type IV hypersens... |
| GO:0045060 | negative thymic T cell selection |
| GO:0045226 | extracellular polysaccharide biosyntheti... |
| GO:0008632 | apoptotic program |
| GO:0043065 | positive regulation of apoptosis |
| GO:0000209 | protein polyubiquitination |
| GO:0043666 | regulation of phosphoprotein phosphatase... |
| GO:0006275 | regulation of DNA replication |
| GO:0006556 | S-adenosylmethionine biosynthetic proces... |
| GO:0007243 | protein kinase cascade |
| GO:0006289 | nucleotide-excision repair |
| GO:0042752 | regulation of circadian rhythm |
| GO:0051168 | nuclear export |
| GO:0001562 | response to protozoan |
| GO:0042535 | positive regulation of tumor necrosis fa... |
| GO:0031295 | T cell costimulation |
| GO:0046902 | regulation of mitochondrial membrane per... |
| GO:0006915 | apoptosis |
| GO:0006511 | ubiquitin-dependent protein catabolic pr... |
| GO:0046474 | glycerophospholipid biosynthetic process |
| GO:0045786 | negative regulation of cell cycle |
| GO:0022402 | cell cycle process |
| GO:0006461 | protein complex assembly |
| GO:0000389 | nuclear mRNA 3'-splice site recognition |
| GO:0006260 | DNA replication |
| GO:0045792 | negative regulation of cell size |
| GO:0050688 | regulation of defense response to virus |
| GO:0002474 | antigen processing and presentation of p... |
| GO:0051329 | interphase of mitotic cell cycle |
| GO:0001302 | replicative cell aging |
| GO:0001711 | endodermal cell fate commitment |
| GO:0001922 | B-1 B cell homeostasis |
| GO:0001988 | positive regulation of heart rate in bar... |
| GO:0006450 | regulation of translational fidelity |
| GO:0006843 | mitochondrial citrate transport |
| GO:0009051 | pentose-phosphate shunt, oxidative branc... |
| GO:0014061 | regulation of norepinephrine secretion |
| GO:0015855 | pyrimidine transport |
| GO:0016480 | negative regulation of transcription fro... |
| GO:0018144 | RNA-protein covalent cross-linking |
| GO:0019322 | pentose biosynthetic process |
| GO:0019408 | dolichol biosynthetic process |
| GO:0030858 | positive regulation of epithelial cell d... |
| GO:0032469 | endoplasmic reticulum calcium ion homeos... |
| GO:0045023 | G0 to G1 transition |
| GO:0045175 | basal protein localization |
| GO:0048149 | behavioral response to ethanol |
| GO:0051097 | negative regulation of helicase activity |
| GO:0060112 | generation of ovulation cycle rhythm |
| GO:0006303 | double-strand break repair via nonhomolo... |
| GO:0000723 | telomere maintenance |
| GO:0050851 | antigen receptor-mediated signaling path... |
| GO:0042130 | negative regulation of T cell proliferat... |
| GO:0051492 | regulation of stress fiber formation |
| GO:0006968 | cellular defense response |
| GO:0000060 | protein import into nucleus, translocati... |
| GO:0006984 | ER-nuclear signaling pathway |
| GO:0001569 | patterning of blood vessels |
| GO:0015986 | ATP synthesis coupled proton transport |
| GO:0043123 | positive regulation of I-kappaB kinase/N... |
| GO:0043549 | regulation of kinase activity |
| GO:0007163 | establishment and/or maintenance of cell... |
| GO:0006284 | base-excision repair |
| GO:0006378 | mRNA polyadenylation |
| GO:0042417 | dopamine metabolic process |
| GO:0000398 | nuclear mRNA splicing, via spliceosome |
| GO:0031647 | regulation of protein stability |
| GO:0051301 | cell division |
| GO:0001667 | ameboidal cell migration |
| GO:0001706 | endoderm formation |
| GO:0006552 | leucine catabolic process |
| GO:0006565 | L-serine catabolic process |
| GO:0006863 | purine transport |
| GO:0007035 | vacuolar acidification |
| GO:0007387 | anterior compartment specification |
| GO:0007388 | posterior compartment specification |
| GO:0009935 | nutrient import |
| GO:0010259 | multicellular organismal aging |
| GO:0014010 | Schwann cell proliferation |
| GO:0015827 | tryptophan transport |
| GO:0018347 | protein amino acid farnesylation |
| GO:0019478 | D-amino acid catabolic process |
| GO:0030187 | melatonin biosynthetic process |
| GO:0030264 | nuclear fragmentation during apoptosis |
| GO:0030951 | establishment and/or maintenance of micr... |
| GO:0032007 | negative regulation of TOR signaling pat... |
| GO:0032225 | regulation of synaptic transmission, dop... |
| GO:0032720 | negative regulation of tumor necrosis fa... |
| GO:0042524 | negative regulation of tyrosine phosphor... |
| GO:0045329 | carnitine biosynthetic process |
| GO:0046080 | dUTP metabolic process |
| GO:0047496 | vesicle transport along microtubule |
| GO:0050434 | positive regulation of viral transcripti... |
| GO:0051451 | myoblast migration |
| GO:0051894 | positive regulation of focal adhesion fo... |
| GO:0007602 | phototransduction |

- - 1. GO processes affected by Trichloroethylene

| **GO.ID** | **Term** |
| --- | --- |
| GO:0006412 | translation |
| GO:0015986 | ATP synthesis coupled proton transport |
| GO:0006120 | mitochondrial electron transport, NADH t... |
| GO:0006096 | glycolysis |
| GO:0045667 | regulation of osteoblast differentiation |
| GO:0000028 | ribosomal small subunit assembly and mai... |
| GO:0001711 | endodermal cell fate commitment |
| GO:0006425 | glutaminyl-tRNA aminoacylation |
| GO:0008611 | ether lipid biosynthetic process |
| GO:0015853 | adenine transport |
| GO:0019747 | regulation of isoprenoid metabolic proce... |
| GO:0030858 | positive regulation of epithelial cell d... |
| GO:0032287 | myelin maintenance in the peripheral ner... |
| GO:0033081 | regulation of T cell differentiation in ... |
| GO:0035067 | negative regulation of histone acetylati... |
| GO:0045603 | positive regulation of endothelial cell ... |
| GO:0045870 | positive regulation of retroviral genome... |
| GO:0060087 | relaxation of vascular smooth muscle |
| GO:0043066 | negative regulation of apoptosis |
| GO:0006457 | protein folding |
| GO:0006367 | transcription initiation from RNA polyme... |
| GO:0008344 | adult locomotory behavior |
| GO:0007088 | regulation of mitosis |
| GO:0001706 | endoderm formation |
| GO:0001808 | negative regulation of type IV hypersens... |
| GO:0001895 | retinal homeostasis |
| GO:0007023 | post-chaperonin tubulin folding pathway |
| GO:0007387 | anterior compartment specification |
| GO:0007388 | posterior compartment specification |
| GO:0030490 | maturation of SSU-rRNA |
| GO:0030655 | beta-lactam antibiotic catabolic process |
| GO:0043072 | negative regulation of non-apoptotic pro... |
| GO:0045060 | negative thymic T cell selection |
| GO:0045541 | negative regulation of cholesterol biosy... |
| GO:0050665 | hydrogen peroxide biosynthetic process |
| GO:0060088 | auditory receptor cell stereocilium orga... |
| GO:0006801 | superoxide metabolic process |
| GO:0042100 | B cell proliferation |
| GO:0006940 | regulation of smooth muscle contraction |
| GO:0030261 | chromosome condensation |
| GO:0000281 | cytokinesis after mitosis |
| GO:0006107 | oxaloacetate metabolic process |
| GO:0006419 | alanyl-tRNA aminoacylation |
| GO:0006424 | glutamyl-tRNA aminoacylation |
| GO:0007197 | muscarinic acetylcholine receptor, adeny... |
| GO:0009437 | carnitine metabolic process |
| GO:0032331 | negative regulation of chondrocyte diffe... |
| GO:0035104 | positive regulation of sterol regulatory... |
| GO:0040001 | establishment of mitotic spindle localiz... |
| GO:0045663 | positive regulation of myoblast differen... |
| GO:0050823 | peptide antigen stabilization |
| GO:0051302 | regulation of cell division |
| GO:0051725 | protein amino acid de-ADP-ribosylation |
| GO:0042542 | response to hydrogen peroxide |
| GO:0030168 | platelet activation |
| GO:0002262 | myeloid cell homeostasis |
| GO:0006189 | 'de novo' IMP biosynthetic process |
| GO:0006434 | seryl-tRNA aminoacylation |
| GO:0007207 | muscarinic acetylcholine receptor, phosp... |
| GO:0015871 | choline transport |
| GO:0035117 | embryonic arm morphogenesis |
| GO:0042059 | negative regulation of epidermal growth ... |
| GO:0046620 | regulation of organ growth |
| GO:0048268 | clathrin cage assembly |
| GO:0051583 | dopamine uptake |
| GO:0006099 | tricarboxylic acid cycle |
| GO:0042254 | ribosome biogenesis and assembly |
| GO:0007190 | activation of adenylate cyclase activity |
| GO:0045892 | negative regulation of transcription, DN... |
| GO:0007283 | spermatogenesis |
| GO:0019883 | antigen processing and presentation of e... |
| GO:0000303 | response to superoxide |
| GO:0001562 | response to protozoan |
| GO:0006102 | isocitrate metabolic process |
| GO:0006269 | DNA replication, synthesis of RNA primer |
| GO:0007025 | beta-tubulin folding |
| GO:0007184 | SMAD protein nuclear translocation |
| GO:0009950 | dorsal/ventral axis specification |
| GO:0042535 | positive regulation of tumor necrosis fa... |
| GO:0043030 | regulation of macrophage activation |
| GO:0046580 | negative regulation of Ras protein signa... |
| GO:0048662 | negative regulation of smooth muscle cel... |
| GO:0006986 | response to unfolded protein |
| GO:0006357 | regulation of transcription from RNA pol... |
| GO:0009062 | fatty acid catabolic process |
| GO:0006108 | malate metabolic process |
| GO:0009954 | proximal/distal pattern formation |
| GO:0031295 | T cell costimulation |
| GO:0035116 | embryonic hindlimb morphogenesis |
| GO:0046716 | muscle maintenance |
| GO:0046836 | glycolipid transport |
| GO:0048678 | response to axon injury |
| GO:0051496 | positive regulation of stress fiber form... |
| GO:0060052 | neurofilament cytoskeleton organization ... |
| GO:0060070 | Wnt receptor signaling pathway through b... |
| GO:0030308 | negative regulation of cell growth |
| GO:0000398 | nuclear mRNA splicing, via spliceosome |
| GO:0043123 | positive regulation of I-kappaB kinase/N... |
| GO:0007162 | negative regulation of cell adhesion |
| GO:0008624 | induction of apoptosis by extracellular ... |

- - 1. GO processes affected by Benz[a]anthracene

| **GO.ID** | **Term** |
| --- | --- |
| GO:0050823 | peptide antigen stabilization |
| GO:0002474 | antigen processing and presentation of p... |
| GO:0042542 | response to hydrogen peroxide |
| GO:0042531 | positive regulation of tyrosine phosphor... |
| GO:0006563 | L-serine metabolic process |
| GO:0031016 | pancreas development |
| GO:0030521 | androgen receptor signaling pathway |
| GO:0006890 | retrograde vesicle-mediated transport, G... |
| GO:0001711 | endodermal cell fate commitment |
| GO:0001916 | positive regulation of T cell mediated c... |
| GO:0006450 | regulation of translational fidelity |
| GO:0006990 | positive regulation of gene-specific tra... |
| GO:0030858 | positive regulation of epithelial cell d... |
| GO:0032510 | endosome to lysosome transport via multi... |
| GO:0033993 | response to lipid |
| GO:0042789 | mRNA transcription from RNA polymerase I... |
| GO:0045019 | negative regulation of nitric oxide bios... |
| GO:0045837 | negative regulation of membrane potentia... |
| GO:0006544 | glycine metabolic process |
| GO:0006687 | glycosphingolipid metabolic process |
| GO:0001706 | endoderm formation |
| GO:0001808 | negative regulation of type IV hypersens... |
| GO:0006290 | pyrimidine dimer repair |
| GO:0006679 | glucosylceramide biosynthetic process |
| GO:0007095 | mitotic cell cycle G2/M transition DNA d... |
| GO:0009223 | pyrimidine deoxyribonucleotide catabolic... |
| GO:0019509 | methionine salvage |
| GO:0030655 | beta-lactam antibiotic catabolic process |
| GO:0030836 | positive regulation of actin filament de... |
| GO:0032780 | negative regulation of ATPase activity |
| GO:0032792 | inhibition of CREB transcription factor |
| GO:0032816 | positive regulation of natural killer ce... |
| GO:0045060 | negative thymic T cell selection |
| GO:0046477 | glycosylceramide catabolic process |
| GO:0046855 | inositol phosphate dephosphorylation |
| GO:0046856 | phosphoinositide dephosphorylation |
| GO:0048007 | antigen processing and presentation, exo... |
| GO:0051138 | positive regulation of NK T cell differe... |
| GO:0051898 | negative regulation of protein kinase B ... |
| GO:0016071 | mRNA metabolic process |
| GO:0000079 | regulation of cyclin-dependent protein k... |
| GO:0051607 | defense response to virus |
| GO:0030168 | platelet activation |
| GO:0042102 | positive regulation of T cell proliferat... |
| GO:0007568 | aging |
| GO:0007067 | mitosis |
| GO:0002860 | positive regulation of natural killer ce... |
| GO:0009440 | cyanate catabolic process |
| GO:0032331 | negative regulation of chondrocyte diffe... |
| GO:0048008 | platelet-derived growth factor receptor ... |
| GO:0001975 | response to amphetamine |
| GO:0006654 | phosphatidic acid biosynthetic process |
| GO:0008634 | negative regulation of survival gene pro... |
| GO:0018345 | protein palmitoylation |
| GO:0035117 | embryonic arm morphogenesis |
| GO:0051895 | negative regulation of focal adhesion fo... |
| GO:0045941 | positive regulation of transcription |
| GO:0001562 | response to protozoan |
| GO:0006269 | DNA replication, synthesis of RNA primer |
| GO:0006390 | transcription from mitochondrial promote... |
| GO:0006622 | protein targeting to lysosome |
| GO:0009950 | dorsal/ventral axis specification |
| GO:0032729 | positive regulation of interferon-gamma ... |
| GO:0032781 | positive regulation of ATPase activity |
| GO:0042535 | positive regulation of tumor necrosis fa... |
| GO:0051001 | negative regulation of nitric-oxide synt... |
| GO:0000910 | cytokinesis |
| GO:0006983 | ER overload response |
| GO:0009954 | proximal/distal pattern formation |
| GO:0010224 | response to UV-B |
| GO:0031295 | T cell costimulation |
| GO:0032496 | response to lipopolysaccharide |
| GO:0035116 | embryonic hindlimb morphogenesis |
| GO:0045669 | positive regulation of osteoblast differ... |
| GO:0046902 | regulation of mitochondrial membrane per... |
| GO:0051014 | actin filament severing |
| GO:0060070 | Wnt receptor signaling pathway through b... |
| GO:0045671 | negative regulation of osteoclast differ... |
| GO:0050830 | defense response to Gram-positive bacter... |
| GO:0002831 | regulation of response to biotic stimulu... |
| GO:0006777 | Mo-molybdopterin cofactor biosynthetic p... |
| GO:0009086 | methionine biosynthetic process |
| GO:0009409 | response to cold |
| GO:0046677 | response to antibiotic |
| GO:0006355 | regulation of transcription, DNA-depende... |
| GO:0006325 | establishment and/or maintenance of chro... |
| GO:0008360 | regulation of cell shape |
| GO:0042733 | embryonic digit morphogenesis |
| GO:0048738 | cardiac muscle development |
| GO:0051592 | response to calcium ion |
| GO:0030049 | muscle filament sliding |
| GO:0032367 | intracellular cholesterol transport |
| GO:0055010 | ventricular cardiac muscle morphogenesis |
| GO:0030503 | regulation of cell redox homeostasis |
| GO:0042130 | negative regulation of T cell proliferat... |
| GO:0050709 | negative regulation of protein secretion |
| GO:0006916 | anti-apoptosis |
| GO:0008283 | cell proliferation |
| GO:0001569 | patterning of blood vessels |
| GO:0001837 | epithelial to mesenchymal transition |
