# Supplementary material for: Effect of Chemical Mutagens and Carcinogens on Gene Expression Profiles in Human TK6 Cells
Source: PLoS One. 2012 Jun 18;7(6):e39205. doi: 10.1371/journal.pone.0039205 (PMC3377624; doi:10.1371/journal.pone.0039205)
Supplement: Table S4 — Functional classification of significantly altered genes with dose-response trend by exposure to carcinogens (S9-) into gene ontology (GO) categories. (DOCX) [file pone.0039205.s004.docx]

**Supplementary Table S4: Functional classification of significantly altered genes with dose-response trend by exposure to carcinogens (S9-) into gene ontology (GO) categories.**

- 1. GO process affected by Benz[a]anthracene

| GO.ID | Term |
| --- | --- |
| GO:0051246 | regulation of protein metabolic process |
| GO:0006512 | ubiquitin cycle |
| GO:0007399 | nervous system development |
| GO:0006139 | nucleobase, nucleoside, nucleotide and n... |
| GO:0030154 | cell differentiation |
| GO:0006355 | regulation of transcription, DNA-depende... |
| GO:0007165 | signal transduction |
| GO:0006350 | transcription |
| GO:0006351 | transcription, DNA-dependent |
| GO:0006464 | protein modification process |
| GO:0007154 | cell communication |
| GO:0007275 | multicellular organismal development |
| GO:0008150 | biological_process |
| GO:0008152 | metabolic process |
| GO:0009987 | cellular process |
| GO:0010467 | gene expression |
| GO:0010468 | regulation of gene expression |
| GO:0016070 | RNA metabolic process |
| GO:0019219 | regulation of nucleobase, nucleoside, nu... |
| GO:0019222 | regulation of metabolic process |
| GO:0019538 | protein metabolic process |
| GO:0031323 | regulation of cellular metabolic process |
| GO:0032501 | multicellular organismal process |
| GO:0032502 | developmental process |
| GO:0032774 | RNA biosynthetic process |
| GO:0043170 | macromolecule metabolic process |
| GO:0043283 | biopolymer metabolic process |
| GO:0043412 | biopolymer modification |
| GO:0043687 | post-translational protein modification |
| GO:0044237 | cellular metabolic process |
| GO:0044238 | primary metabolic process |
| GO:0044260 | cellular macromolecule metabolic process |
| GO:0044267 | cellular protein metabolic process |
| GO:0045449 | regulation of transcription |
| GO:0048731 | system development |
| GO:0048856 | anatomical structure development |
| GO:0048869 | cellular developmental process |
| GO:0050789 | regulation of biological process |
| GO:0050794 | regulation of cellular process |
| GO:0051252 | regulation of RNA metabolic process |
| GO:0065007 | biological regulation |
| GO:0000002 | mitochondrial genome maintenance |
| GO:0000003 | reproduction |
| GO:0000012 | single strand break repair |
| GO:0000018 | regulation of DNA recombination |
| GO:0000019 | regulation of mitotic recombination |
| GO:0000022 | mitotic spindle elongation |
| GO:0000023 | maltose metabolic process |
| GO:0000028 | ribosomal small subunit assembly and mai... |
| GO:0000038 | very-long-chain fatty acid metabolic pro... |
| GO:0000041 | transition metal ion transport |
| GO:0000042 | protein targeting to Golgi |
| GO:0000045 | autophagic vacuole formation |
| GO:0000050 | urea cycle |
| GO:0000051 | urea cycle intermediate metabolic proces... |
| GO:0000052 | citrulline metabolic process |
| GO:0000053 | argininosuccinate metabolic process |
| GO:0000059 | protein import into nucleus, docking |
| GO:0000060 | protein import into nucleus, translocati... |
| GO:0000066 | mitochondrial ornithine transport |
| GO:0000070 | mitotic sister chromatid segregation |
| GO:0000072 | M phase specific microtubule process |
| GO:0000075 | cell cycle checkpoint |
| GO:0000076 | DNA replication checkpoint |
| GO:0000077 | DNA damage checkpoint |
| GO:0000079 | regulation of cyclin-dependent protein k... |
| GO:0000080 | G1 phase of mitotic cell cycle |
| GO:0000082 | G1/S transition of mitotic cell cycle |
| GO:0000083 | G1/S-specific transcription in mitotic c... |
| GO:0000084 | S phase of mitotic cell cycle |
| GO:0000085 | G2 phase of mitotic cell cycle |
| GO:0000086 | G2/M transition of mitotic cell cycle |
| GO:0000087 | M phase of mitotic cell cycle |
| GO:0000089 | mitotic metaphase |
| GO:0000090 | mitotic anaphase |
| GO:0000093 | mitotic telophase |
| GO:0000096 | sulfur amino acid metabolic process |
| GO:0000097 | sulfur amino acid biosynthetic process |
| GO:0000098 | sulfur amino acid catabolic process |
| GO:0000101 | sulfur amino acid transport |
| GO:0000103 | sulfate assimilation |
| GO:0000105 | histidine biosynthetic process |
| GO:0000114 | G1-specific transcription in mitotic cel... |
| GO:0000115 | S-phase-specific transcription in mitoti... |
| GO:0000122 | negative regulation of transcription fro... |
| GO:0000132 | establishment of mitotic spindle orienta... |
| GO:0000154 | rRNA modification |
| GO:0000160 | two-component signal transduction system... |
| GO:0000161 | MAPKKK cascade during osmolarity sensing |
| GO:0000165 | MAPKKK cascade |
| GO:0000173 | inactivation of MAPK activity during osm... |
| GO:0000183 | chromatin silencing at rDNA |
| GO:0000184 | mRNA catabolic process, nonsense-mediate... |
| GO:0000185 | activation of MAPKKK activity |
| GO:0000186 | activation of MAPKK activity |
| GO:0000187 | activation of MAPK activity |
| GO:0000188 | inactivation of MAPK activity |
| GO:0000209 | protein polyubiquitination |
| GO:0000212 | meiotic spindle organization and biogene... |
| GO:0000226 | microtubule cytoskeleton organization an... |

- 1. GO process affected by Benzene

| **GO.ID** | **Term** |
| --- | --- |
| GO:0019049 | evasion of host defenses by virus |
| GO:0045730 | respiratory burst |
| GO:0007183 | SMAD protein complex assembly |
| GO:0032909 | regulation of transforming growth factor... |
| GO:0042921 | glucocorticoid receptor signaling pathwa... |
| GO:0045944 | positive regulation of transcription fro... |
| GO:0042993 | positive regulation of transcription fac... |
| GO:0016180 | snRNA processing |
| GO:0016575 | histone deacetylation |
| GO:0030520 | estrogen receptor signaling pathway |
| GO:0045930 | negative regulation of mitotic cell cycl... |
| GO:0006917 | induction of apoptosis |
| GO:0007528 | neuromuscular junction development |
| GO:0017015 | regulation of transforming growth factor... |
| GO:0006958 | complement activation, classical pathway |
| GO:0006919 | caspase activation |
| GO:0006944 | membrane fusion |
| GO:0001666 | response to hypoxia |
| GO:0030308 | negative regulation of cell growth |
| GO:0007204 | elevation of cytosolic calcium ion conce... |
| GO:0009725 | response to hormone stimulus |
| GO:0007050 | cell cycle arrest |
| GO:0045935 | positive regulation of nucleobase, nucle... |
| GO:0006310 | DNA recombination |
| GO:0045087 | innate immune response |
| GO:0006816 | calcium ion transport |
| GO:0000122 | negative regulation of transcription fro... |
| GO:0006457 | protein folding |
| GO:0006260 | DNA replication |
| GO:0008285 | negative regulation of cell proliferatio... |
| GO:0006281 | DNA repair |
| GO:0006886 | intracellular protein transport |
| GO:0009887 | organ morphogenesis |
| GO:0006955 | immune response |
| GO:0016192 | vesicle-mediated transport |
| GO:0006512 | ubiquitin cycle |
| GO:0051252 | regulation of RNA metabolic process |
| GO:0006351 | transcription, DNA-dependent |
| GO:0000003 | reproduction |
| GO:0000278 | mitotic cell cycle |
| GO:0001558 | regulation of cell growth |
| GO:0001816 | cytokine production |
| GO:0001817 | regulation of cytokine production |
| GO:0002250 | adaptive immune response |
| GO:0002252 | immune effector process |
| GO:0002253 | activation of immune response |
| GO:0002376 | immune system process |
| GO:0002443 | leukocyte mediated immunity |
| GO:0002449 | lymphocyte mediated immunity |
| GO:0002455 | humoral immune response mediated by circ... |
| GO:0002460 | adaptive immune response based on somati... |
| GO:0002526 | acute inflammatory response |
| GO:0002541 | activation of plasma proteins during acu... |
| GO:0002682 | regulation of immune system process |
| GO:0002684 | positive regulation of immune system pro... |
| GO:0006139 | nucleobase, nucleoside, nucleotide and n... |
| GO:0006259 | DNA metabolic process |
| GO:0006325 | establishment and/or maintenance of chro... |
| GO:0006350 | transcription |
| GO:0006355 | regulation of transcription, DNA-depende... |
| GO:0006357 | regulation of transcription from RNA pol... |
| GO:0006366 | transcription from RNA polymerase II pro... |
| GO:0006396 | RNA processing |
| GO:0006461 | protein complex assembly |
| GO:0006464 | protein modification process |
| GO:0006476 | protein amino acid deacetylation |
| GO:0006508 | proteolysis |
| GO:0006605 | protein targeting |
| GO:0006606 | protein import into nucleus |
| GO:0006810 | transport |
| GO:0006811 | ion transport |
| GO:0006812 | cation transport |
| GO:0006873 | cellular ion homeostasis |
| GO:0006874 | cellular calcium ion homeostasis |
| GO:0006875 | cellular metal ion homeostasis |
| GO:0006913 | nucleocytoplasmic transport |
| GO:0006915 | apoptosis |
| GO:0006950 | response to stress |
| GO:0006952 | defense response |
| GO:0006954 | inflammatory response |
| GO:0006956 | complement activation |
| GO:0006959 | humoral immune response |
| GO:0006974 | response to DNA damage stimulus |
| GO:0006996 | organelle organization and biogenesis |
| GO:0007049 | cell cycle |
| GO:0007154 | cell communication |
| GO:0007165 | signal transduction |
| GO:0007166 | cell surface receptor linked signal tran... |
| GO:0007167 | enzyme linked receptor protein signaling... |
| GO:0007178 | transmembrane receptor protein serine/th... |
| GO:0007179 | transforming growth factor beta receptor... |
| GO:0007242 | intracellular signaling cascade |
| GO:0007275 | multicellular organismal development |
| GO:0007346 | regulation of mitotic cell cycle |
| GO:0007517 | muscle development |
| GO:0007519 | skeletal muscle development |
| GO:0008104 | protein localization |
| GO:0008150 | biological_process |
| GO:0008152 | metabolic process |
| GO:0008219 | cell death |

- 1. GO process affected by Epichlorohydrin

| **GO.ID** | **Term** |
| --- | --- |
| GO:0006412 | translation |
| GO:0006509 | membrane protein ectodomain proteolysis |
| GO:0007220 | Notch receptor processing |
| GO:0006446 | regulation of translational initiation |
| GO:0008624 | induction of apoptosis by extracellular ... |
| GO:0001808 | negative regulation of type IV hypersens... |
| GO:0045060 | negative thymic T cell selection |
| GO:0006596 | polyamine biosynthetic process |
| GO:0007050 | cell cycle arrest |
| GO:0009070 | serine family amino acid biosynthetic pr... |
| GO:0042987 | amyloid precursor protein catabolic proc... |
| GO:0048168 | regulation of neuronal synaptic plastici... |
| GO:0016458 | gene silencing |
| GO:0042981 | regulation of apoptosis |
| GO:0001562 | response to protozoan |
| GO:0008615 | pyridoxine biosynthetic process |
| GO:0042535 | positive regulation of tumor necrosis fa... |
| GO:0043249 | erythrocyte maturation |
| GO:0050671 | positive regulation of lymphocyte prolif... |
| GO:0031274 | positive regulation of pseudopodium form... |
| GO:0031295 | T cell costimulation |
| GO:0046834 | lipid phosphorylation |
| GO:0007163 | establishment and/or maintenance of cell... |
| GO:0050688 | regulation of defense response to virus |
| GO:0018279 | protein amino acid N-linked glycosylatio... |
| GO:0000398 | nuclear mRNA splicing, via spliceosome |
| GO:0000028 | ribosomal small subunit assembly and mai... |
| GO:0001582 | detection of chemical stimulus involved ... |
| GO:0001711 | endodermal cell fate commitment |
| GO:0006450 | regulation of translational fidelity |
| GO:0006851 | mitochondrial calcium ion transport |
| GO:0007403 | glial cell fate determination |
| GO:0008049 | male courtship behavior |
| GO:0008611 | ether lipid biosynthetic process |
| GO:0009229 | thiamin diphosphate biosynthetic process |
| GO:0015855 | pyrimidine transport |
| GO:0019747 | regulation of isoprenoid metabolic proce... |
| GO:0019853 | L-ascorbic acid biosynthetic process |
| GO:0030828 | positive regulation of cGMP biosynthetic... |
| GO:0030858 | positive regulation of epithelial cell d... |
| GO:0031293 | membrane protein intracellular domain pr... |
| GO:0032417 | positive regulation of sodium:hydrogen a... |
| GO:0032469 | endoplasmic reticulum calcium ion homeos... |
| GO:0042789 | mRNA transcription from RNA polymerase I... |
| GO:0042840 | D-glucuronate catabolic process |
| GO:0043071 | positive regulation of non-apoptotic pro... |
| GO:0046185 | aldehyde catabolic process |
| GO:0048312 | intracellular distribution of mitochondr... |
| GO:0051790 | short-chain fatty acid biosynthetic proc... |
| GO:0051792 | medium-chain fatty acid biosynthetic pro... |
| GO:0045454 | cell redox homeostasis |
| GO:0042325 | regulation of phosphorylation |
| GO:0030036 | actin cytoskeleton organization and biog... |
| GO:0042088 | T-helper 1 type immune response |
| GO:0042130 | negative regulation of T cell proliferat... |
| GO:0030198 | extracellular matrix organization and bi... |
| GO:0001706 | endoderm formation |
| GO:0001835 | blastocyst hatching |
| GO:0002268 | follicular dendritic cell differentiatio... |
| GO:0006222 | UMP biosynthetic process |
| GO:0006346 | methylation-dependent chromatin silencin... |
| GO:0006438 | valyl-tRNA aminoacylation |
| GO:0006608 | snRNP protein import into nucleus |
| GO:0006627 | mitochondrial protein processing during ... |
| GO:0006679 | glucosylceramide biosynthetic process |
| GO:0006863 | purine transport |
| GO:0006987 | unfolded protein response, activation of... |
| GO:0009313 | oligosaccharide catabolic process |
| GO:0009935 | nutrient import |
| GO:0014010 | Schwann cell proliferation |
| GO:0015788 | UDP-N-acetylglucosamine transport |
| GO:0015827 | tryptophan transport |
| GO:0030208 | dermatan sulfate biosynthetic process |
| GO:0030490 | maturation of SSU-rRNA |
| GO:0031333 | negative regulation of protein complex a... |
| GO:0032792 | inhibition of CREB transcription factor |
| GO:0035090 | maintenance of apical/basal cell polarit... |
| GO:0042524 | negative regulation of tyrosine phosphor... |
| GO:0043537 | negative regulation of blood vessel endo... |
| GO:0046022 | positive regulation of transcription fro... |
| GO:0046080 | dUTP metabolic process |
| GO:0046534 | positive regulation of photoreceptor cel... |
| GO:0048549 | positive regulation of pinocytosis |
| GO:0051000 | positive regulation of nitric-oxide synt... |
| GO:0051451 | myoblast migration |
| GO:0001522 | pseudouridine synthesis |
| GO:0007004 | telomere maintenance via telomerase |
| GO:0006783 | heme biosynthetic process |
| GO:0050819 | negative regulation of coagulation |
| GO:0006270 | DNA replication initiation |
| GO:0006953 | acute-phase response |
| GO:0045786 | negative regulation of cell cycle |
| GO:0001958 | endochondral ossification |
| GO:0006424 | glutamyl-tRNA aminoacylation |
| GO:0006772 | thiamin metabolic process |
| GO:0007080 | mitotic metaphase plate congression |
| GO:0007097 | nuclear migration |
| GO:0007175 | negative regulation of epidermal growth ... |
| GO:0009440 | cyanate catabolic process |
| GO:0010155 | regulation of proton transport |

- 1. GO process affected by Hydroquinone

| **GO.ID** | **Term** |
| --- | --- |
| GO:0015961 | diadenosine polyphosphate catabolic proc... |
| GO:0050823 | peptide antigen stabilization |
| GO:0009650 | UV protection |
| GO:0033683 | nucleotide-excision repair, DNA incision |
| GO:0006283 | transcription-coupled nucleotide-excisio... |
| GO:0019885 | antigen processing and presentation of e... |
| GO:0016180 | snRNA processing |
| GO:0006890 | retrograde vesicle-mediated transport, G... |
| GO:0006120 | mitochondrial electron transport, NADH t... |
| GO:0019722 | calcium-mediated signaling |
| GO:0006118 | electron transport |
| GO:0045935 | positive regulation of nucleobase, nucle... |
| GO:0008360 | regulation of cell shape |
| GO:0009913 | epidermal cell differentiation |
| GO:0046777 | protein amino acid autophosphorylation |
| GO:0042113 | B cell activation |
| GO:0000075 | cell cycle checkpoint |
| GO:0006119 | oxidative phosphorylation |
| GO:0007243 | protein kinase cascade |
| GO:0006979 | response to oxidative stress |
| GO:0043087 | regulation of GTPase activity |
| GO:0009615 | response to virus |
| GO:0043123 | positive regulation of I-kappaB kinase/N... |
| GO:0045449 | regulation of transcription |
| GO:0008544 | epidermis development |
| GO:0006350 | transcription |
| GO:0045944 | positive regulation of transcription fro... |
| GO:0007249 | I-kappaB kinase/NF-kappaB cascade |
| GO:0006955 | immune response |
| GO:0007398 | ectoderm development |
| GO:0007605 | sensory perception of sound |
| GO:0010468 | regulation of gene expression |
| GO:0000165 | MAPKKK cascade |
| GO:0006091 | generation of precursor metabolites and ... |
| GO:0007017 | microtubule-based process |
| GO:0002376 | immune system process |
| GO:0001775 | cell activation |
| GO:0006917 | induction of apoptosis |
| GO:0006915 | apoptosis |
| GO:0012501 | programmed cell death |
| GO:0007242 | intracellular signaling cascade |
| GO:0006355 | regulation of transcription, DNA-depende... |
| GO:0051252 | regulation of RNA metabolic process |
| GO:0045859 | regulation of protein kinase activity |
| GO:0009607 | response to biotic stimulus |
| GO:0008219 | cell death |
| GO:0006351 | transcription, DNA-dependent |
| GO:0032774 | RNA biosynthetic process |
| GO:0006139 | nucleobase, nucleoside, nucleotide and n... |
| GO:0000902 | cell morphogenesis |
| GO:0016337 | cell-cell adhesion |
| GO:0006461 | protein complex assembly |
| GO:0006954 | inflammatory response |
| GO:0006793 | phosphorus metabolic process |
| GO:0006796 | phosphate metabolic process |
| GO:0009611 | response to wounding |
| GO:0006396 | RNA processing |
| GO:0006357 | regulation of transcription from RNA pol... |
| GO:0007264 | small GTPase mediated signal transductio... |
| GO:0007010 | cytoskeleton organization and biogenesis |
| GO:0006952 | defense response |
| GO:0007154 | cell communication |
| GO:0009605 | response to external stimulus |
| GO:0007267 | cell-cell signaling |
| GO:0006366 | transcription from RNA polymerase II pro... |
| GO:0015031 | protein transport |
| GO:0006468 | protein amino acid phosphorylation |
| GO:0006508 | proteolysis |
| GO:0007600 | sensory perception |
| GO:0006810 | transport |
| GO:0008104 | protein localization |
| GO:0007155 | cell adhesion |
| GO:0007049 | cell cycle |
| GO:0051234 | establishment of localization |
| GO:0007165 | signal transduction |
| GO:0006950 | response to stress |
| GO:0008152 | metabolic process |
| GO:0006996 | organelle organization and biogenesis |
| GO:0003008 | system process |
| GO:0006464 | protein modification process |
| GO:0007275 | multicellular organismal development |
| GO:0000737 | DNA catabolic process, endonucleolytic |
| GO:0002474 | antigen processing and presentation of p... |
| GO:0002483 | antigen processing and presentation of e... |
| GO:0006152 | purine nucleoside catabolic process |
| GO:0006259 | DNA metabolic process |
| GO:0006281 | DNA repair |
| GO:0006289 | nucleotide-excision repair |
| GO:0006308 | DNA catabolic process |
| GO:0006518 | peptide metabolic process |
| GO:0006974 | response to DNA damage stimulus |
| GO:0008150 | biological_process |
| GO:0009056 | catabolic process |
| GO:0009057 | macromolecule catabolic process |
| GO:0009116 | nucleoside metabolic process |
| GO:0009119 | ribonucleoside metabolic process |
| GO:0009164 | nucleoside catabolic process |
| GO:0009314 | response to radiation |
| GO:0009411 | response to UV |
| GO:0009416 | response to light stimulus |

- 1. GO process affected by Trichloroethylene

| **GO.ID** | **Term** |
| --- | --- |
| GO:0045941 | positive regulation of transcription |
| GO:0000398 | nuclear mRNA splicing, via spliceosome |
| GO:0031295 | T cell costimulation |
| GO:0001808 | negative regulation of type IV hypersens... |
| GO:0045060 | negative thymic T cell selection |
| GO:0051451 | myoblast migration |
| GO:0000184 | mRNA catabolic process, nonsense-mediate... |
| GO:0030521 | androgen receptor signaling pathway |
| GO:0001562 | response to protozoan |
| GO:0007183 | SMAD protein complex assembly |
| GO:0042535 | positive regulation of tumor necrosis fa... |
| GO:0030036 | actin cytoskeleton organization and biog... |
| GO:0031274 | positive regulation of pseudopodium form... |
| GO:0046902 | regulation of mitochondrial membrane per... |
| GO:0007076 | mitotic chromosome condensation |
| GO:0043687 | post-translational protein modification |
| GO:0030308 | negative regulation of cell growth |
| GO:0000122 | negative regulation of transcription fro... |
| GO:0008624 | induction of apoptosis by extracellular ... |
| GO:0051345 | positive regulation of hydrolase activit... |
| GO:0050688 | regulation of defense response to virus |
| GO:0009116 | nucleoside metabolic process |
| GO:0051301 | cell division |
| GO:0006857 | oligopeptide transport |
| GO:0048538 | thymus development |
| GO:0000290 | deadenylation-dependent decapping |
| GO:0000722 | telomere maintenance via recombination |
| GO:0001711 | endodermal cell fate commitment |
| GO:0001922 | B-1 B cell homeostasis |
| GO:0006450 | regulation of translational fidelity |
| GO:0015855 | pyrimidine transport |
| GO:0018144 | RNA-protein covalent cross-linking |
| GO:0030828 | positive regulation of cGMP biosynthetic... |
| GO:0030858 | positive regulation of epithelial cell d... |
| GO:0031293 | membrane protein intracellular domain pr... |
| GO:0032417 | positive regulation of sodium:hydrogen a... |
| GO:0045023 | G0 to G1 transition |
| GO:0051044 | positive regulation of membrane protein ... |
| GO:0051097 | negative regulation of helicase activity |
| GO:0055009 | atrial cardiac muscle morphogenesis |
| GO:0042102 | positive regulation of T cell proliferat... |
| GO:0042088 | T-helper 1 type immune response |
| GO:0042130 | negative regulation of T cell proliferat... |
| GO:0048704 | embryonic skeletal morphogenesis |
| GO:0007264 | small GTPase mediated signal transductio... |
| GO:0007569 | cell aging |
| GO:0008360 | regulation of cell shape |
| GO:0048168 | regulation of neuronal synaptic plastici... |
| GO:0001543 | ovarian follicle rupture |
| GO:0001706 | endoderm formation |
| GO:0002268 | follicular dendritic cell differentiatio... |
| GO:0006552 | leucine catabolic process |
| GO:0006679 | glucosylceramide biosynthetic process |
| GO:0006863 | purine transport |
| GO:0009935 | nutrient import |
| GO:0015788 | UDP-N-acetylglucosamine transport |
| GO:0019049 | evasion of host defenses by virus |
| GO:0019478 | D-amino acid catabolic process |
| GO:0030208 | dermatan sulfate biosynthetic process |
| GO:0030655 | beta-lactam antibiotic catabolic process |
| GO:0031333 | negative regulation of protein complex a... |
| GO:0035090 | maintenance of apical/basal cell polarit... |
| GO:0043017 | positive regulation of lymphotoxin A bio... |
| GO:0043537 | negative regulation of blood vessel endo... |
| GO:0046080 | dUTP metabolic process |
| GO:0048549 | positive regulation of pinocytosis |
| GO:0051000 | positive regulation of nitric-oxide synt... |
| GO:0006333 | chromatin assembly or disassembly |
| GO:0043407 | negative regulation of MAP kinase activi... |
| GO:0007050 | cell cycle arrest |
| GO:0007163 | establishment and/or maintenance of cell... |
| GO:0006783 | heme biosynthetic process |
| GO:0006270 | DNA replication initiation |
| GO:0006544 | glycine metabolic process |
| GO:0007179 | transforming growth factor beta receptor... |
| GO:0000920 | cell separation during cytokinesis |
| GO:0001958 | endochondral ossification |
| GO:0006196 | AMP catabolic process |
| GO:0006601 | creatine biosynthetic process |
| GO:0007097 | nuclear migration |
| GO:0018076 | N-terminal peptidyl-lysine acetylation |
| GO:0019934 | cGMP-mediated signaling |
| GO:0032331 | negative regulation of chondrocyte diffe... |
| GO:0032801 | receptor catabolic process |
| GO:0035162 | embryonic hemopoiesis |
| GO:0042149 | cellular response to glucose starvation |
| GO:0043011 | myeloid dendritic cell differentiation |
| GO:0045404 | positive regulation of interleukin-4 bio... |
| GO:0045630 | positive regulation of T-helper 2 cell d... |
| GO:0046967 | cytosol to ER transport |
| GO:0006367 | transcription initiation from RNA polyme... |
| GO:0006888 | ER to Golgi vesicle-mediated transport |
| GO:0008286 | insulin receptor signaling pathway |
| GO:0000059 | protein import into nucleus, docking |
| GO:0006047 | UDP-N-acetylglucosamine metabolic proces... |
| GO:0006654 | phosphatidic acid biosynthetic process |
| GO:0007207 | muscarinic acetylcholine receptor, phosp... |
| GO:0008634 | negative regulation of survival gene pro... |
| GO:0016068 | type I hypersensitivity |
| GO:0016246 | RNA interference |
